# Endurant Stents in Abdominal Aortic Aneurysm Repair: A Systematic Review and Meta-Analysis

**DOI:** 10.3390/jcm14186453

**Published:** 2025-09-12

**Authors:** Georgios Loufopoulos, Petroula Nana, Konstantinos Dakis, George Kouvelos, Vladimir Makaloski, Konstantinos P. Donas, Miltiadis Matsagkas, Athanasios Giannoukas, Konstantinos Spanos

**Affiliations:** 1 Department of Vascular Surgery, University Hospital of Lausanne (CHUV), 1005 Lausanne, Switzerland; 2 Department of Vascular Surgery, Faculty of Medicine, School of Health Sciences, University Hospital of Larissa, University of Thessaly, 38221 Larissa, Greecespanos.kon@gmail.com (K.S.); 3 Department of Vascular Surgery, Swiss Aortic Center Bern, University Hospital of Bern, Inselspital, 3010 Bern, Switzerland; 4 Rhein Main Vascular Center, Department of Vascular and Endovascular Surgery, Asklepios Clinics Langen, Paulinen Wiesbaden, 63225 Langen, Germany

**Keywords:** abdominal aortic aneurysm, endovascular treatment, endoleak, aortic stent

## Abstract

**Objectives:** New endografts have improved clinical outcomes in patients with abdominal aortic aneurysm (AAA) treated with the endovascular approach (EVAR). The purpose of this study is to evaluate the Endurant endograft for EVAR. **Methods:** A systematic search was conducted in PubMed, Scopus, and Cochrane for studies including patients treated with EVAR for unruptured AAA. This meta-analysis follows PRISMA guidelines (PROSPERO: CRD42024621517). A Kaplan–Meier-derived individual patient data analysis assessed the survival, the freedom from reintervention, the freedom from type Ia endoleak (ETIa), and the aneurysm-related mortality rates. The analysis reflects aggregated survival data, and the at-risk population decreases over time due to censoring and loss to follow-up. Kaplan–Meier survival curves were digitized to extract survival/mortality values at specific time points, and number-at-risk tables or total events were used to improve time-to-event accuracy. A subgroup analysis compared the outcomes of treatment within versus outside the instructions for use (IFU). **Results:** Twenty-six studies met our eligibility criteria, incorporating 5901 patients in terms of survival, with survival rates at 1, 5, and 10 years of follow-up at 94.4%, 71.6%, and 42.4%, respectively, while overall aneurysm-related mortality rates were 0.8%, 2.3%, and 7.6%, respectively. Freedom from secondary reintervention was 94.9% at 1 year, 81.9% at 5 years, and 43.7% at 10 years, while freedom from type Ia endoleak was 98.8%, 94.6%, and 85.6%, respectively. Comparing treatment within versus outside the IFU, in terms of survival (HR: 0.94, 95% CI: 0.75–1.16, *p* = 0.53), freedom from reintervention (HR: 0.85, 95% CI: 0.63–1.15, *p* = 0.29) and mortality due to aneurysm-related complication (HR: 0.79, 95% CI: 0.34–1.84, *p* = 0.58) revealed no statistically significant difference. **Conclusions:** The Endurant endograft provides acceptable rates of survival, freedom from secondary intervention, aneurysm-related mortality rates, and freedom from ETIa; however, continuous long-term follow-up surveillance is necessary.

## 1. Introduction

Endovascular aneurysm repair (EVAR) and open surgical repair (OSR) are the two primary approaches for patients requiring elective repair for infrarenal abdominal aortic aneurysm (AAA) [1]. EVAR established its role as the primary treatment choice in AAA management due to its lower early mortality and morbidity rates [1,2]. Technological advancements, with the introduction of newer generation endografts, led to notable improvements in terms of technical and clinical outcomes [3,4,5,6]. Despite this evolution, questions remain regarding the long-term durability of the technique and the associated need for reintervention during follow-up.

One of the most described and studied endovascular grafts for infrarenal abdominal aortic aneurysm repair is the Endurant endograft (Medtronic, Santa Ana, CA, USA) [7,8,9]. While in use for over a decade, the Endurant II-IIs endograft system has demonstrated reliable durability, acceptable sac regression rates, and low aneurysm-related mortality during the mid- and long-term follow-up periods [8,10]. Even when its implantation is performed outside the Instructions for Use (IFU), real-world experience reports encouraging outcomes in patients with hostile aortic neck anatomy and complex aortic pathologies, including endoluminal thrombus and calcification [1,11]. Understanding the evidence gap between IFU and outside-IFU use is important to clarify real-world implications, including effects on procedural success, durability, and complication rates, and it may also help guide patient selection and optimize device performance in clinical practice.

Given the widespread global adoption of the Endurant endograft, this systematic review and meta-analysis aimed, through reconstructing patient-level time-to-event data from individual studies, to evaluate its use in unruptured AAA repair. A subgroup analysis comparing the outcomes of interest between patients treated within versus outside the Endurant’s IFUs was executed.

## 2. Materials and Methods

### 2.1. Study Design and Inclusion/Exclusion Criteria

This systematic review and meta-analysis was performed according to PRISMA (Preferred Reporting Items for Systematic Reviews and Meta-analyses) guidelines [12] and was prospectively registered in the PROSPERO database (registration number: CRD42024621517). PICO (Population/Participants, Intervention, Comparison and Outcome) criteria were applied to define our research question (Table 1).

Studies reporting on the outcomes of interest in patients who underwent EVAR for unruptured AAA with the use of the Endurant endograft were included. Exclusion criteria were defined as follows: (i) studies involving complex or ruptured abdominal aortic aneurysms, (ii) studies using the Endurant stent in combination with other stents, or the treatment of isolated iliac aneurysms, or endosuture aneurysm repair with EndoAnchors, (iii) studies published in a language other than English, (iv) meta-analyses, systematic reviews, editorials, or letters to the editor (Table 2). Complex abdominal aortic aneurysms were defined as aneurysms involving the origins of branching arteries supplying visceral organs. In cases where multiple studies reported on the same population, only the larger study or the one providing the most adequate data in terms of a given Kaplan–Meier curve was analyzed in the present meta-analysis.

### 2.2. Search Strategy

The MEDLINE (via PubMed), Scopus, and Cochrane Library databases (search end-date: 12 November 2024) were searched as shown in Table 2. No time restrictions were applied regarding the time of publication or patients’ enrollment into the cohorts. Title and abstract screening and full text eligibility were assessed by two independent investigators. Any disagreement was resolved after discussion with a third investigator (K.S.). Potentially eligible studies were also hand-searched using the snowball methodology [13]. Two investigators independently collected the Kaplan–Meier curves and extracted the studys’ characteristics into a pre-designed Excel table.

### 2.3. Quality Assessment

The Newcastle–Ottawa Scale was used to assess the risk of bias in the included studies. The first domain is selection, which provides up to four stars, including the representativeness of the exposed cohort, selection of the non-exposed cohort, ascertainment of exposure, and demonstration that the outcome of interest was not present at the start of the study [14]. The second domain is comparability, providing a maximum of two stars. As most of the included studies were not comparative, this domain was considered non-applicable (NA), except for studies that provided data comparing outcomes following the intervention within versus outside the instructions for use (IFU). The final domain is outcome, which evaluates the assessment of outcome, the sufficiency of follow-up length, and the adequacy of follow-up, providing up to three stars. The overall quality and summary of evidence was evaluated using the GRADE assessment [15]. Two independent investigators assessed risk of bias. Any discrepancy was resolved after discussion with a third author (K.S.).

### 2.4. Outcomes

The primary assessed outcomes were survival, freedom from reintervention, freedom from type Ia endoleak (ETIa), and aneurysm-related mortality rates. A subgroup analysis was conducted to compare patients treated within the IFU with those treated outside the IFU in terms of survival, freedom from reintervention, and aneurysm-related mortality. Freedom from type Ia endoleak was defined as a leak in the graft proximal end resulting from an inadequate seal, causing continued pressurization of the aneurysmal sac [1]. Type Ia endoleak in the studies was diagnosed based on either computed tomography angiography (CTA), Duplex ultrasound, or Magnetic Resonance Imaging at 1 month after the EVAR.

### 2.5. Statistical Analysis

#### 2.5.1. Reconstruction of Time to Event Patient Survival Data

The methods described by Wei et al. were used to reconstruct individual patient data (IPD) from the Kaplan–Meier curves of all eligible studies for the long-term survival outcomes [16,17]. Raster and vector images of the Kaplan–Meier survival curves were pre-processed and digitized so that the values reflecting specific time points with their corresponding survival/mortality information could be extracted. Where additional information (e.g., number-at-risk tables or total number of events) were available, they were used to further calibrate the accuracy of the time-to-event values. Departures from monotonicity were detected using isotonic regression and corrected with a pool-adjacent-violators algorithm [16,17]. To confirm the quality of the timing of failure events captured, the consistency with the reported survival or mortality data provided in the original publications was assessed.

#### 2.5.2. One-Stage Survival Meta-Analysis

The Kaplan–Meier method was used to calculate overall survival. The Cox proportional hazards regression model was used to compare within and outside IFU group differences. In this model, every patient within each individual study was assumed to be similarly failure prone to other patients belonging to that study. Survival curves were plotted using the Kaplan–Meier product limit method, which was also used to calculate the Hazard Ratios (HRs) and 95% CIs of each group.

#### 2.5.3. Two-Stage Survival Meta-Analysis

As a sensitivity analysis, we calculated summary HRs and 95% CIs for all individual studies based on the reconstructed IPD and pooled them under the conventional “two-step” meta-analysis. Hazard ratios (HR) and 95% confidence intervals (CI) were calculated using the DerSimonian–Laird random-effects model [18]. A forest plot for each outcome was used to display the pooled estimates graphically. A *p* value < 0.05 was considered significant. Between-study heterogeneity was assessed through Cochran’s Q statistics and by estimating I^2^. I^2^ greater than 50% and *p <* 0.1 indicated significant heterogeneity. Funnel plots were used to assess small-study bias, focusing on publication bias as a potential source. Egger’s test was used when at least 10 studies were included in the analysis of each outcome of interest, and *p* < 0.10 was considered statistically significant, indicating possible small-study bias.

## 3. Results

### 3.1. Study and Patient Characteristics

**Figure 1:**
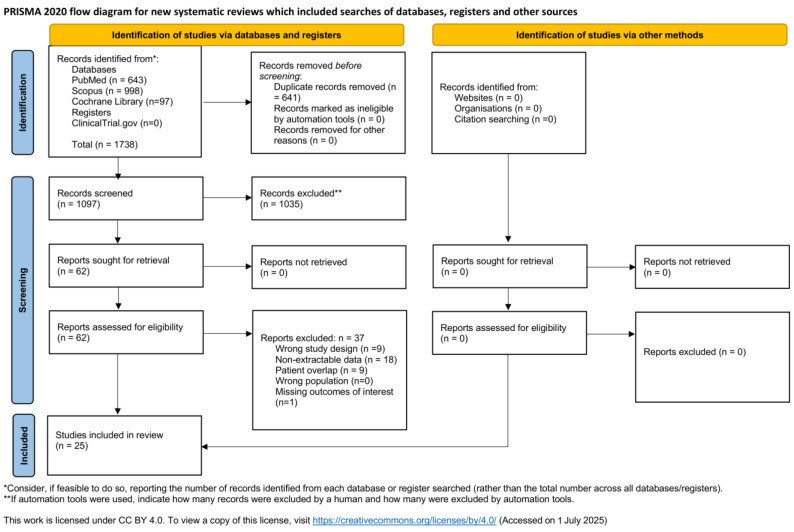
PRISMA flow diagram. Source: Page MJ, et al. MBJ 2021; 372:n71. Doi: 10.1136/bmj.n71. [12].

The literature search yielded 1097 potentially eligible articles after duplicates removed. A total of 9 studies were excluded due to overlapping populations [19,20,21,22,23,24,25,26,27]; 18 were excluded because they did not include a Kaplan–Meier curve; 9 had an unsuitable study design; and 1 study, although it included KM curves, did not provide them for the outcomes of interest. After full-text review, 25 studies met our eligibility criteria as summarized by the PRISMA flowchart (Figure 1) [5,8,10,11,28,29,30,31,32,33,34,35,36,37,38,39,40,41,42,43,44,45,46,47,48]. Even though the studies by Bisdas T. et al. [36] and Troisi N. et al. [37] were referring to the same population, the Kaplan–Meier curve only from the latter was used to extract data for the subgroup analysis regarding the application of the Endurant endograft within or out of the IFU. Concerning the ENGAGE registry [5], the Kaplan–Meier curve of the study by Teijink JAW. et al. comparing the outcomes within versus outside the IFU was reconstructed for the subanalysis [8]. Likewise, regarding the overlapping population between the studies by Oliveira N. et al. [49] and Oliveira Pinto J. et al. [47], the first was excluded from the analysis, as it presented only data concerning clinical success and did not provide data on other main outcomes. Consequently, only the latter study was utilized to reconstruct the Kaplan–Meier curve for the main outcomes. A total of 6199 different patients undergoing EVAR for infrarenal unruptured AAA was identified. The baseline characteristics of the included studies are summarized in Table 3.

### 3.2. Study Quality and Publication Bias Assessment

The studies were assessed for quality using the Newcastle-Ottawa Scale. A total of 6 studies were deemed low quality [11,28,30,31,38,39], 17 studies moderate quality [8,10,32,33,34,35,36,37,40,41,42,43,44,45,46,47,49], and 2 studies high quality [5,29] (Supplemental Table S1). The domain of comparability was evaluated only for studies that provided data comparing treatments within versus outside the IFU. The GRADE assessment and the summary of evidence are presented in Supplemental Table S2 and Supplemental Table S3, respectively. The visual inspection of the funnel plots regarding survival, freedom from secondary reintervention and aneurysm-related mortality suggested potential publication bias, with smaller studies clustering more to the left, indicating lower survival, freedom from reintervention and aneurysm-related mortality rates. This was also supported by the Egger’s test, regarding the survival and the freedom from reintervention outcomes as provided in Supplemental Figure S1–S3.

### 3.3. Time to Event Patient Data and Kaplan–Meier Curves Reconstruction

In total, 19 Kaplan–Meier curves of survival [8,10,11,28,29,30,31,33,34,35,36,38,39,40,42,43,44,45,46], 17 for freedom from secondary reintervention [8,10,30,32,33,34,36,38,40,41,42,43,44,45,46,47,49], 7 reporting on aneurysm-related mortality rates [32,33,35,38,43,44,46], and 4 for freedom from type IA endoleak [8,32,33,42] were processed, digitized, and reconstructed. Regarding the subgroup analysis comparing the outcomes of interest between patients treated within versus outside the IFU, six Kaplan–Meier curves on survival [5,29,35,37,40,45], six on freedom from reintervention [5,37,40,41,43,45], and two reporting on aneurysm-related mortality [5,35] were processed, digitized, and reconstructed. There was insufficient data regarding type Ia endoleak between the two groups, as only one study included a relevant Kaplan–Meier curve [37]. A side-by-side comparison of our reconstructed Kaplan–Meier curves and those found in the original publications is provided in Supplemental Figures S4–S17. Each time point reflects estimates of the outcomes of interest from reconstructed Kaplan–Meier curves and is not derived from a fixed cohort. The at-risk population decreases over time due to censoring, including mortality events and loss to follow-up.

### 3.4. Main Outcomes

#### 3.4.1. Survival and Aneurysm-Related Mortality

**Figure 2:**
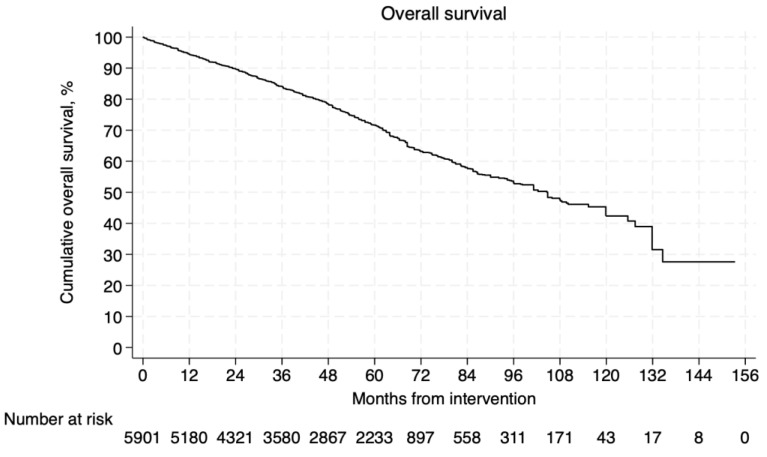
Pooled Kaplan–Meier curve of overall survival.

**Figure 3:**
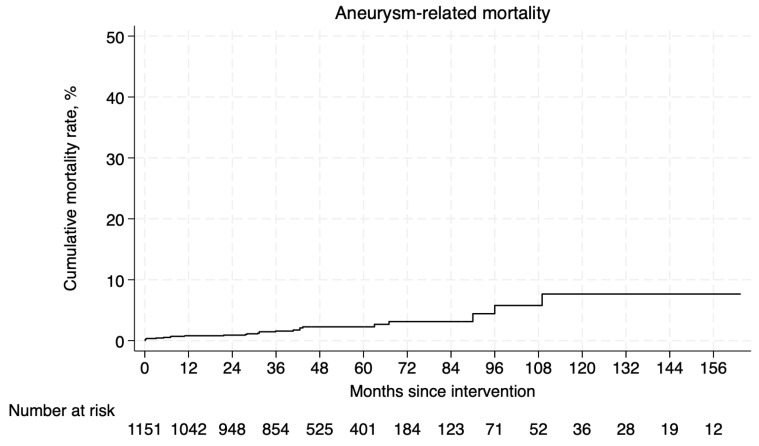
Pooled Kaplan–Meier curve of aneurysm-related mortality.

The overall survival rate was estimated for 5901 patients [8,10,11,28,29,30,31,33,34,35,36,38,39,40,42,43,44,45,46]. The pooled Kaplan–Meier curve is presented in Figure 2. The probability of survival at 1, 5, and 10 years of follow-up was 94.4%, 71.6%, and 42.4%, respectively (mean follow-up 45.8 ± 26.8 months). The overall aneurysm-related mortality was estimated for 1151 patients [32,33,35,38,43,44,46]. The pooled Kaplan–Meier curve of aneurysm-related mortality estimates for the reconstructed IPD is presented in Figure 3. The rates of mortality due to aneurysm-related complications at 1, 5, and 10 years of follow-up were 0.8%, 2.3%, and 7.6%, respectively.

#### 3.4.2. Freedom from Secondary Intervention

**Figure 4:**
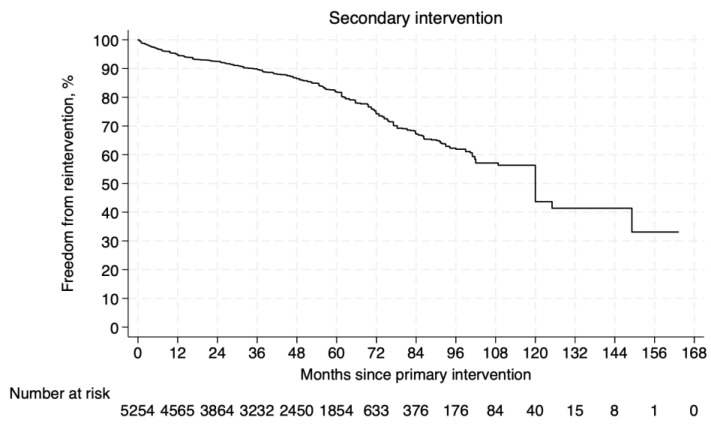
Pooled Kaplan–Meier curve of freedom from secondary intervention.

The pooled Kaplan–Meier curve of reconstructed IPD is presented in Figure 4. The overall freedom from secondary reintervention rates referred to 5254 patients [8,10,30,32,33,34,36,38,40,41,42,43,44,45,46,47,49]. The rates of freedom from reintervention at 1, 5, and 10 years of follow-up were 94.9%, 81.9%, and 43.7%, respectively. An overview of the reasons for intervention is presented in Supplemental Table S4. The most frequent reasons were stenosis or occlusion of the iliac limb (71 cases), Type Ia endoleak (53 cases), and Type II endoleak (49 cases).

#### 3.4.3. Type Ia Endoleak

**Figure 5:**
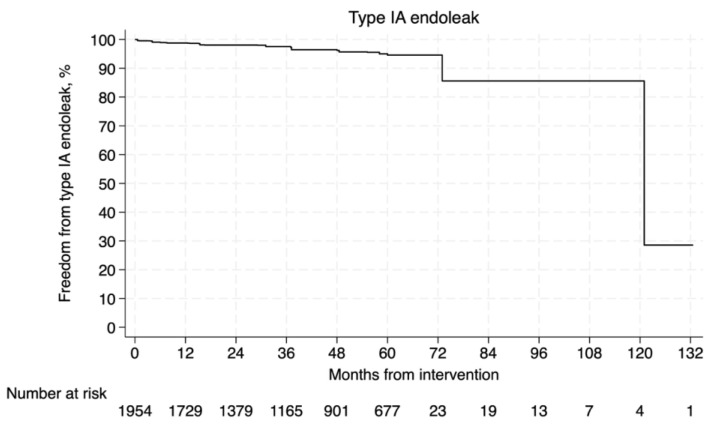
Pooled Kaplan–Meier curve of freedom from type Ia endoleak.

Regarding the freedom from type Ia endoleak, the estimated rate was calculated for 1954 patients [8,32,33,42]. The pooled Kaplan–Meier curve is presented in Figure 5. The rates of freedom from type IA endoleak at 1, 5, and 10 years of follow-up were 98.8%, 94.6%, and 85.6%, respectively.

### 3.5. Subgroup Analysis Within Versus Outside the Endurant’s IFU

#### 3.5.1. Survival

The pooled Kaplan–Meier curve of reconstructed IPD, involving 1448 patients treated within versus 470 treated outside the IFU, is presented in Supplemental Figure S18A [5,29,35,37,40,45]. No statistically significant difference was observed between the two groups (HR: 0.94, 95% CI: 0.75-1.16, *p* = 0.53). The probabilities of survival at 1 and 5 years in the “within the IFU” group were 94.4% and 68.9%, respectively. Concerning the treatment outside the IFU counterpart, the probabilities of survival at 1 and 5 years were 92.7% and 67.3%, respectively. The sensitivity cumulative two-stage meta-analysis based on the pooled HRs of the included studies confirmed the absence of any statistically significant difference between the two groups, with no statistical heterogeneity (HR: 0.88, 95% CI: 0.71–1.08, *p* = 0.21, I^2^ = 0.00%) (Supplemental Figure S18B).

#### 3.5.2. Aneurysm-Related Mortality

Regarding aneurysm-related mortality, data for 1142 patients treated within the IFU versus 299 patients treated outside the IFU were provided [5,35]. The pooled Kaplan–Meier curve of the reconstructed IPD is presented in the Supplemental Figure S19A, revealing no statistically significant difference between the two groups (HR: 0.79, 95% CI: 0.34–1.84, *p* = 0.58). The probabilities of survival at 1 and 5 years of follow-up in the within the IFU group were 99.1% and 97.7%, respectively. Concerning the treatment outside the IFU counterpart, the probabilities of survival at 1 and 5 years of follow-up were 98.7% and 97.4%, respectively. The sensitivity cumulative two-stage meta-analysis based on the pooled HRs following the IPD reconstruction of the included studies confirmed that there was no statistically significant difference between the two arms regarding aneurysm-related mortality, with a low statistical heterogeneity (HR: 1.03, 95% CI: 0.38–2.84, *p* = 0.95, I^2^ = 21.45%) (Supplemental Figure S19B).

#### 3.5.3. Freedom from Secondary Intervention

The data referred to 1378 and 415 patients treated within versus outside the IFU, respectively [5,37,40,41,43,45]. The pooled Kaplan–Meier curve of the reconstructed IPD is presented in Supplemental Figure S20A. The freedom from secondary intervention rates at 1, 5, and 10 years of follow-up in the within the IFU group were 94.2%, 85.2%, and 77.5%, respectively. Concerning the treatment group outside the IFU, the respective rates were 94%, 83.8%, and 53.8%. No statistically significant difference was observed between the two groups (HR: 0.85, 95% CI: 0.63–1.15, *p* = 0.29). After analyzing the pooled HRs of the included studies, the sensitivity cumulative two-stage meta-analysis did not reveal any statistical significance, and the possibility of substantial heterogeneity was low (HR: 0.69, 95% CI: 0.39–1.23, *p* = 0.21, I^2^ = 38.84%) (Supplemental Figure S20B).

### 3.6. Sensitivity Analysis

A sensitivity analysis that excluded the low-quality studies [30,38] demonstrated freedom from reintervention rates of 95.1% (95% CI 94.3–95.8), 84.6% (95% CI 83.1–86.0), and 53.3% (95% CI 43.1–62.4) at 1, 5, and 10 years of follow-up, respectively, based on a total of 3454 patients. (Supplemental Figure S21A). In comparison, the inclusion of all studies yielded corresponding rates of 94.9%, 81.9%, and 43.7%, respectively. Overall survival rates at 1, 5, and 10 years were 94.4% (95% CI 93.5–95.1), 70.7% (95% CI 68.9–72.4), and 44.9% (95% CI 39.2–50.4), respectively (Supplemental Figure S21B), following the exclusion of the low-quality studies [11,30,31,38], compared to 94.4%, 71.6%, and 42.4%, respectively, when all studies were included.

## 4. Discussion

The findings of this systematic review and meta-analysis provide the overall insight into the survival, freedom from reintervention, aneurysm-related mortality, and type Ia endoleak frequency of patients managed with EVAR for infrarenal unruptured AAA using the Endurant endograft. The subgroup analysis comparing the patients receiving the Endurant endograft within versus outside the IFU did not reveal any statistically significant difference in terms of survival; thus patients outside IFU also did well in comparison with patients that were treated according to IFU. Despite the fact that no statistical difference was detected in terms of secondary interventions, patients treated outside the IFUs presented a 20% higher rate at 10 years of follow-up, highlighting the role of baseline anatomy and respect to the IFU in the long-term technical outcomes and durability of EVAR.

The bi-modular Endurant II endograft was used for several years, whereas the newer tri-modular device has been utilized for nearly a decade for the treatment of AAA [50]. The newer Endurant IIs system is a three-piece design based on the previous endograft, featuring a differently designed bifurcated component and a shorter main body [50]. In our meta-analysis, most included studies did not specify which type of endograft was used, except for the study by Kemmling S. et al., which directly compared the two models with comparable outcomes of mortality, endoleaks, reintervention, and technical success [49]. Troisi N. et al. analyzed 79 patients treated for abdominal aortic aneurysm using only the Endurant IIs device, with a follow-up period of one year [51]. They reported a rate of freedom from type I endoleaks of 96.6% and a rate of freedom from limb occlusion of 100%. In comparison, the corresponding one-year rates reported by Kemmling S. et al. from a group of 34 patients were 98.1% and 94.6%, respectively. Regarding freedom from reintervention, the rates were 100% in the Troisi cohort and 85.3% in the Kemmling S. et al. study. Although these results suggest a generally comparable clinical performance between the two groups, differences in patients’ characteristics and follow-up design may account for the observed differences in the reported rates. Despite the continuously growing use of the Endurant II endograft, dedicated data to the newer design are limited or unextractable from the available literature [49,51].

The current meta-analysis demonstrated survival probabilities of 71.6% and 42.4% at 5 and 10 years of follow-up, respectively, confirming previous analyses with similar survival rates at the same time points [29,30]. However, the aneurysm-related mortality after EVAR remained as low as 7.6% at 10 years follow-up. This might suggest that the overall long-term mortality is influenced by patients’ comorbidities and age-related factors, and EVAR remains an effective approach in reducing aneurysm-related mortality in the long term follow-up. Similarly low rates of aneurysm-related mortality in patients treated with EVAR are presented in the DREAM, EVAR, OVER, and ACE randomized controlled trials, at 5.7%, 1%, 2.7%, and 4%, respectively [52,53,54,55]. Although these findings might highlight the durability and the efficacy of EVAR in preventing aneurysm-related mortality, the decline in overall survival underscores the need for further comprehensive approach to address non-aneurysmal causes of mortality. However, it is important to highlight that the above studies were conducted over a decade ago using earlier-generation endografts, with different patient selection strategies and postoperative surveillance protocols [52,53,54,55]. In contrast, 23 of the 25 studies included in our analysis were conducted after 2014, reflecting the use of newer-generation devices and contemporary clinical practices. These differences may have influenced the pooled estimates and may partially explain the observed variations in long-term survival. Furthermore, rigorous follow-up protocols with strict data collection are necessary to ensure the accuracy of the assessment of long term outcomes, as loss to follow-up may contribute to an underestimation of both aneurysm-related and all-cause mortality.

Various periprocedural factors, including hostile neck anatomy and complex anatomical features, have been associated with challenges in achieving adequate sealing at the proximal and distal ends of the endograft [1,23]. These difficulties can lead to clinical failure and ultimately necessitate reinterventions. This meta-analysis suggests a freedom from reintervention rate of 81.9% at 5 years, while roughly half of patients with follow-up at 10 years after EVAR remained free from reintervention, which appears to align with the available literature reported in the randomized trials comparing EVAR to OSR [56]. Especially regarding the extended follow-up of 10 years, previous large cohort data of patients managed with various endografts showed a lower rate of freedom from reintervention, which was estimated at 33% compared to 56.3% in the current meta-analysis [57]. This discrepancy may be attributed to differences in patients’ baseline and endograft characteristics. The importance of long-term follow-up is highlighted by this study, as late complications such as endoleaks, graft migration, and infection can occur well after the initial intervention. Unfortunately, there are insufficient data regarding the type and complexity of reintervention, which are clinically significant and could influence the long-term outcomes and follow-up strategies. Type Ia endoleaks, proximal neck degeneration, and graft migration are associated with a higher risk of aneurysm-related complications, such as aneurysm sac enlargement and rupture, necessitating more intensive imaging surveillance and timely intervention [58,59]. Meanwhile, distal limb occlusion or type Ib endoleaks may necessitate distal reintervention after targeted clinical follow-up and imaging [60,61]. The distinction between major interventions, such as open surgical conversion or aortic relining, and minor reinterventions, such as percutaneous thrombectomy, the coil embolization of type II endoleaks, or isolated limb stenting interventions, is also clinically relevant, as major interventions are typically associated with greater morbidity and may indicate a worse prognosis. A complex reintervention is often necessary after a failed EVAR with a type Ia endoleak, most commonly involving F/BEVAR rather than open surgical repair, which is associated with higher perioperative mortality and morbidity rates [1]. Although reinterventions might be perceived as negative outcomes in a study, they often indicate effective post-EVAR surveillance with the early detection of complications. Since most studies did not differentiate between major and minor reinterventions, future research should focus on this distinction, as the clinical significance of each for patient outcomes differs substantially.

Type Ia endoleaks are associated with inadequate sealing at the neck, caused by angulation, or neck length, and the presence of thrombus or calcification at the sealing zone [62]. Rates of freedom from type Ia endoleaks at 5 and 10 years were 94.6% and 85.6%, respectively, in the conducted meta-analysis. All four studies included in the reconstruction reported rates of over 80% at 5 years of follow-up. As type Ia endoleaks can remain asymptomatic, it is important to perform regular imaging and surveillance to monitor the integrity of the endograft and prevent long-term complications. The ability to promptly detect and treat type Ia endoleaks is essential for preserving the overall efficacy of EVAR.

A study examining the suitability of various endografts in real-world data, with strict adherence to each device’s IFU, concluded that the Medtronic Endurant had a highly suitable rate of 80.7% [63]. Despite this, minor deviations between the patient’s anatomy and the IFU often do not alter the physician’s decision when the endograft appears to be the best overall option for the patient’s anatomical profile. However, as evaluated by Schanzer et al., data from 10,228 patients revealed that only 42% of their anatomies aligned with the respective device’s IFUs [64]. This misalignment was associated with higher rates of aneurysm sac enlargement, raising concerns about the subsequent risk of aneurysm rupture. Unfortunately, none of the studies included in our meta-analysis provided data on aneurysmal sac behavior after EVAR. This limitation significantly reduces our ability to assess long-term device durability and the risk of late rupture, both of which are clinically important outcomes. While most existing studies report similarly comparable outcomes in terms of survival, aneurysm-related mortality, and freedom from reintervention during short term follow-up periods of 1–3 years [5,45], some studies have found a significantly higher rate of type Ia endoleaks during long-term follow-up beyond 5 years [37,65]. Despite the modest observed visual advantage in the reconstructed Kaplan–Meier curves favoring treatment within the IFU over outside the IFU in terms of survival, freedom from secondary intervention, and freedom from aneurysm-related mortality, these findings were not statistically significant. Although there was no statistically significant difference in long-term freedom from reintervention, it is notable that patients treated within the IFU had a 20% higher rate of freedom from reintervention at 10 years (77.5%) compared to those treated outside the IFU (53.8%). While this difference of 20% did not reach statistical significance, likely due to limited sample size and wide confidence intervals, the clinical implications of this finding remain important. The lack of statistical significance may be attributed to differences in sample size, heterogeneity in follow-up duration, and methodology, as well as the absence of detailed procedural and anatomical data across studies. Individualized risk assessment and intensified post-EVAR surveillance are recommended in cases where treatment outside the IFU is deemed necessary in order to promptly detect potential complications. It should be noted that the sample sizes for the outside IFU groups were relatively small (470 vs. 1448 for survival and 299 vs. 1142 for aneurysm-related mortality). This limitation reduces the statistical power of the subgroup analyses, and the non-significant findings should therefore be interpreted with caution, as they may be attributable to insufficient sample size rather than true equivalence. To arrive at more definitive and safe conclusions regarding the outcomes of treatment within versus outside the IFU, larger-scale studies with larger patient populations are required.

### Limitations

There are several limitations in this study that should be acknowledged. First, there was the inherent limitation of a study-level meta-analysis that cannot adjust for residual confounding factors. Similarly, the study does not present patient-level data on prognostic factors or perioperative variables that could significantly affect clinical outcomes. Instead, it reports only aggregated results, which limits our ability to perform detailed analyses. Specifically, the absence of these data prevents the control of critical adjustments such as patient comorbidities, anatomical variations, or procedural specifics that may have influenced the observed outcomes. This limitation introduces the possibility of confounding, particularly in subgroup analyses, where underlying baseline differences such as age, comorbidities, or aneurysm morphology might have contributed to the variability in results. Furthermore, without access to individual patient data, it is not possible to conduct multivariable analyses to adjust for these potential confounders. Since our analysis included only studies that provided Kaplan–Meier curves, there is a potential for publication bias, as studies without these data were excluded. Although the pooled estimates for freedom from reintervention, survival, and aneurysm-related mortality may be affected by small-study effects, as indicated by funnel plot asymmetry, the overall direction and interpretation of our findings remain unchanged. Furthermore, there is a distinction between the use of bi-modular and tri-modular Endurant endografts and their possible effects on the outcomes of interest. Unfortunately, most of the included studies did not distinguish between the bi-modular and tri-modular versions of the Endurant device. Consequently, our pooled analysis combines outcomes from both device types. This mixing may weaken the precision of our estimates and limit the ability to draw conclusions about the performance of the newer tri-modular device. Therefore, the results should be interpreted with caution, as they may not fully reflect the outcomes associated with each device type individually. Moreover, all the procedures in the included studies were performed in different centers by different operators. The majority of studies did not consistently report certain adverse events, including rupture rates and distinctions between major and minor reinterventions. Additionally, due to the nature of our analysis, relying on published data rather than direct access to patients’ records, the study is limited by the lack of data regarding the type of reintervention and the specific reasons behind violations of the IFU. A secondary intervention for relining the distal limb differs from a proximal repair for an endoleak, and violations of the IFU in the proximal neck length or angulation could affect the outcomes differently compared to violations involving the distal limb. Similarly, the variability in the post-procedural protocols among the included studies may introduce bias in the assessment of long-term outcomes. The lack of standardized post-EVAR surveillance protocols across theincluded studies introduces potential bias in long-term estimates. Differences in imaging surveillance, clinical assessments, and follow-up schemes could lead to the underreporting of late aneurysm-related complications, as some outcomes may have been missed due to inconsistent or limited long-term assessment. These facts might affect the generalizability and the external validity of our findings.

## 5. Conclusions

The Endurant endograft seems to provide a reliable solution for EVAR, with acceptable rates of survival and low aneurysm-related mortality during long term follow-up. The freedom of ETIa rate remained >80% at 10 years of follow-up in the total cohort of patients. Patients treated within the IFU achieved a freedom from reintervention rate above 77%, highlighting a lower long-term reintervention risk, whereas robust follow-up is especially important for those treated outside the IFU.

## Figures and Tables

**Table 1:** PICO criteria.

PICO Element	Description
Population	Adult patients undergoing EVAR for infrarenal unruptured AAA, including those with symptomatic status, if mentioned
Intervention	EVAR using the Endurant endograft
Comparison	Non-applicable
Outcomes	Survival, freedom from reintervention, aneurysm-related mortality, and freedom from type IA endoleak rates

Abbreviations: EVAR: Endovascular aneurysm repair, AAA: abdominal aortic aneurysm.

**Table 2:** Search strategy.

Search Criteria	Description
Inclusion criteria	Studies reporting on the outcomes of interest concerning patients undergoing EVAR for unruptured AAA with the Endurant endograft
Exclusion criteria	(i) Studies involving complex or ruptured AAAs (ii) Studies using the Endurant stent with other stents, treating isolated iliac aneurysms, or using EndoAnchors (iii) Non-English language publications (iv) Meta-analyses, systematic reviews, editorials, and letters to the editor
Study selection	Inclusion of only studies with the largest sample size or best quality data (including Kaplan–Meier curves)
Algorithm	PUBMED: ((endostent*) OR (endurant*) OR (“endovascular graft”)) AND (((“AAA”) OR (“abdominal aortic aneurysm”) OR (“aortic aneurysm”)) OR (abdominal aorta aneurysm [MeSH Terms]) OR (aneurysm, abdominal aortic [MeSH Terms])). Scopus: TITLE-ABS-KEY ((“Endurant stent” OR “Endurant graft” OR “endovascular graft”) AND (“abdominal aortic aneurysm” OR “infrarenal abdominal aortic aneurysm”)) Cochrane (Title Abstract Keyword): ((endostent) OR (endurant*) OR (“endovascular graft”)) AND ((“AAA”) OR (“abdominal aortic aneurysm”) OR (“aortic aneurysm”))
Last Search	12 November 2024
Sources	Medline, Scopus, Cohrane database, Clinicaltrial.gov
Subgroup analysis	Comparison of outcomes between patients treated within versus outside the Endurant’s IFUs

Abbreviations: EVAR: endovascular aneurysm repair, AAA: abdominal aortic aneurysm, IFU: instructions for use.

**Table 3:** Study characteristics.

Author	Publication Year	Center	Country	Design	Study Period	Number	IFU Versus Outside IFU
Rouwet EV et al. [28]	2011	10 EU centers	10 EU centers	Prospective	November 2007–August 2008	80	No
Benveniste GL et al. [29]	2020	Ashford Vascular Clinic, Adelaide, South Australia	Australia	Prospective	August 2008–March 2019	180	Yes
ΜO Falster et al. [30]	2023	Australasian Vascular Audit, New South Wales	Australia	Prospective	January 2010–June 2019	1713	No
Kvinlaug KE et al. [31]	2012	McGill University Health Centre in Montreal, Quebec, the London Health Sciences Centre, University of Western Ontario, the Sudbury Regional Hospital in Sudbury, Ontario	Canada	Prospective	September 2008–January 2010	107	No
van Basten Batenburg M et al. [32]	2022	ΕAGLE, Multicenter Europe	Europe	Prospective	February 2012–September 2017	150	No
Becquemin JP et al. [33]	2021	20 French centers	France	Prospective	March 2012–April 2013	180	No
Omran S et al. [34]	2023	Universitätsmedizin Berlin, Berlin	Germany	Prospective	January 2013–May 2021	165	No
Özdemir-van Brunschot DMD et al. [35]	2024	Augusta Hospital and Catholic Hospital Group Düsseldorf, Düsseldorf	Germany	Retrospective	January 2012–December 2020	178	Yes
Bisdas T et al. [36]	2014	Department of Vascular Surgery, St. Franziskus Hospital and University Clinic of Muenster, Muenster	Germany	Prospective	November 2007–December 2010	273	No
Troisi N et al. [37]	2014	St. Franziskus Hospital, Munster, the Center for Vascular and Endovascular Surgery, University Hospital, Munster, Department of Vascular Surgery, University of Florence	Germany, Italy	Prospective	November 2007–March 2010	173	Yes
Deery SE et al. [38]	2019	24 USA centers, ENGAGE post-approval	USA	Prospective	June 2011–August 2012	178	No
ENGAGE [5]	2021	ENGAGE registry, 79 centers worldwide	International	Prospective	March 2009–April 2011	1263	Yes
Teijink JAW et al. [8]	2019	ENGAGE registry, 79 centers worldwide	International	Prospective	March 2009–April 2011	1263	No
Sekimoto Y et al. [39]	2023	10 Japanese hospitals	Japan	Retrospective	January 2012–July 2019	332	No
Vedani SM et al. [40]	2022	Department of Vascular Surgery, CHUVLausanne	Switzerland	Retrospective	2014–2018	60	Yes
Μatsagkas M et al. [41]	2015	Ιoannina University Hospital	Greece	Prospective	September 2008–December 2012	57	Yes
Georgiadis S et al. [43]	2023	University General Hospital of Alexandroupolis	Greece	Prospective	January 2009–December 2016	184	Yes
Singh MJ et al. [44]	2016	U.S. Endurant Stent Graft System regulatory trial	USA	Prospective	June 2008–April 2009	150	No
Pecoraro F et al. [45]	2016	Vascular Surgery Unit, AOUP “P. Giaccone”, University of Palermo	Ιtaly	Retrospective	December 2012–March 2015	64	Yes
Setacci F et al. [11]	2014	Vascular and Endovascular Surgery Unit, University of Siena	Ιtaly	Retrospective	January 2010–December 2010	137	No
t Mannetje YW et al. [46]	2017	Department of Vascular Surgery, Catharina Hospital, Eindhoven	The Netherlands	Retrospective	January 2005– December 2010	131	No
Oliveira Pinto J et al. [47]	2019	Erasmus University Medical Centre, Rotterdam	Τhe Netherlands	Prospective	July 2004–December 2011	156	No
Salemans PB et al. [10]	2021	Department Of Vascular Surgery, Isala, Zwolle	The Netherlands	Retrospective	February 2009–December 2012	165	No
Kemmling S et al. [48]	2022	University Hospital of Schleswig Holstein, Lübeck	Germany	Prospective	2013–2018	100	No
Spanos K et al. [42]	2024	Department of Vascular Surgery, Larissa University Hospital	Greece	Retrospective	2008–2024	361	No

Abbreviations: IFU: Instructions for use.

## Data Availability

Not applicable.

## Supplementary Materials

The following supporting information can be downloaded at: https://www.mdpi.com/article/10.3390/jcm14186453/s1, Figure S1: Funnel plot regarding survival; Figure S2: Funnel plot regarding freedom from reintervention; Figure S3: Funnel plot regarding freedom from aneurysm related mortality; Figure S4: Original and regenerated Kaplan-Meier (KM) curves of Mannetje Y.W. et al., Becquemin J.P. et al, Benveniste G.L. et al., Bisdas T. et al. and Deery S.E. et al. regarding overall survival probability; Figure S5: Original and regenerated Kaplan-Meier (KM) curves of Falster M.O. et al., ENGAGE registry, Georgiadis S.G. Et al., Kvinlaug K.E. et al. and Kvinlaug K.E. et al. regarding overall survival probability; Figure S6: Original and regenerated Kaplan-Meier (KM) curves of Özdemir-vanBrunschot D.M.D. et al., Pecoraro F. et al., Rouwet E.V. et al., Salemans P.B. et al. and Sekimoto Y. et al. regarding overall survival probability; Figure S7: Original and regenerated Kaplan-Meier (KM) curves of Setacci F. et al., Singh M.J. et al, Spanos K. et al. and Vedani S.M. et al. regarding overall survival probability; Figure S8: Original and regenerated Kaplan-Meier (KM) curves of Mannetje Y.W. et al., Becquemin J.P. et al., Bisdas T. et al., ENGAGE registry and Deery S.E. et al. regarding overall freedom from reintervention; Figure S9: Original and regenerated Kaplan-Meier (KM) curves of Falster M.O. et al, Georgiadis S.G. et al., Kemmling S. et al., Matsagkas et al. and Oliveira Pinto J. et al. regarding overall freedom from reintervention; Figure S10: Original and regenerated Kaplan-Meier (KM) curves of Pecoraro F. et al., Salemans P.B. et al., Singh M.J. et al., Spanos K. et al. and van Basten Batenburg M. et al. regarding overall freedom from reintervention; Figure S11: Original and regenerated Kaplan-Meier (KM) curves of Vedani S.M. et al. and Omran S. et al. regarding overall freedom from reintervention; Figure S12: Original and regenerated Kaplan-Meier (KM) curves of Mannetje Y.W. et al., Becquemin J.P. et al., Deery S.E. et al., Georgiadis S.G. et al. and Özdemir-van Brunschot D.M.D et al. regarding overall aneurysm related mortality; Figure S13: Original and regenerated Kaplan-Meier (KM) curves of Singh M.J. et al, and van Basten Batenburg M. et al. regarding overall aneurysm related mortality; Figure S14: Original and regenerated Kaplan-Meier (KM) curves of Becquemin J.P. et al., ENGAGE Registry, Spanos K. et al. and van Basten Batenburg M. et al. regarding overall Type IA Endoleak; Figure S15: Original and regenerate Kaplan-Meier (KM) curves of within IFU versus outside IFU regarding Survival; Figure S16: Original and regenerated Kaplan-Meier (KM) curves of within IFU versus outside IFU regarding freedom from reintervention; Figure S17: Original and regenerated Kaplan-Meier (KM) curves of within IFU versus outside IFU regarding aneurysm related mortality; Figure S18: Kaplan Meier curve of reconstructed data and forest plot regarding overall survival comparing patients treated IFU versus outside IFU; Figure S19: Kaplan Meier curve of reconstructed data and forest plot regarding aneurysm related mortality comparing patients treated IFU versus outside IFU; Figure S20: Kaplan Meier curve of reconstructed data and forest plot regarding freedom from secondary intervention comparing patients treated IFU versus outside IFU; Figure S21: Kaplan Meier curve of reconstructed data regarding freedom from secondary intervention and overall survival after excluding the low-quality studies. Table S1: The Newcastle-Ottawa Scale was used to assess the risk of bias in the included studies.; Table S2: GRADE assessment of the including studies regarding the quality of evidence.; Table S3: Summary of evidence; Table S4: Data regarding reasons for reintervention.
