# Supplementary material for: Endurant Stents in Abdominal Aortic Aneurysm Repair: A Systematic Review and Meta-Analysis
Source: J Clin Med. 2025 Sep 12;14(18):6453. doi: 10.3390/jcm14186453 (PMC12470529; doi:10.3390/jcm14186453)

Kaplan–Meier (KM) curves of overall aneurysm related mortality

Supplemental Figure S12

A. Original and regenerated KM of Mannetje Y.W. et al. [46]

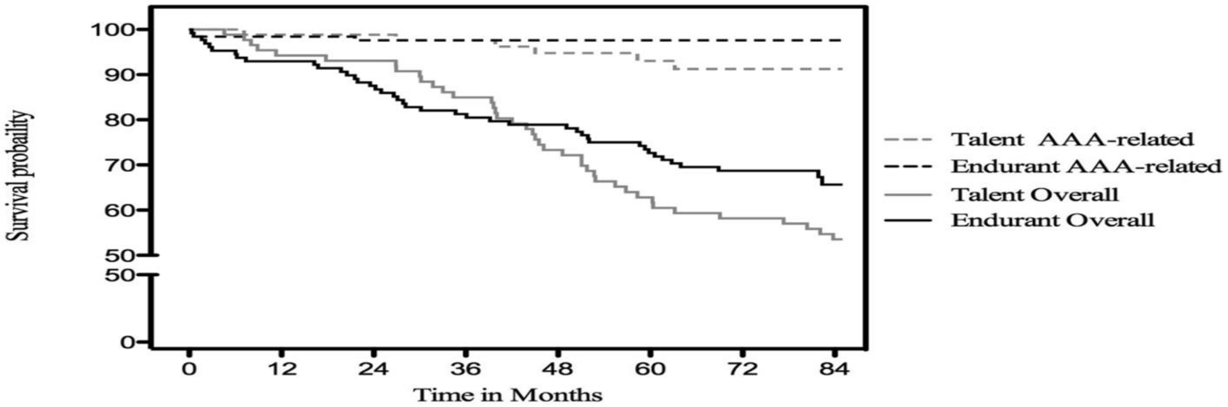

	Day 0	30-days	1-year	5-years	7-years
Talent overall<br>Patients at risk (SE)	90	88<br>(.000)	83<br>(.025)	54<br>(.052)	46<br>(.054)
Endurant overall<br>Patients at risk (SE)	131	126<br>(.011)	119<br>(.023)	93<br>(.039)	32<br>(.045)
Talent AAA<br>Patients at risk (SE)	90	88<br>(.000)	83<br>(.011)	54<br>(.030)	46<br>(.035)
Endurant AAA<br>Patients at risk (SE)	131	126<br>(.022)	119<br>(.011)	93<br>(.014)	32<br>(.014)

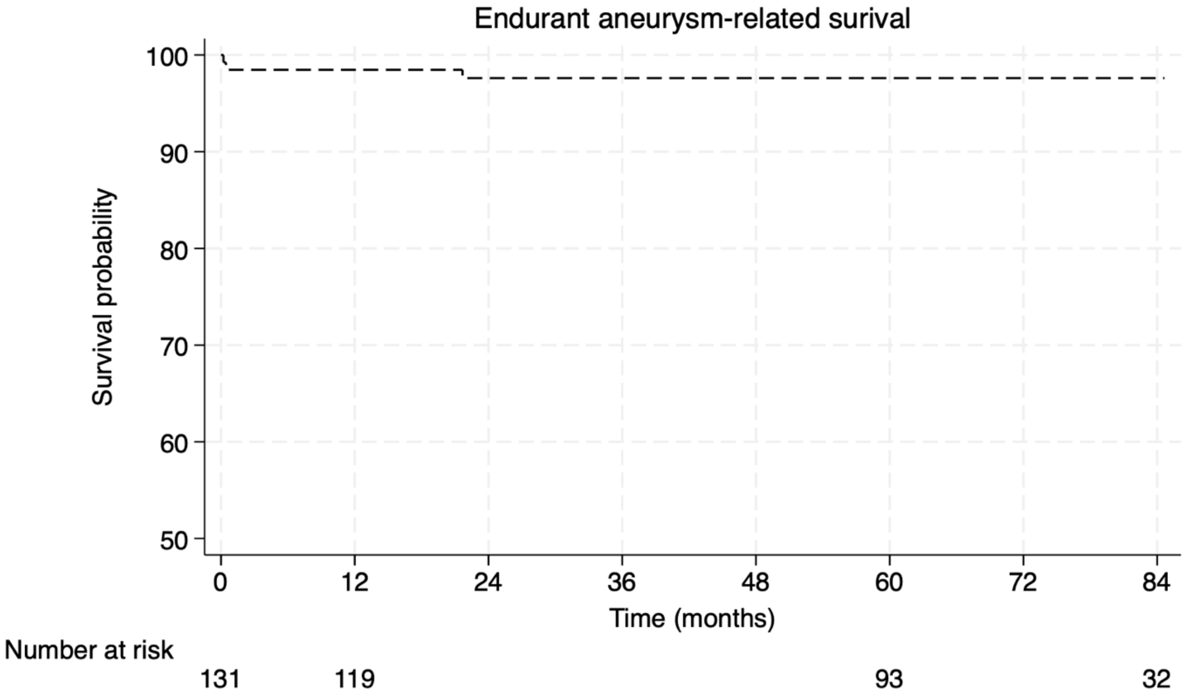

B. Original and regenerated KM of Becquemin J.P. et al. [33]

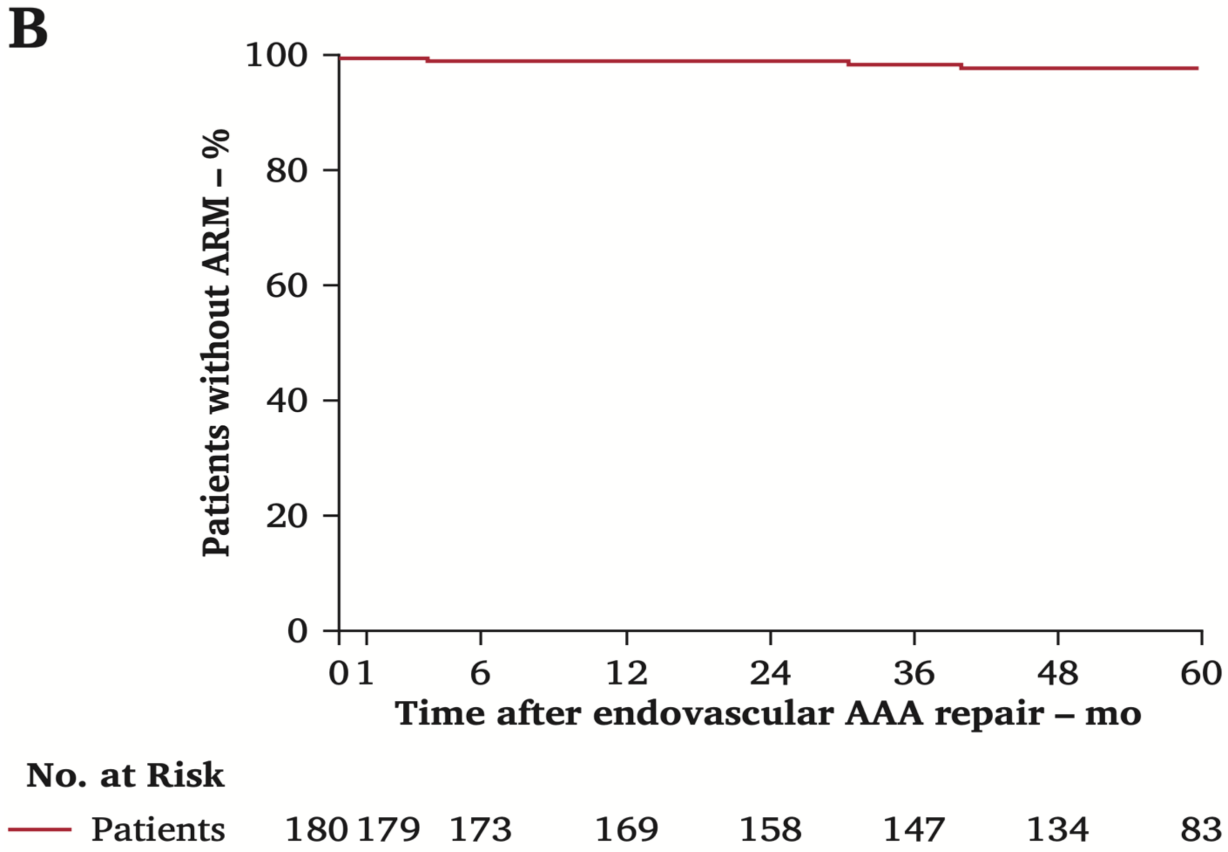

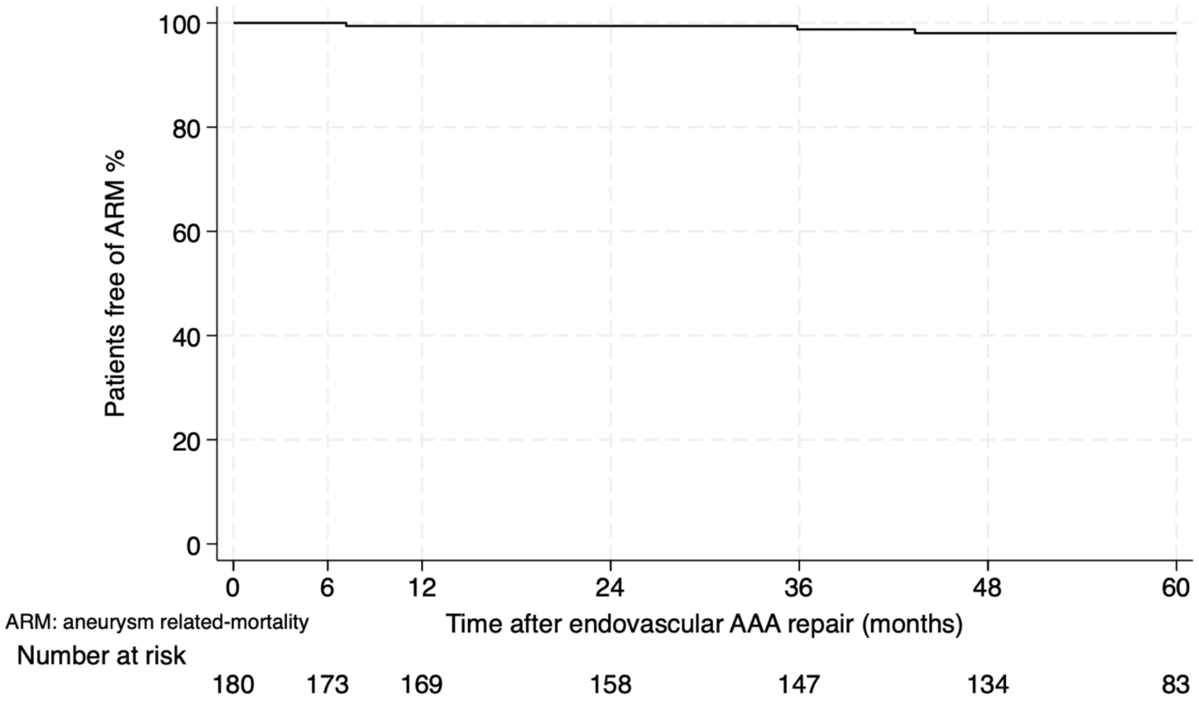

C. Original and regenerated KM of Deery S.E. et al. [38]

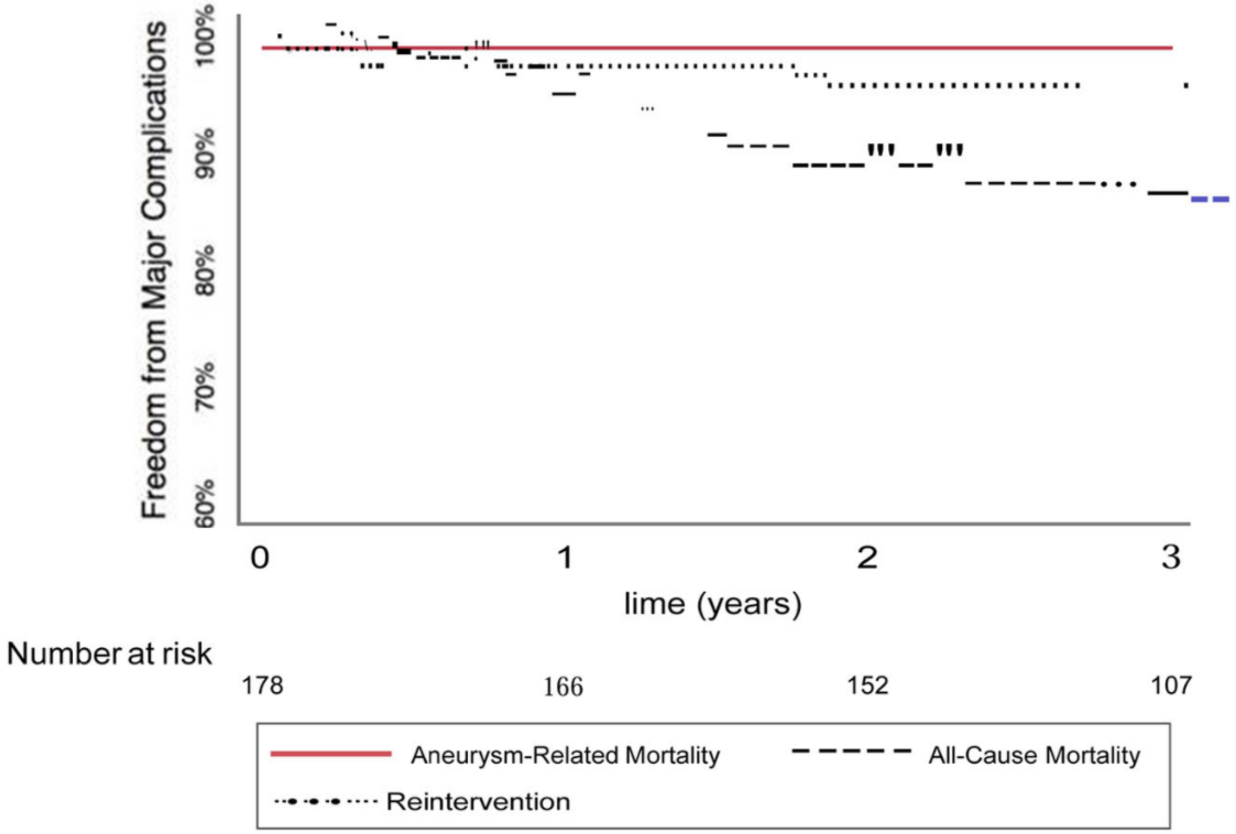

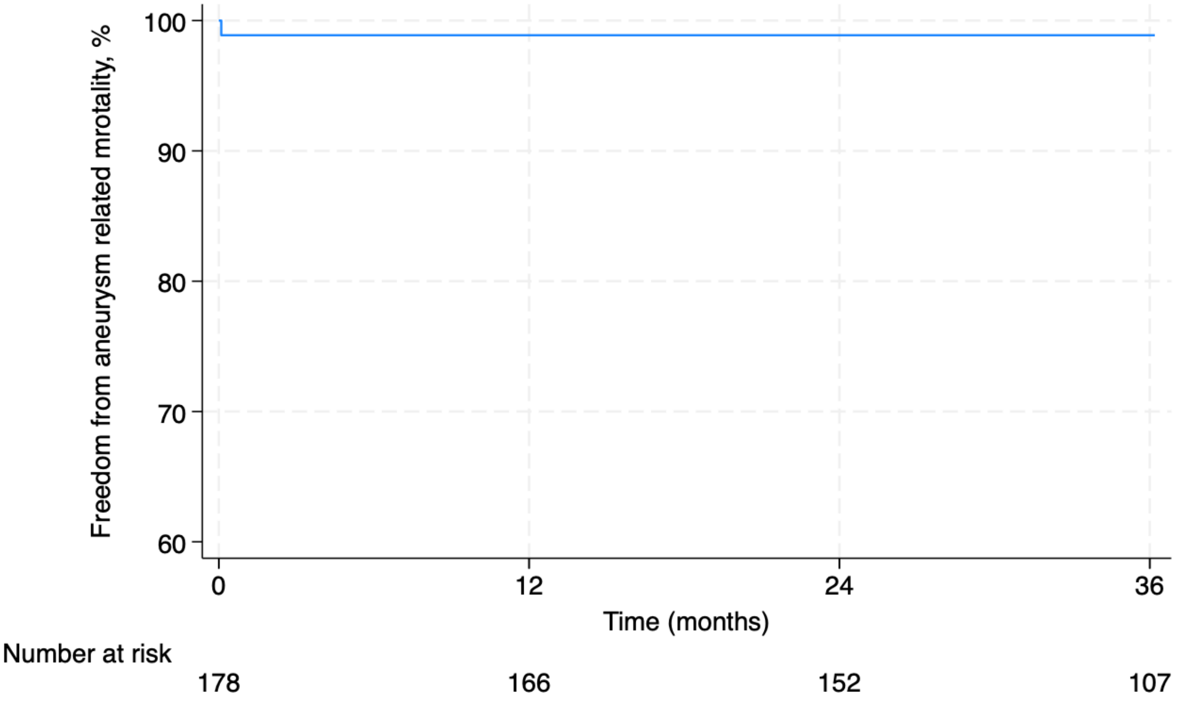

D. Original and regenerated KM of Georgiadis S.G. et al. [43]

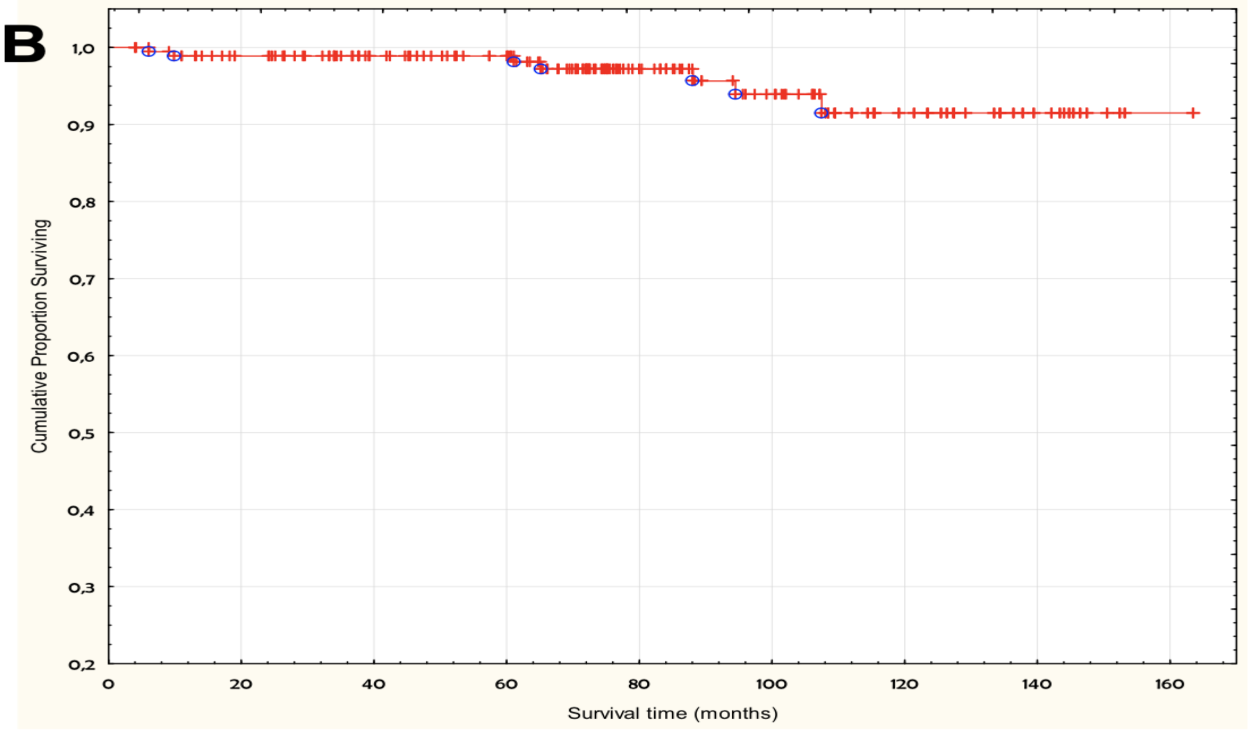

Time (months)	24	36	48	60	72	84	96	120
N at risk	168	150	135	127	99	69	53	26

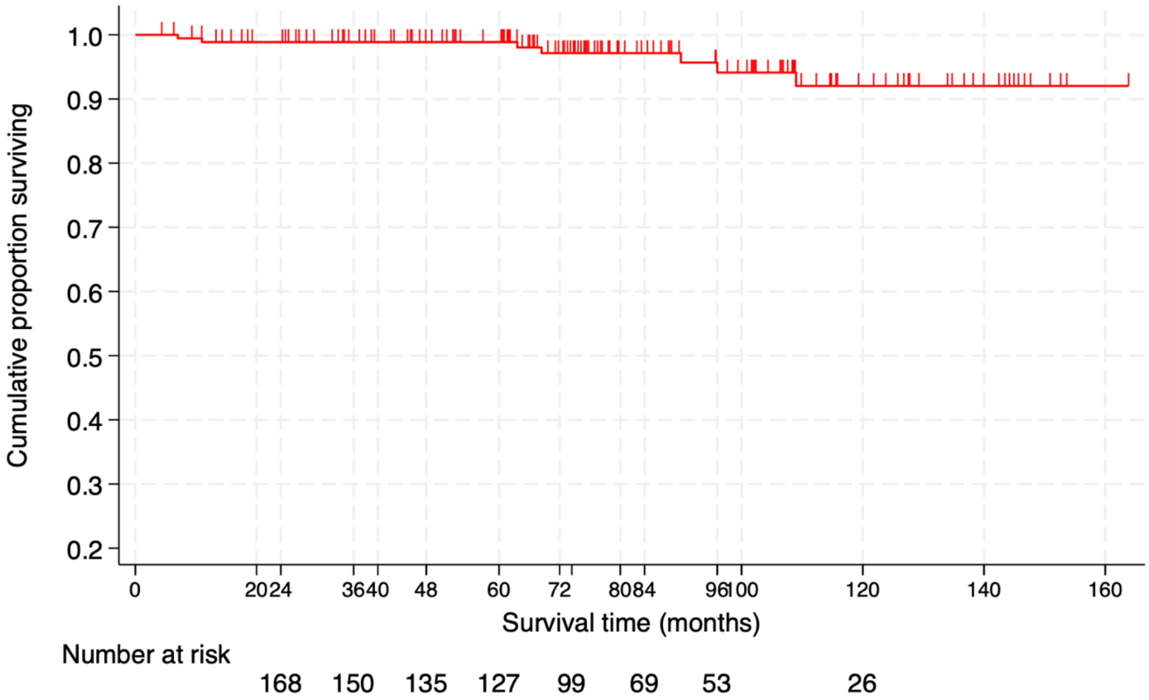

E. Regenerated KM of Özdemir-van Brunschot D.M.D. et al. [35]
(The extracted data were used to reconstruct a single final KM)

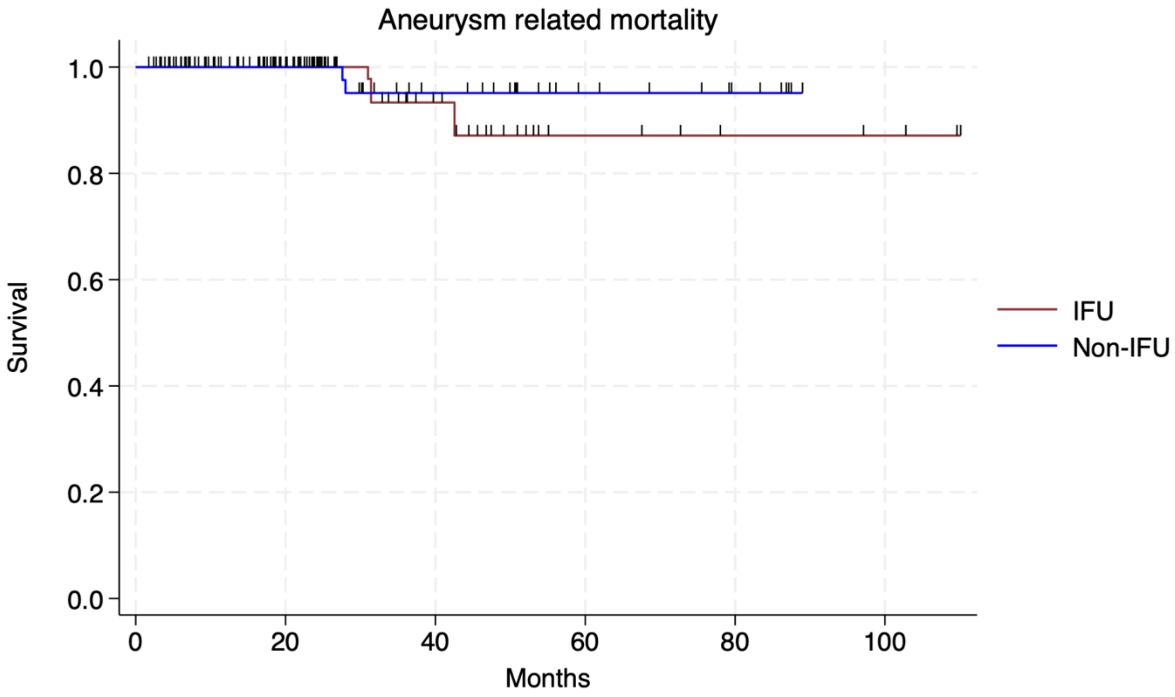

Supplementary Figure S13.

A. Original and regenerated KM of Singh M.J. et al. [44]

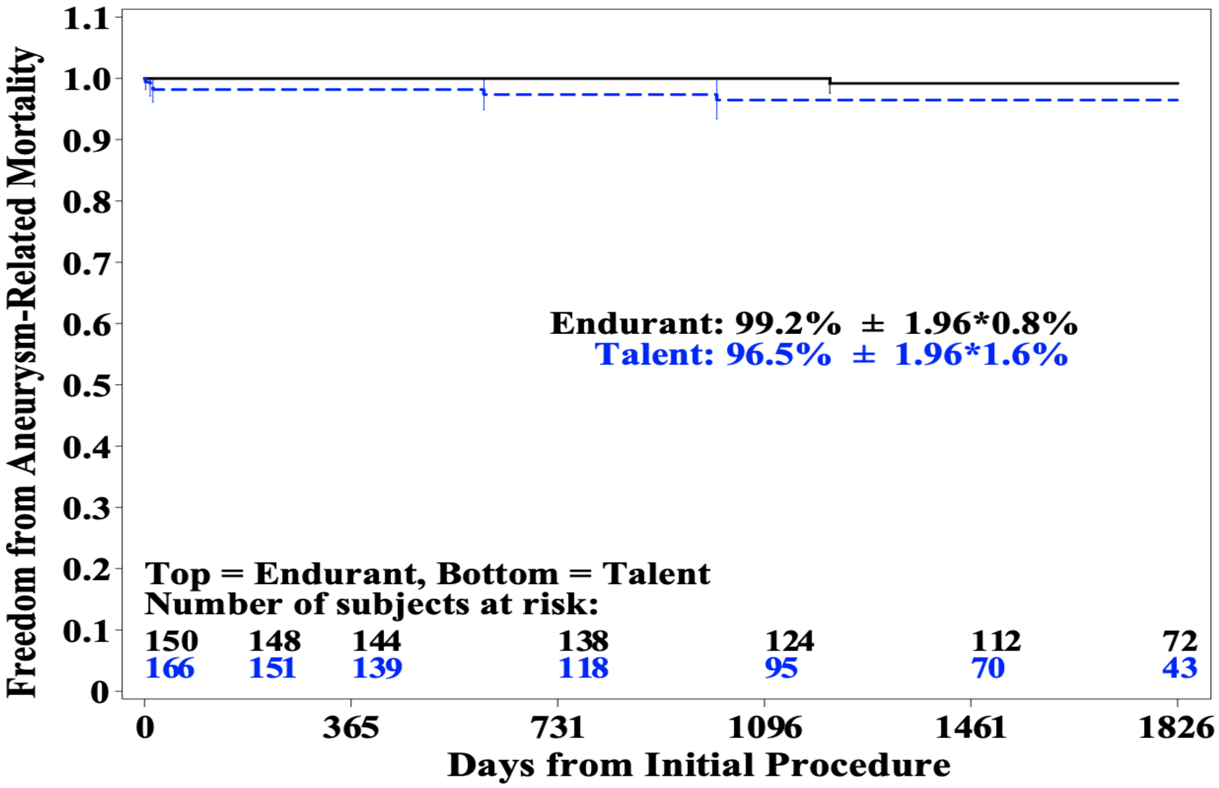

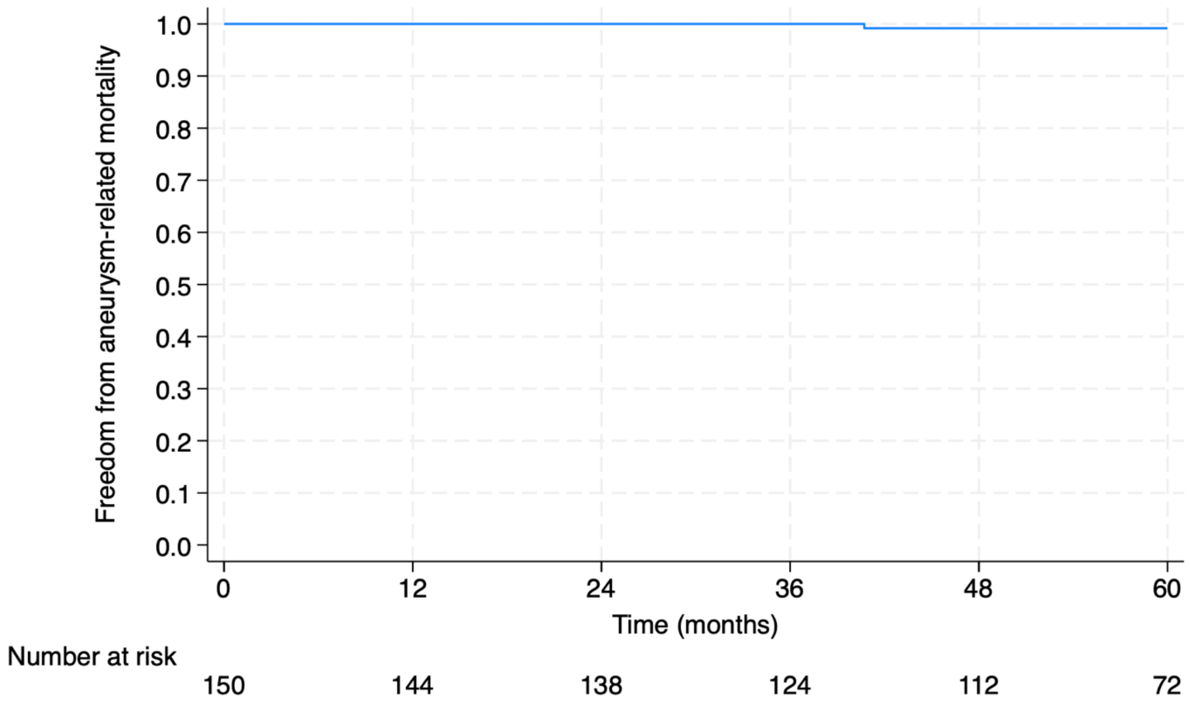

B. Original and regenerated KM of van Basten Batenburg M. et al. [32]

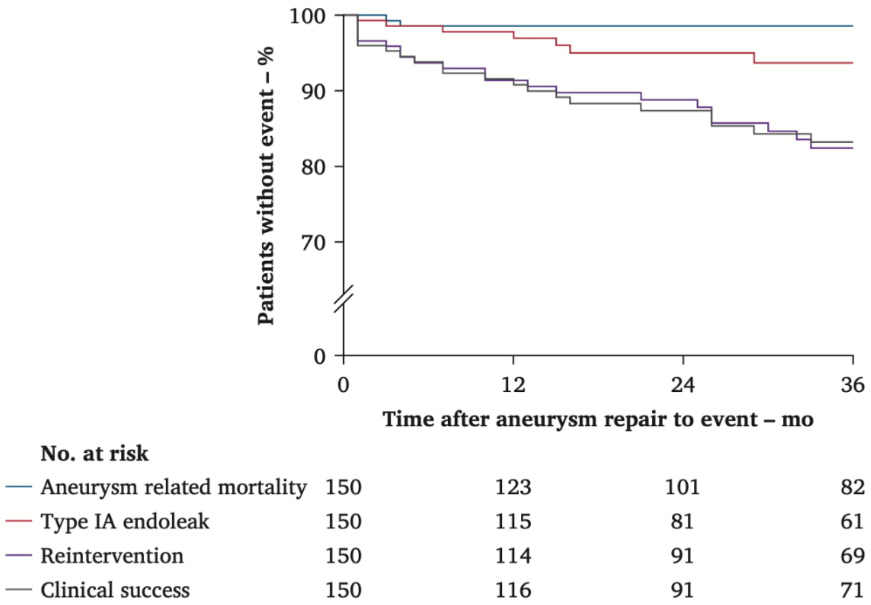

Figure 3. Cumulative Kaplan–Meier estimate of freedom from aneurysm related mortality, type IA endoleak and re-intervention, as well as clinical success in 150 patients with abdominal aortic aneurysms with challenging neck anatomy treated with endovascular aneurysm repair outside instructions for use. analysis. Note the adapted scale for the *y* axis.

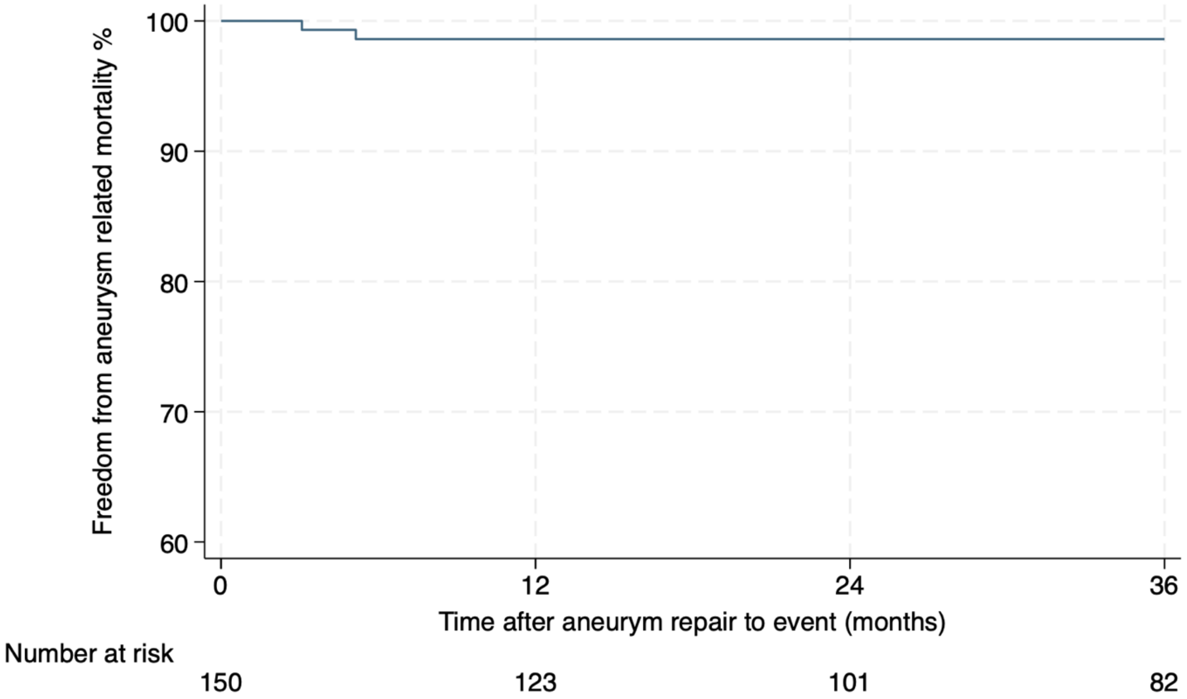

Kaplan–Meier (KM) curves of overall Type IA Endoleak

Supplemental Figure S14.
A. Original and regenerated KM of Becquemin J.P. et al. [33]

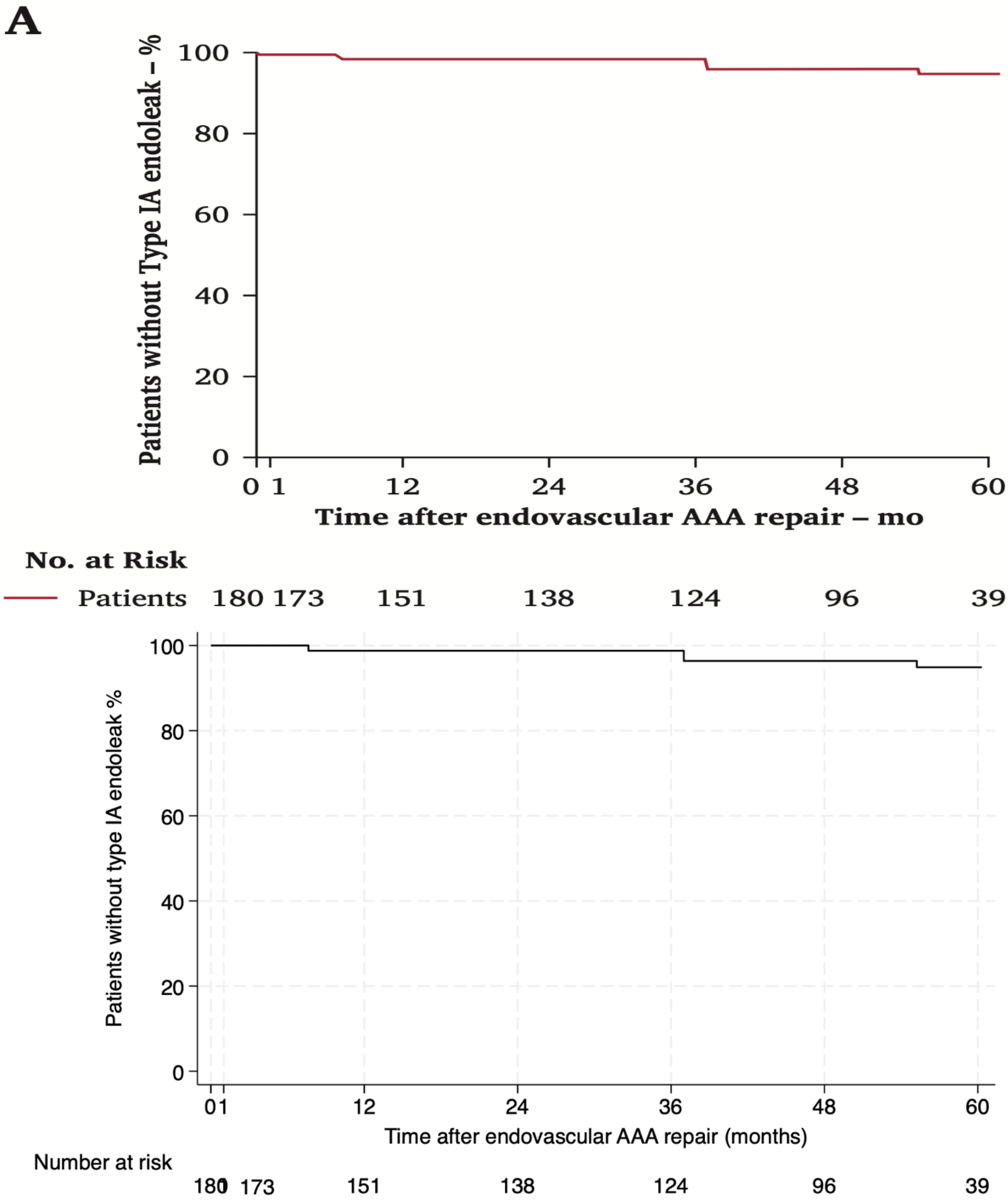

B. Original and regenerated KM of ENGAGE Registry. [5]

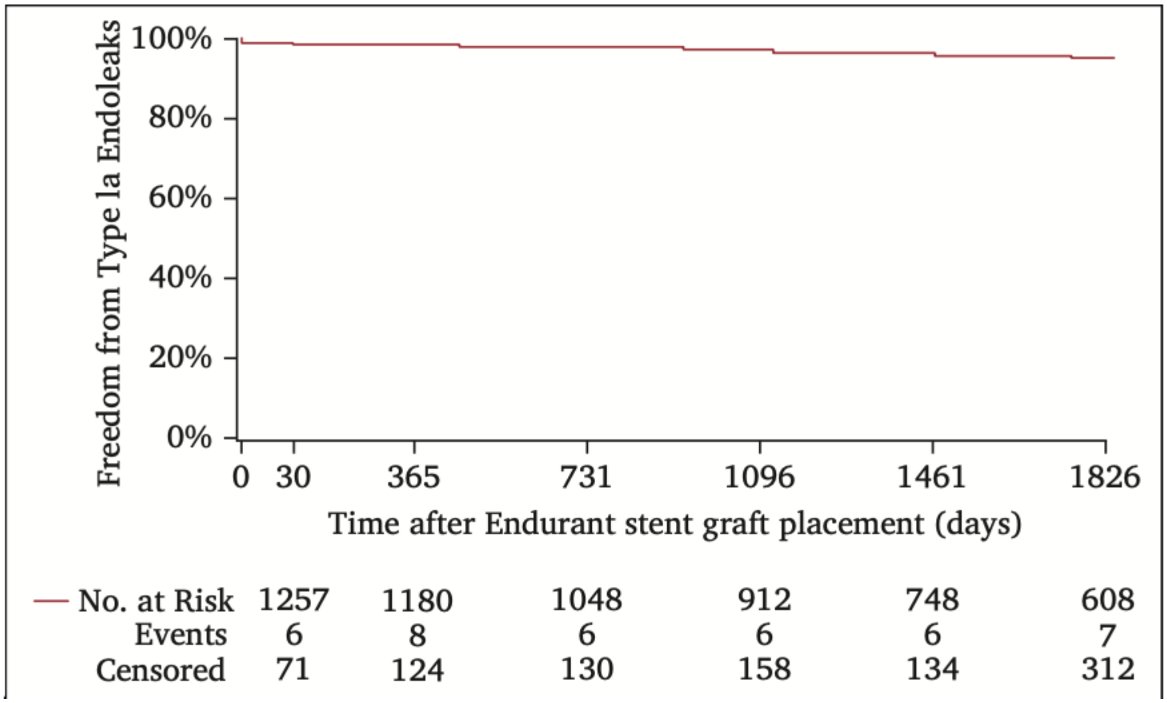

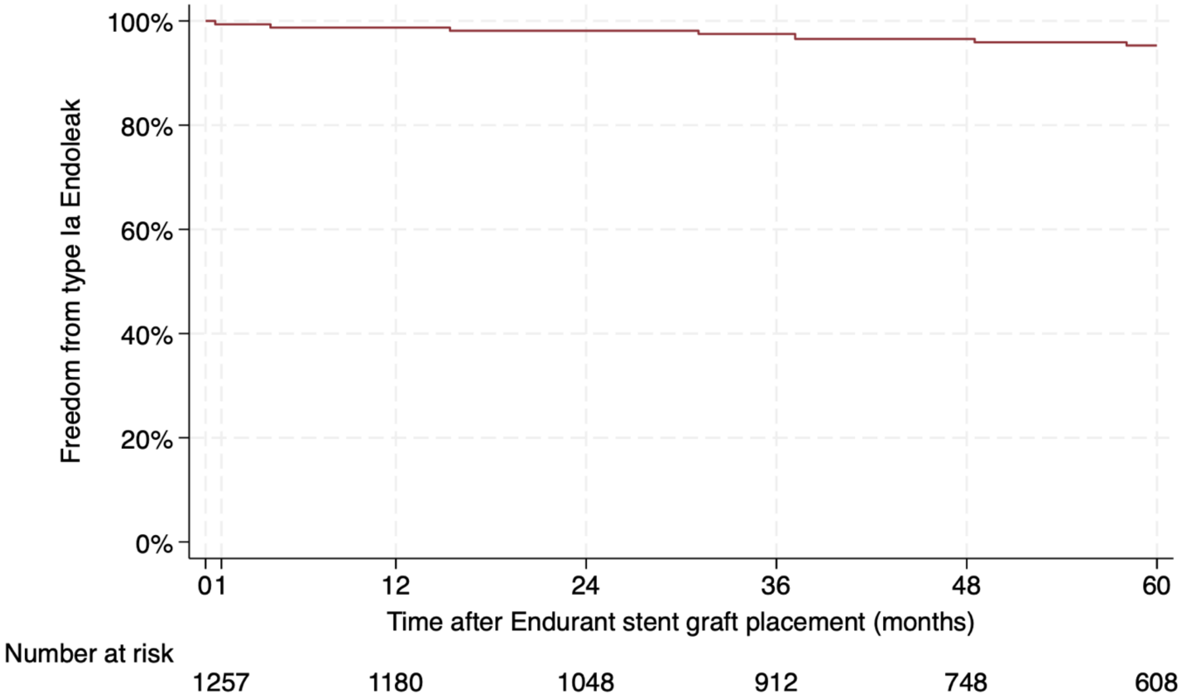

C. Original and regenerated KM of Spanos K. et al. [42]

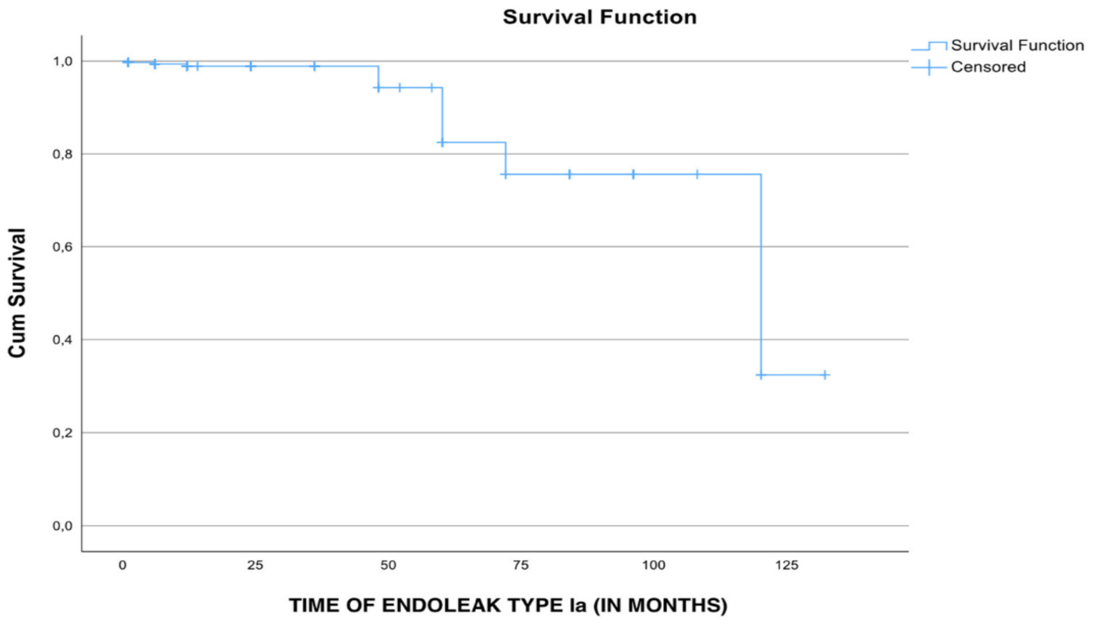

Months	12	24	36	48	60	72	84	96	108
Patients at risk	269	77	59	42	31	23	18	12	7
Events	3	3	3	5	9	11	11	11	11
Percentages	99%	-	-	94.3%	83%	76%	-	-	-
SE	0.7%	-	-	3.2%	6.2%	7.3%	-	-	-

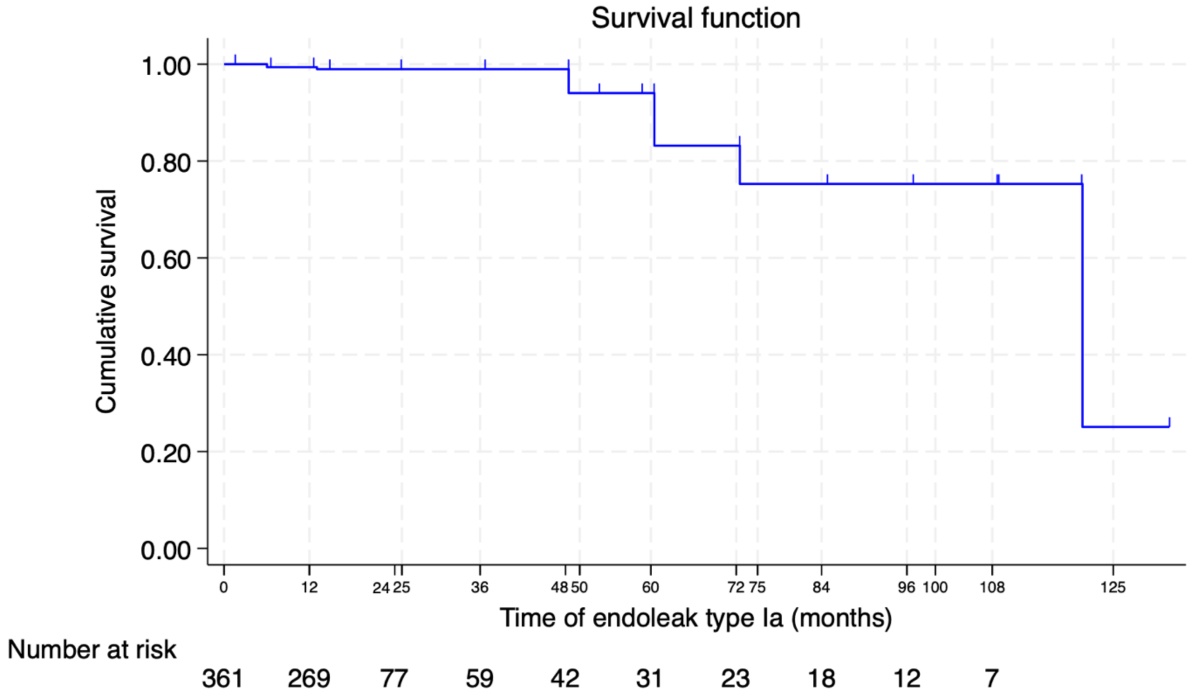

D. Original and regenerated KM of van Basten Batenburg M. et al. [32]

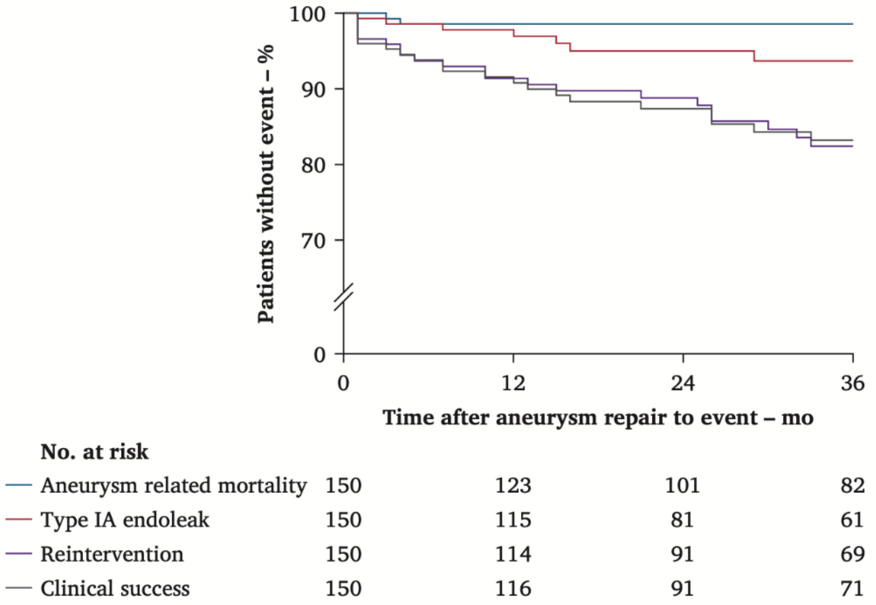

Figure 3. Cumulative Kaplan–Meier estimate of freedom from aneurysm related mortality, type IA endoleak and re-intervention, as well as clinical success in 150 patients with abdominal aortic aneurysms with challenging neck anatomy treated with endovascular aneurysm repair outside instructions for use. analysis. Note the adapted scale for the *y* axis.

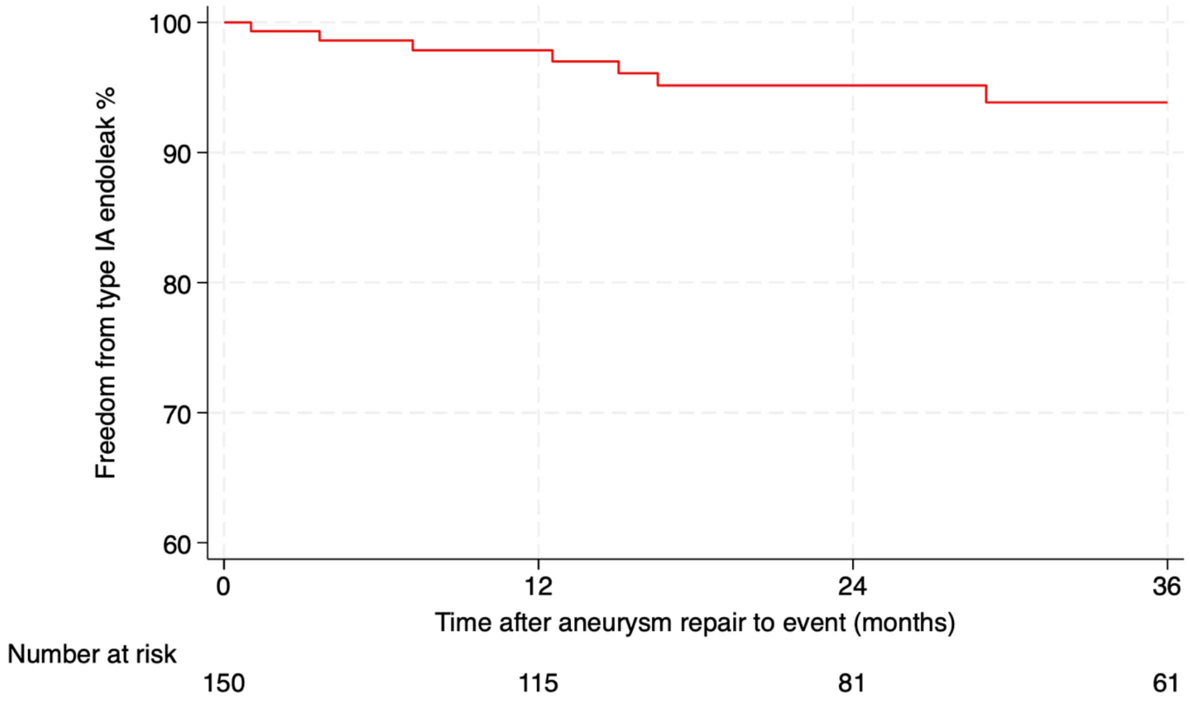

Supplement: Supplementary file 1 [file jcm-14-06453-s001.zip › Supplemental Figures S12-S14.pdf]
