# Supplementary material for: Endurant Stents in Abdominal Aortic Aneurysm Repair: A Systematic Review and Meta-Analysis
Source: J Clin Med. 2025 Sep 12;14(18):6453. doi: 10.3390/jcm14186453 (PMC12470529; doi:10.3390/jcm14186453)

Kaplan–Meier (KM) curves of within IFU versus outside IFU regarding Survival
Supplemental Figure S15

A. Regenerated KM of Benveniste G.L. et al. [29]

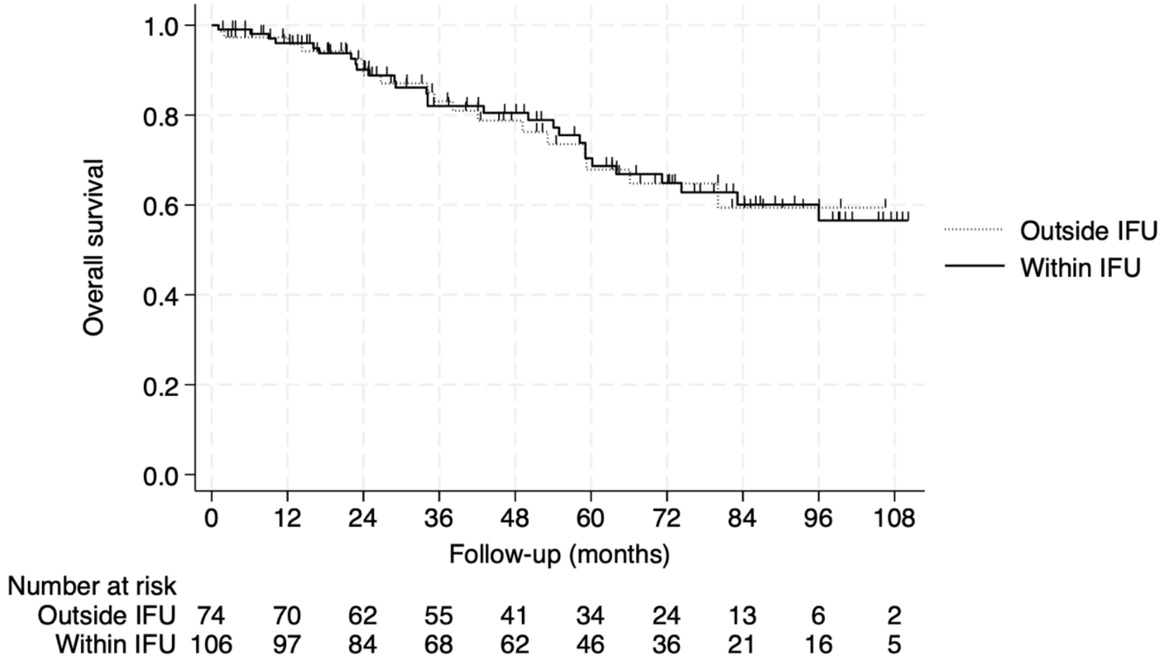

B. Regenerated KM of Özdemir-van Brunschot D.M.D. et al. [35]

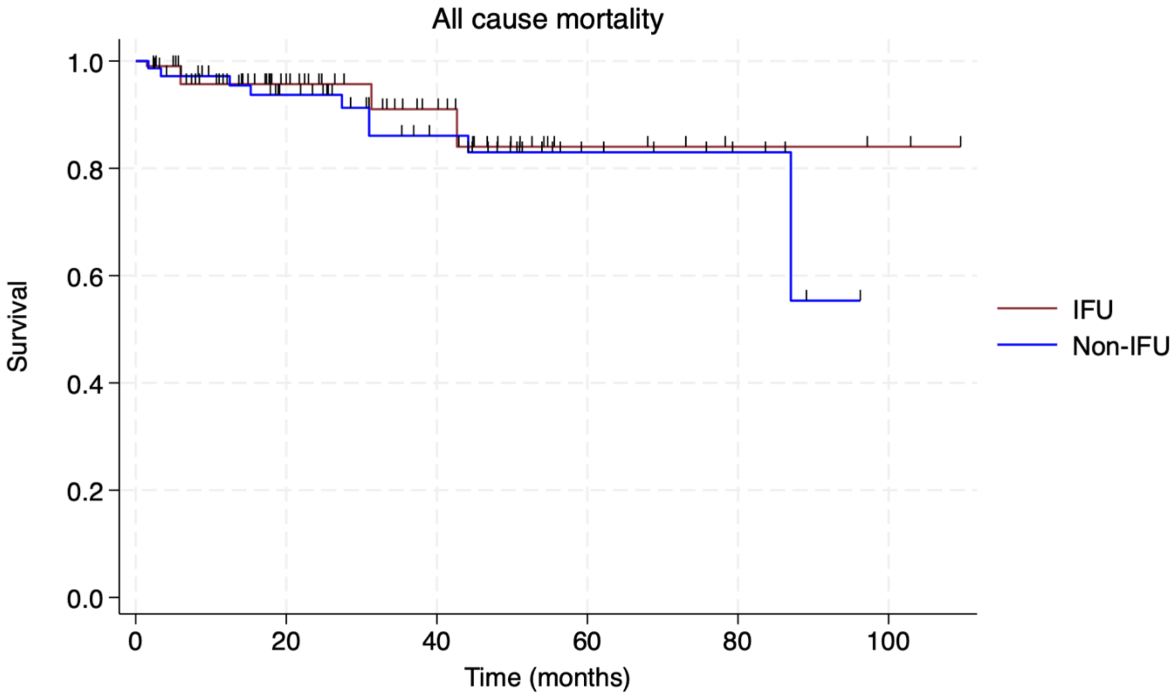

C. Original and regenerated KM of ENGAGE Registry. [5]

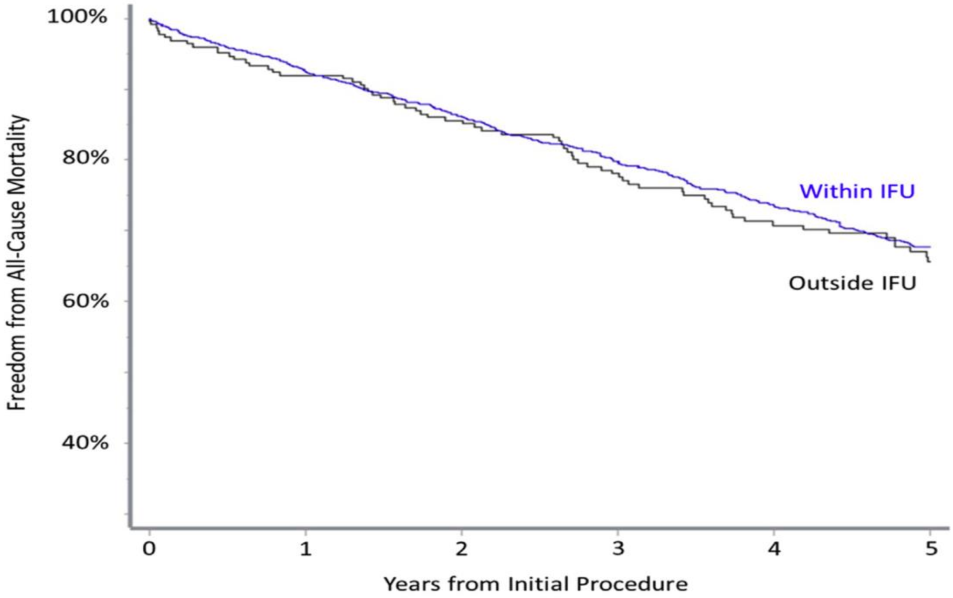

	Outside IFU						Within IFU					
Time Interval	0-30 days	31-365 days	366-731 days	732-1096 days	1097-1461 days	1462-1826 days	0-30 days	31-365 days	366-731 days	732-1096 days	1097-1461 days	1462-1826 days
No. at Risk[1]	225	219	203	177	153	131	1038	1024	947	821	740	648
No. of Events	5	13	14	15	14	8	11	66	66	61	56	46
No. Censored[2]	1	3	12	9	8	35	3	11	60	20	36	227
KM Estimate[3]	0.978	0.919	0.856	0.781	0.708	0.656	0.989	0.925	0.86	0.795	0.734	0.677
Peto SE	0.010	0.018	0.024	0.030	0.033	0.041	0.003	0.008	0.011	0.013	0.015	0.018

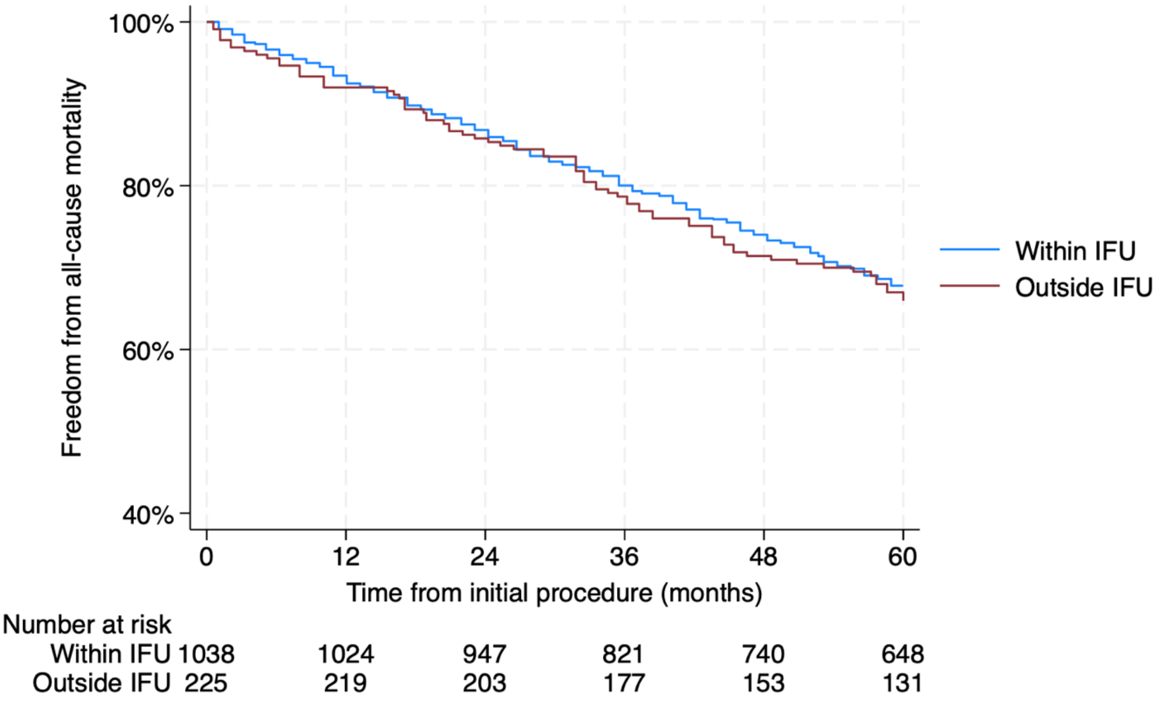

D. Original and regenerated KM of Vedani S.M. et al. [40]

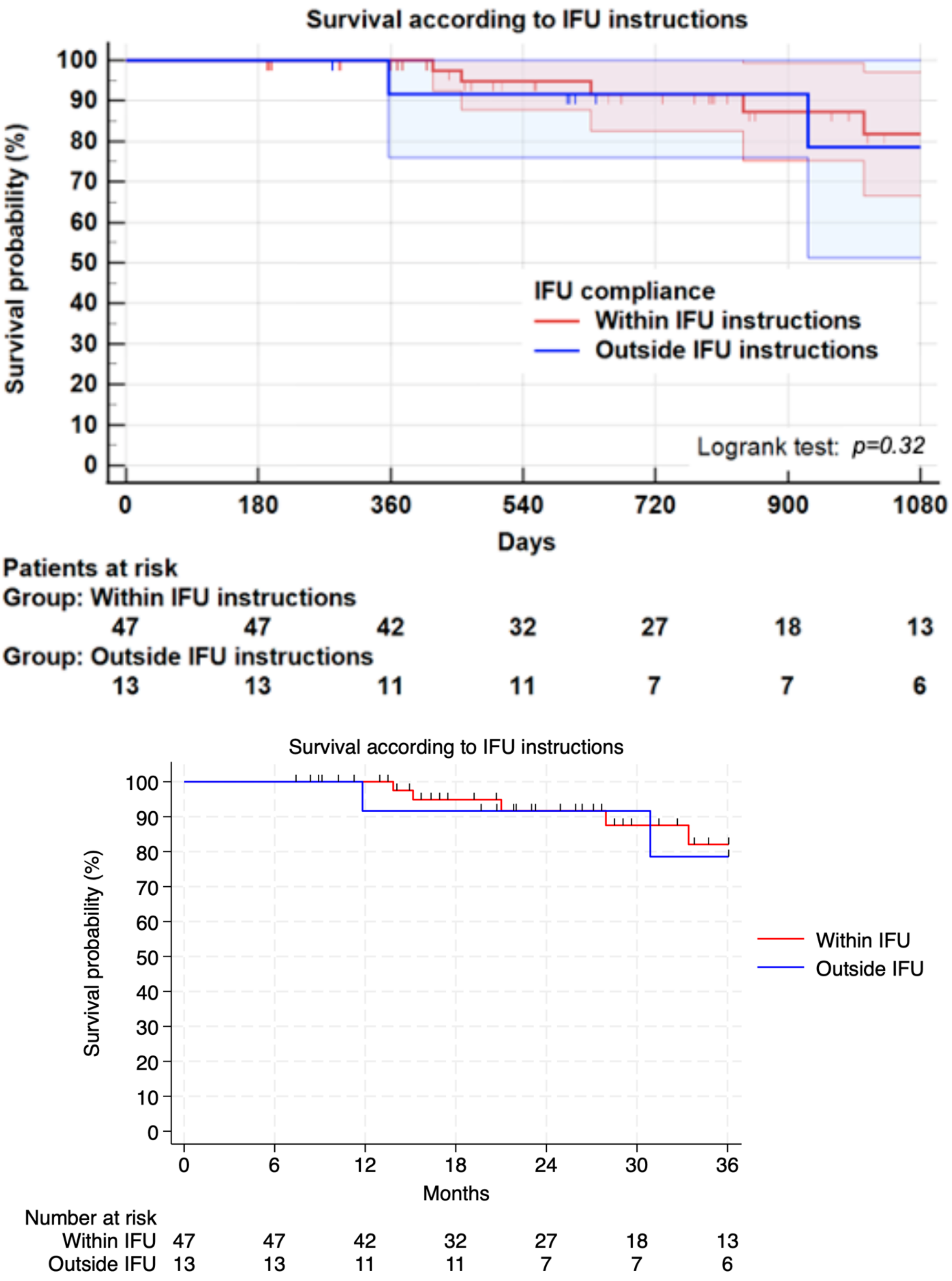

E. Original and regenerated KM of Troisi N. et al. [37]

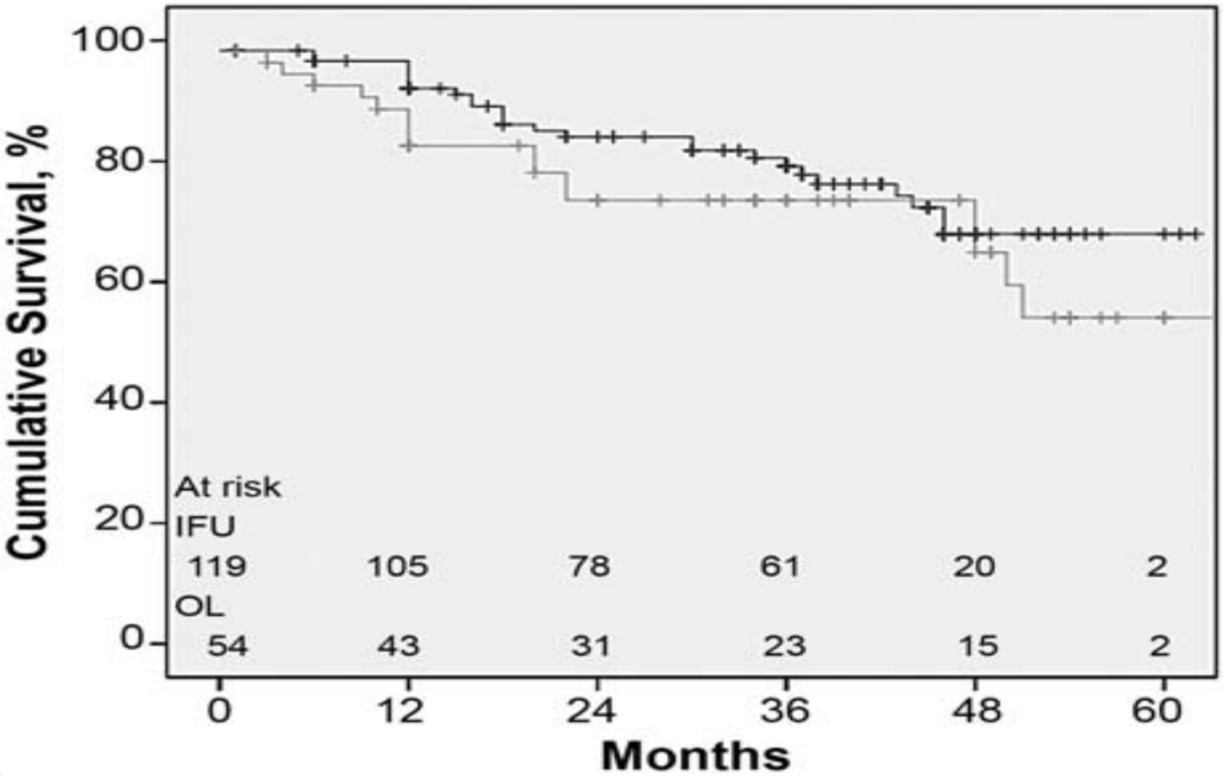

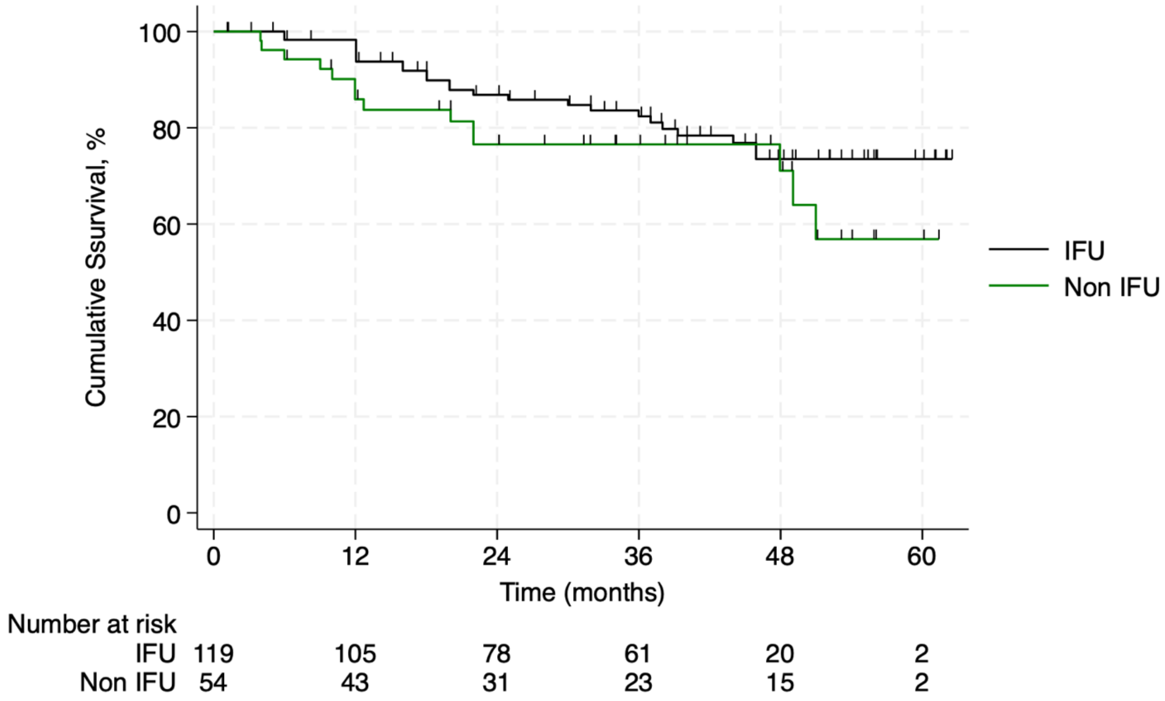

F. Original and regenerated KM of Pecoraro F. et al. [45]

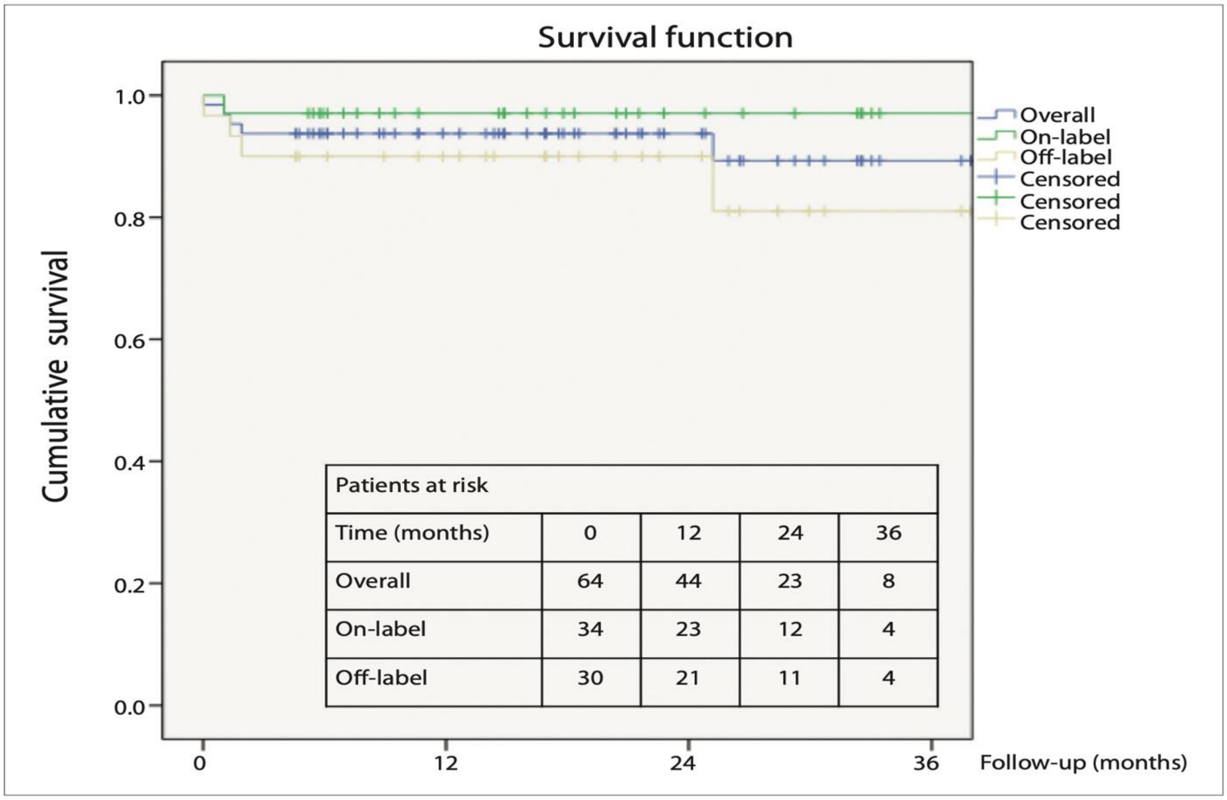

Patients at risk				
Time (months)	0	12	24	36
Overall	64	44	23	8
On-label	34	23	12	4
Off-label	30	21	11	4

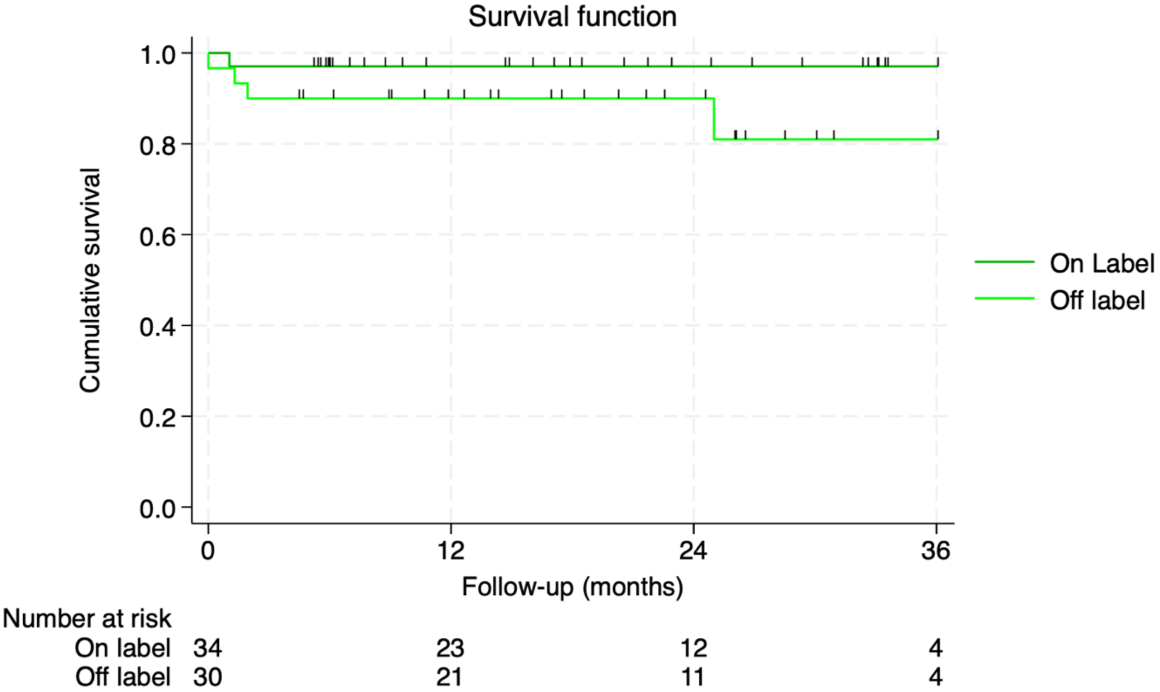

Kaplan–Meier (KM) curves of within IFU versus outside IFU regarding freedom from reintervention

Supplemental Figure S16

A. Original and regenerated KM of Troisi N. et al. [37]

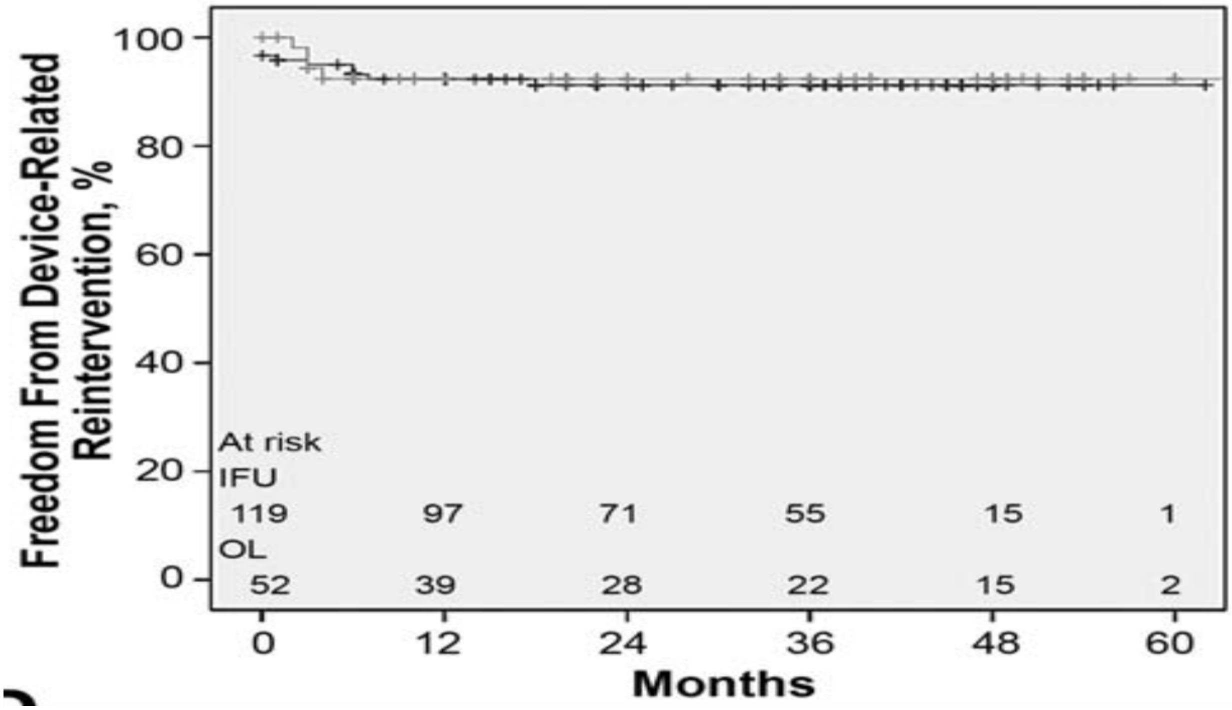

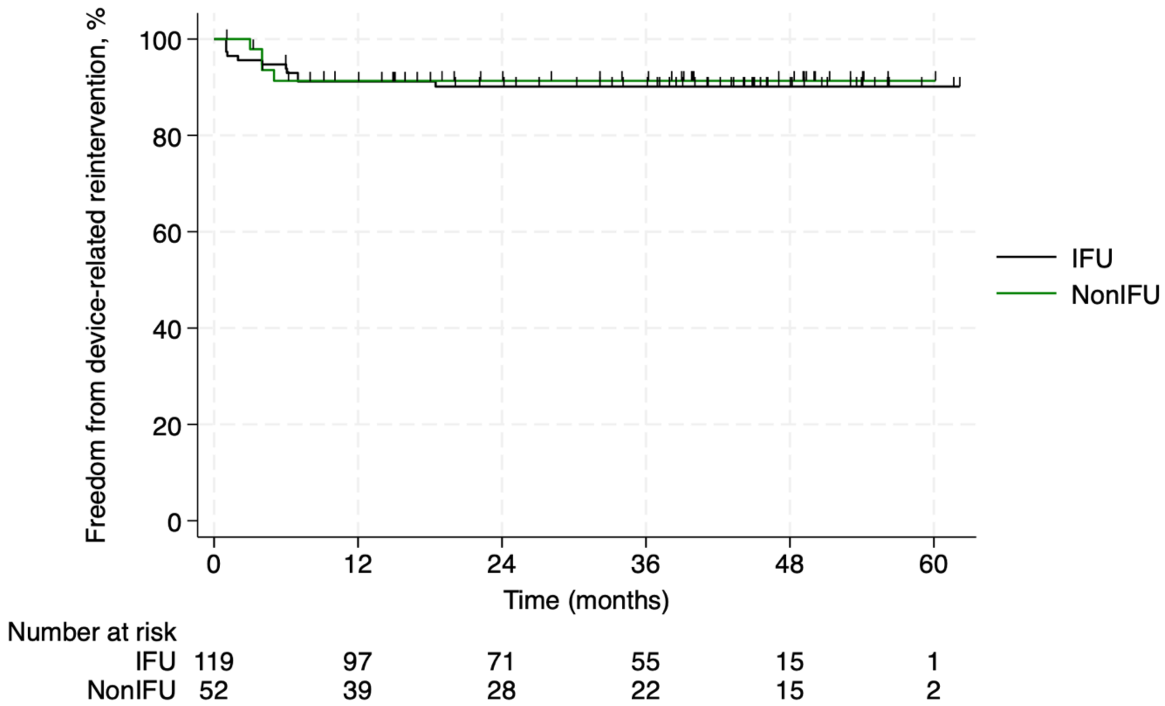

B. Original and regenerated KM of ENGAGE Registry. [5]

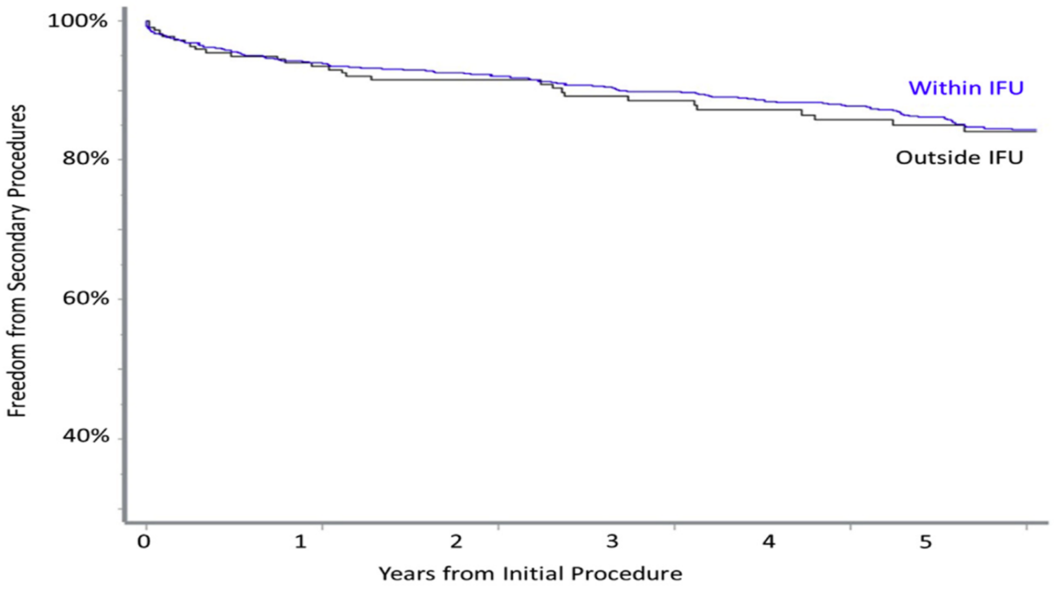

	Outside IFU						Within IFU					
Time Interval	0-30 days	31-365 days	366-731 days	732-1096 days	1097-1461 days	1462-1826 days	0-30 days	31-365 days	366-731 days	732-1096 days	1097-1461 days	1462-1826 days
No. at Risk[1]	224	214	190	161	137	114	1033	1000	891	760	667	571
No. of Events	4	10	4	5	4	2	21	41	17	17	15	20
No. Censored[2]	6	14	25	19	19	34	12	68	114	76	81	224
KM Estimate[3]	0.982	0.935	0.915	0.885	0.858	0.841	0.98	0.938	0.92	0.898	0.877	0.843
Peto SE	0.009	0.017	0.020	0.025	0.030	0.034	0.004	0.008	0.009	0.011	0.013	0.017

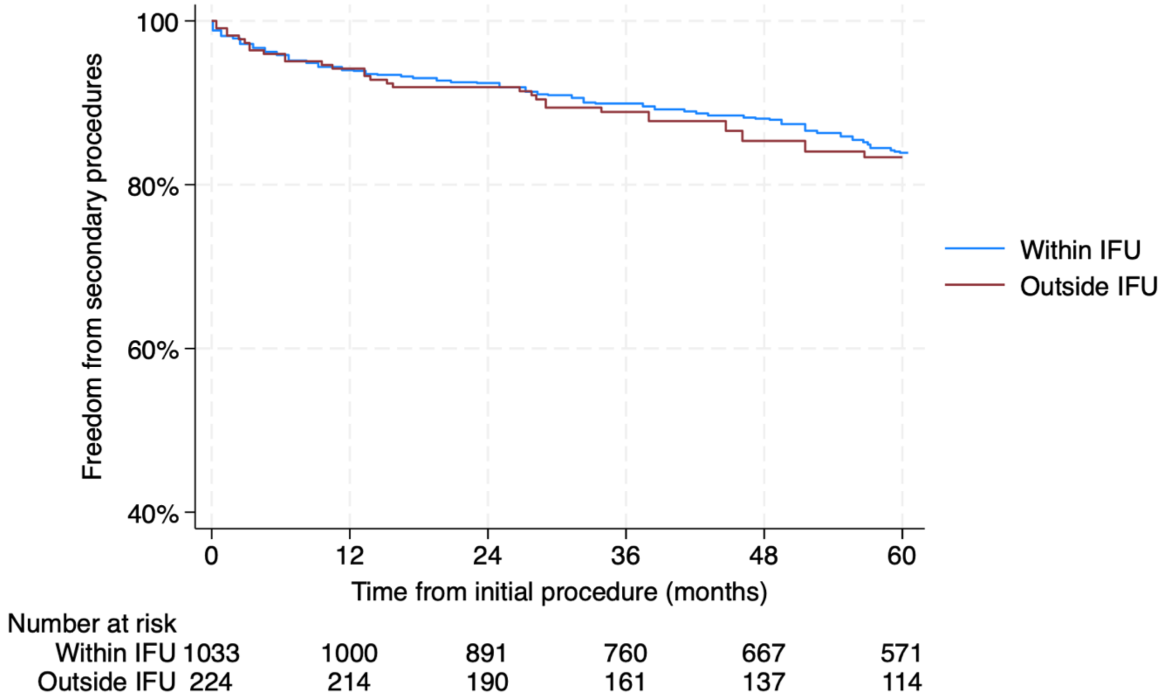

C. Original and regenerated KM of Vedani S.M. et al. [40]

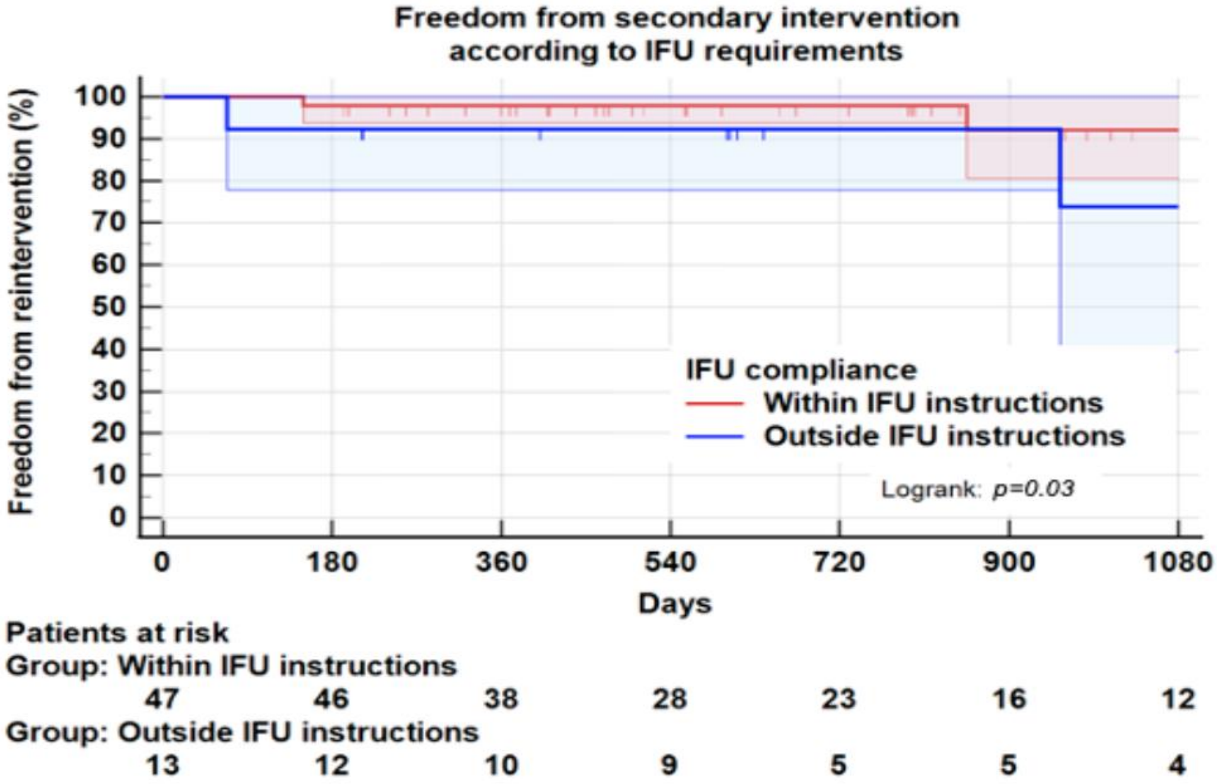

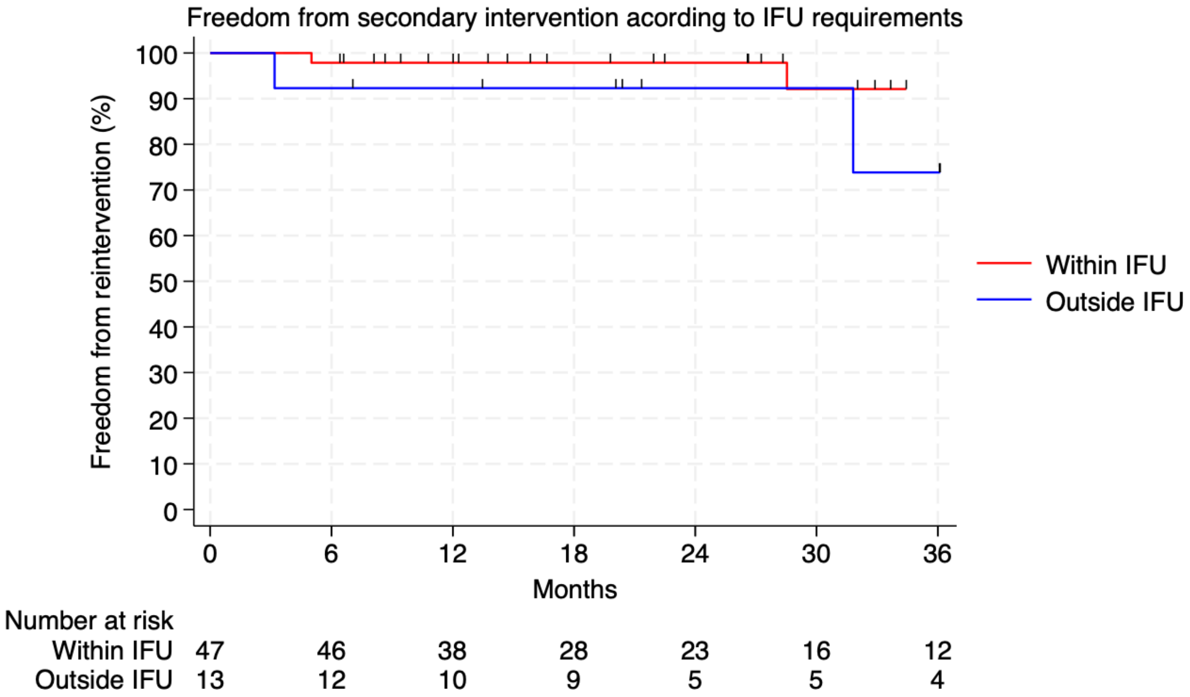

D. Original and regenerated KM of Matsagkas M. et al. [41]

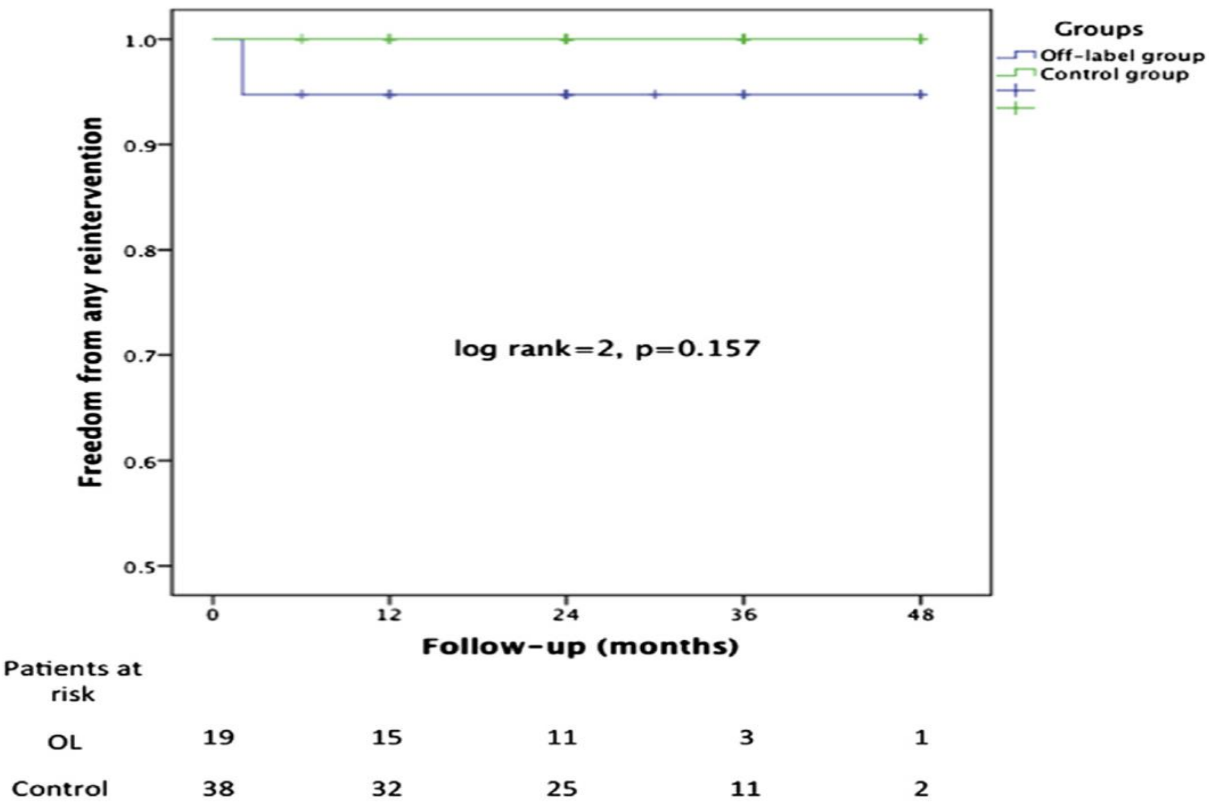

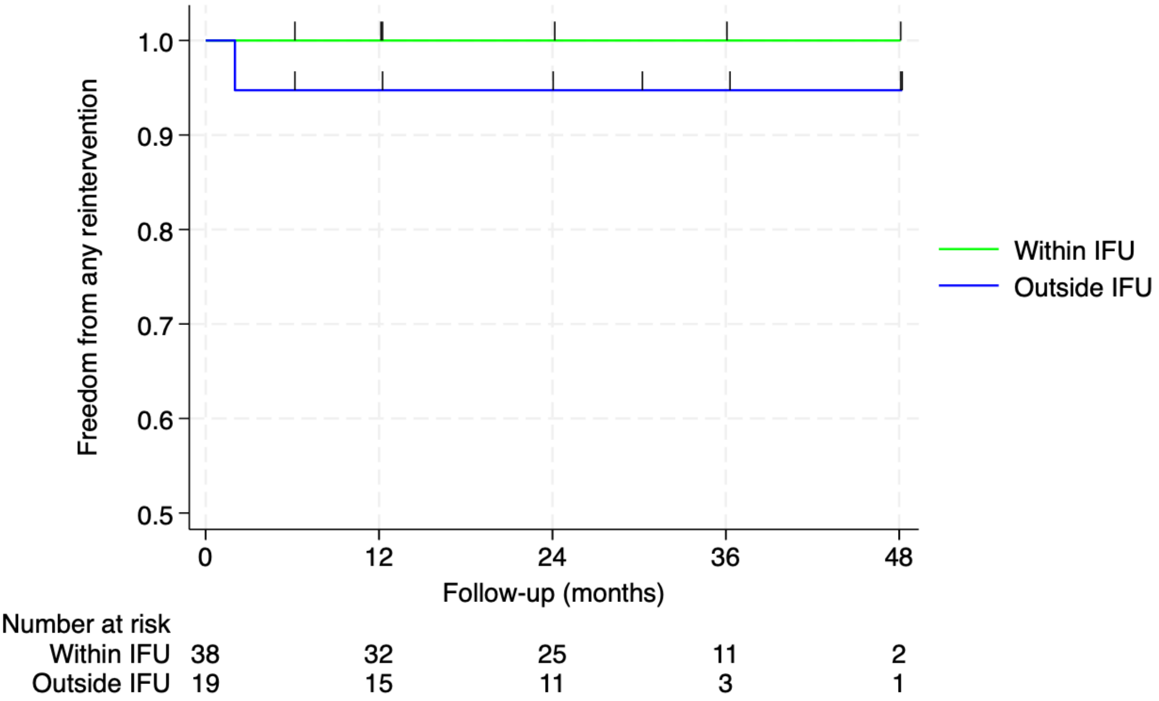

E. Original and regenerated KM of Georgiadis S.G. et al. [43]

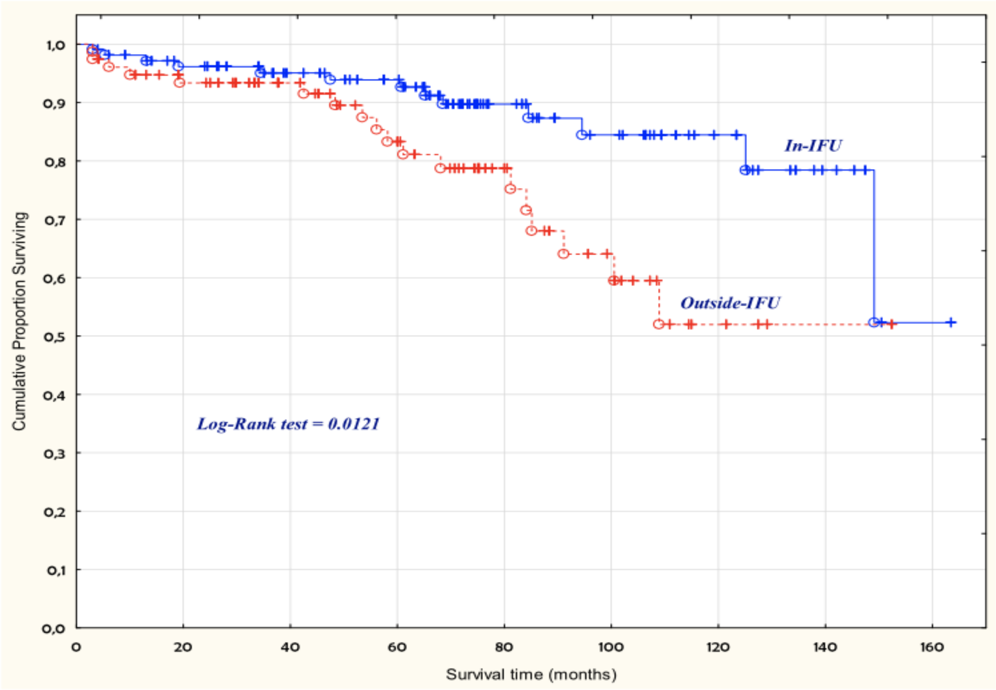

Time (months)	24	36	48	60	72	84	96	120
N at risk (In-IFU)	96	87	79	74	54	39	30	16
N at risk (Outside-IFU)	65	55	47	40	31	21	15	4

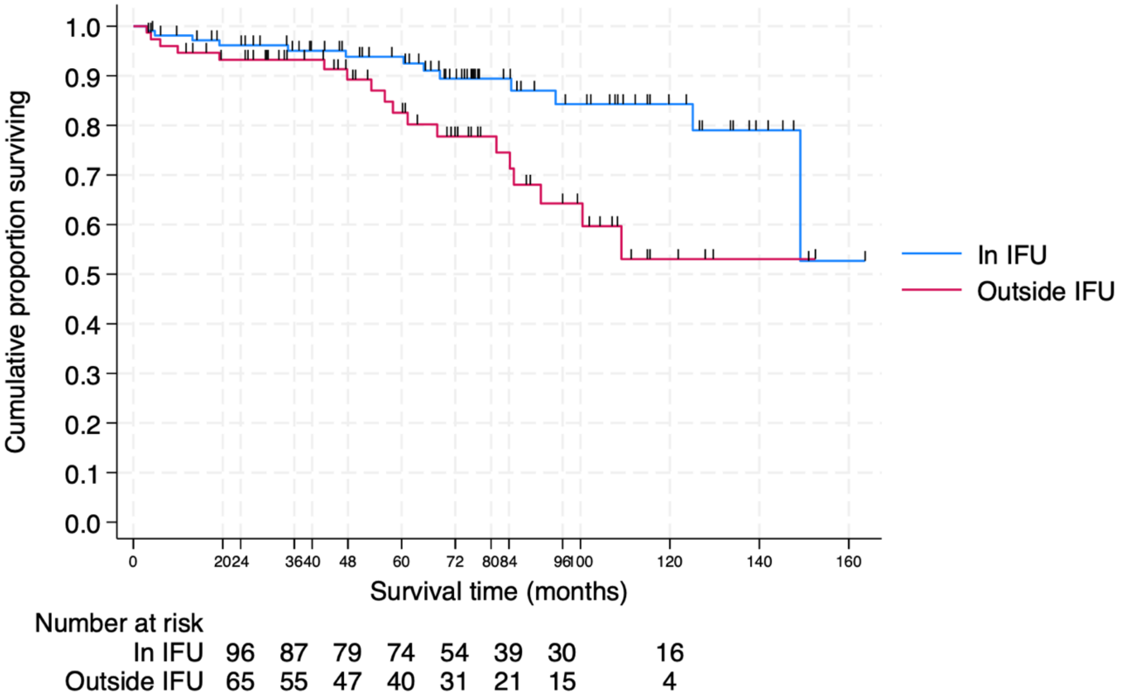

F. Original and regenerated KM of Pecoraro F. et al. [45]

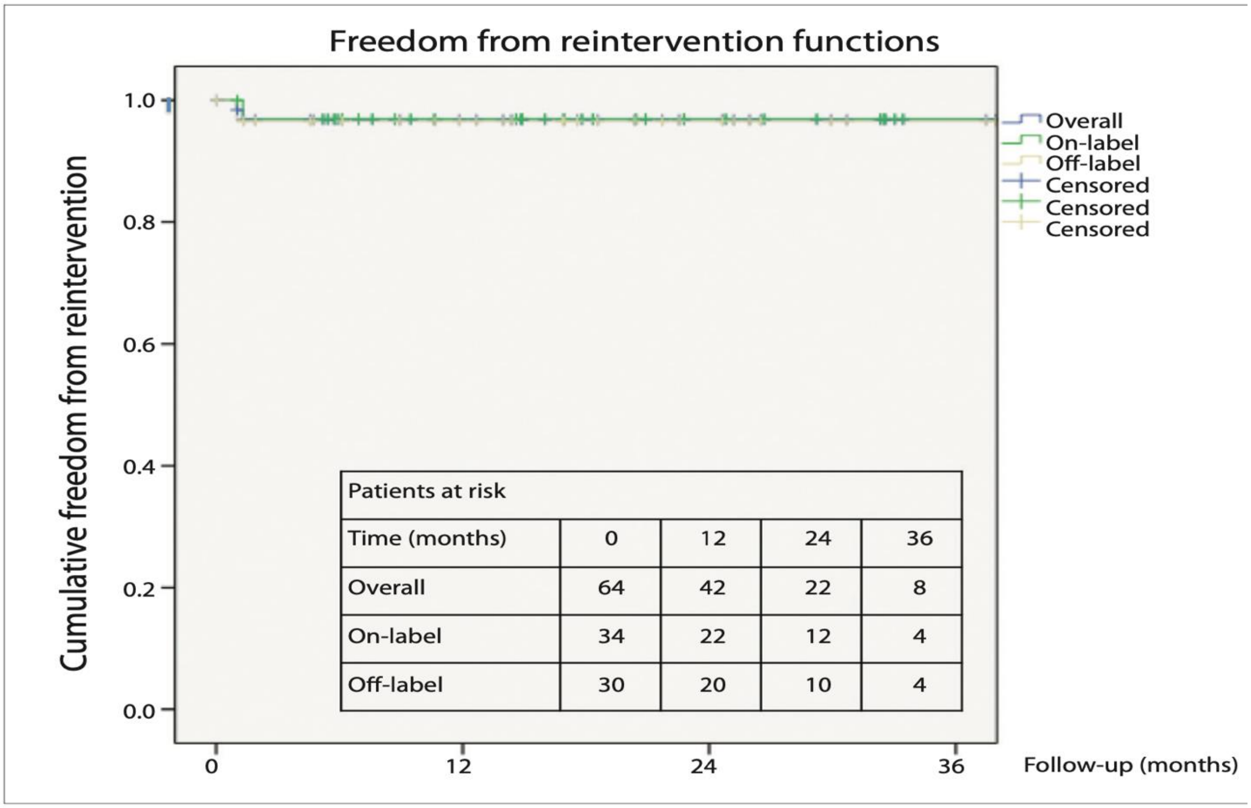

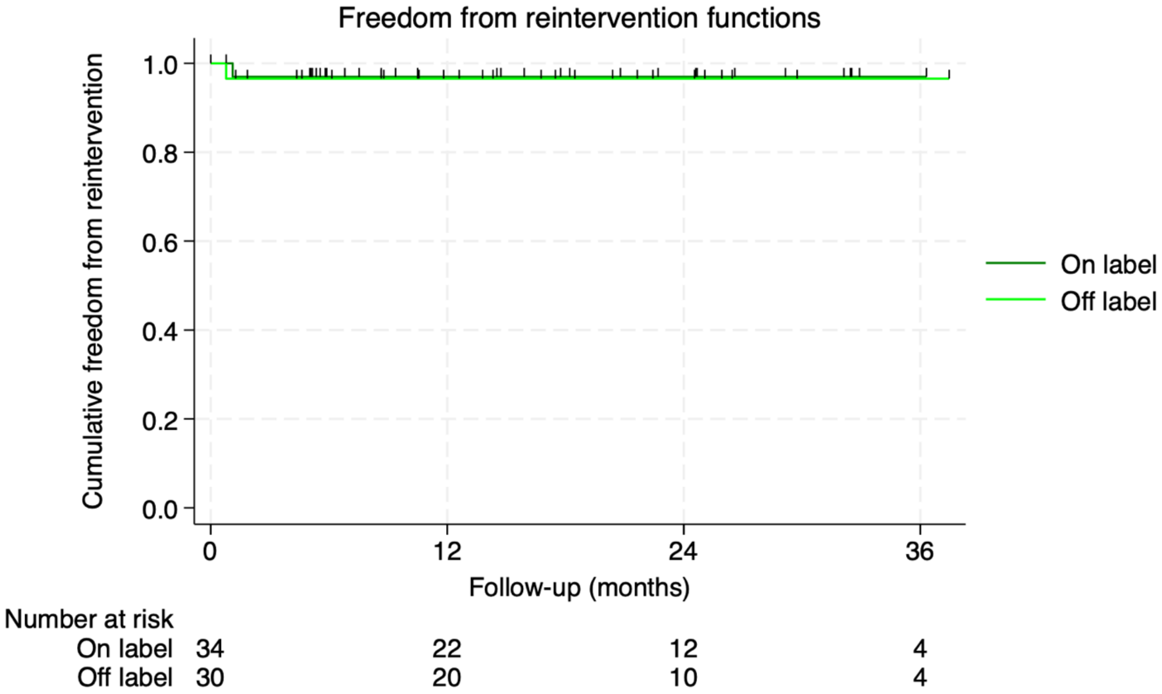

Kaplan–Meier (KM) curves of within IFU versus outside IFU regarding aneurysm–related mortality

Supplementary Figure S17

A. Original and regenerated KM of ENGAGE Registry. [5]

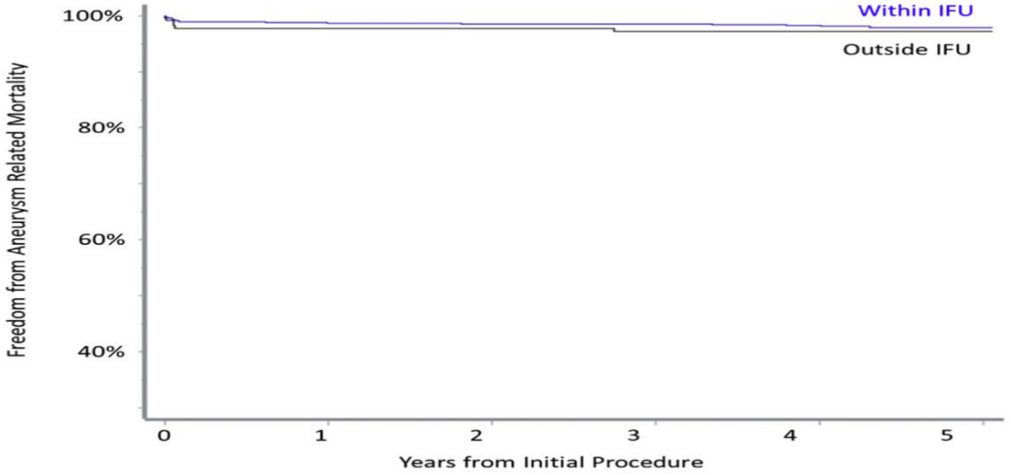

	Outside IFU						Within IFU					
Time Interval	0-30 days	31-365 days	366-731 days	732-1096 days	1097-1461 days	1462-1826 days	0-30 days	31-365 days	366-731 days	732-1096 days	1097-1461 days	1462-1826 days
No. at Risk[1]	225	219	203	177	153	131	1038	1024	947	821	740	648
No. of Events	5	0	0	1	0	0	11	3	1	0	3	1
No. Censored[2]	1	16	26	23	22	43	3	74	125	81	89	272
KM Estimate[3]	0.978	0.978	0.978	0.972	0.972	0.972	0.989	0.986	0.985	0.985	0.981	0.979
Peto SE	0.010	0.010	0.010	0.013	0.013	0.013	0.003	0.004	0.004	0.004	0.005	0.006

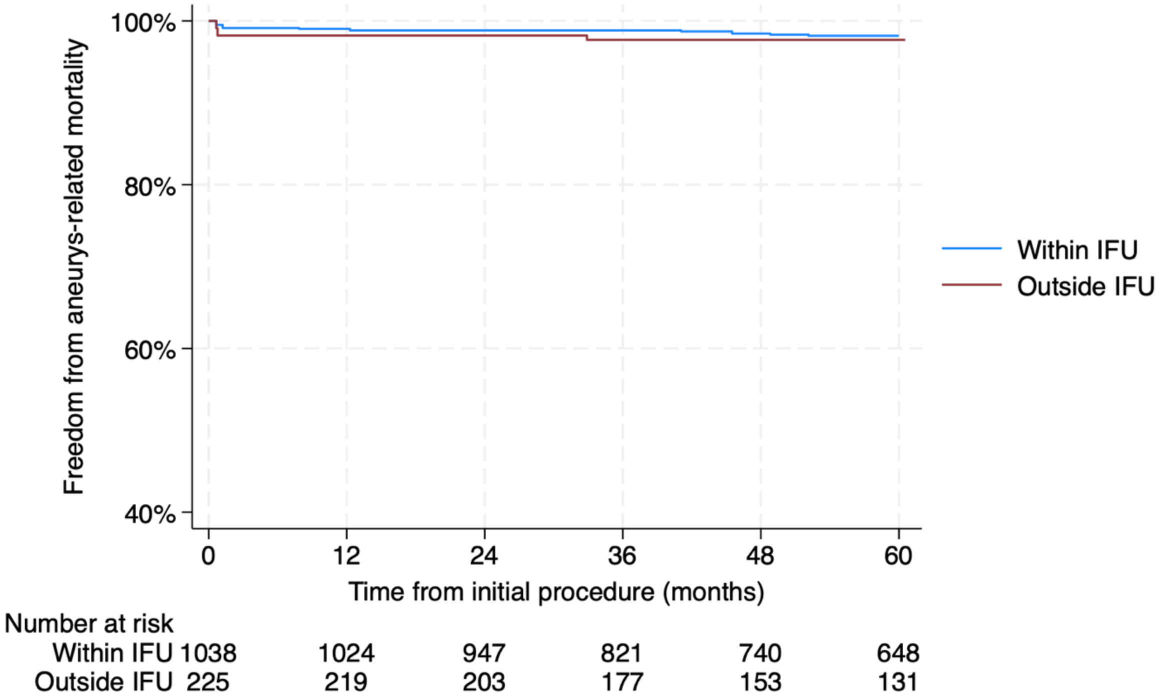

B. Regenerated KM of Özdemir-van Brunschot D.M.D. et al. [35]

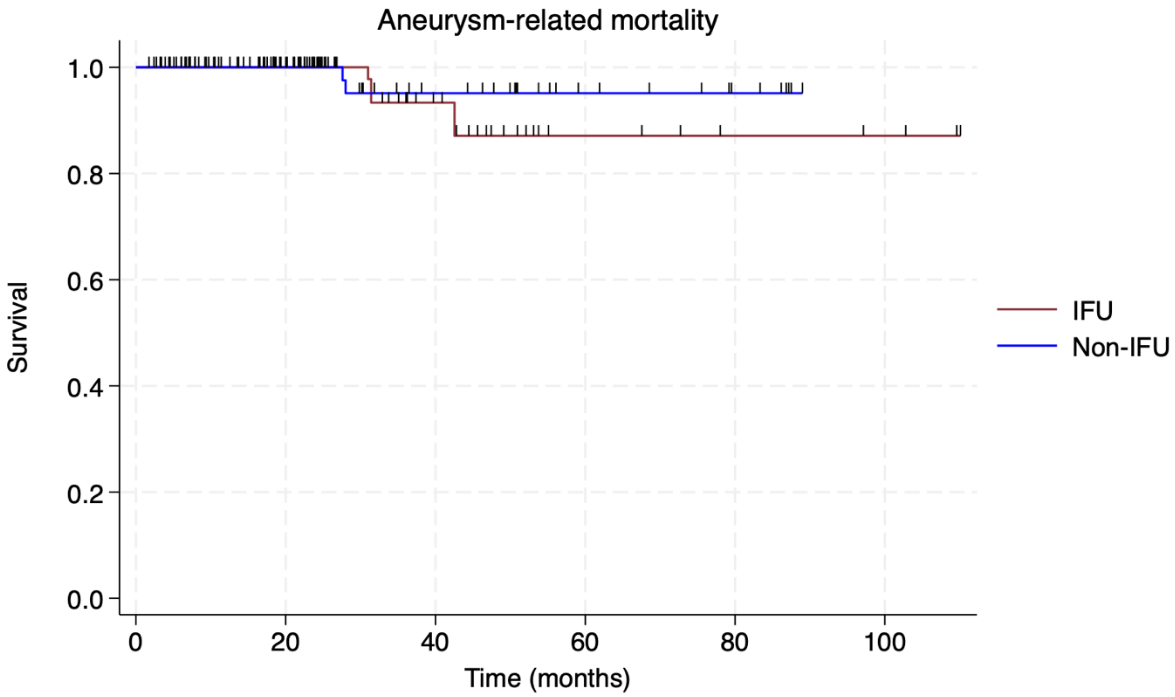

Supplement: Supplementary file 1 [file jcm-14-06453-s001.zip › Supplemental Figures S15-S17.pdf]
