# Supplementary material for: Endurant Stents in Abdominal Aortic Aneurysm Repair: A Systematic Review and Meta-Analysis
Source: J Clin Med. 2025 Sep 12;14(18):6453. doi: 10.3390/jcm14186453 (PMC12470529; doi:10.3390/jcm14186453)

Supplemental Figure S18.

A. Kaplan–Meier curve of reconstructed IPD, regarding the overall survival, involving 1448 patients treated within versus 470 treated outside the IFU

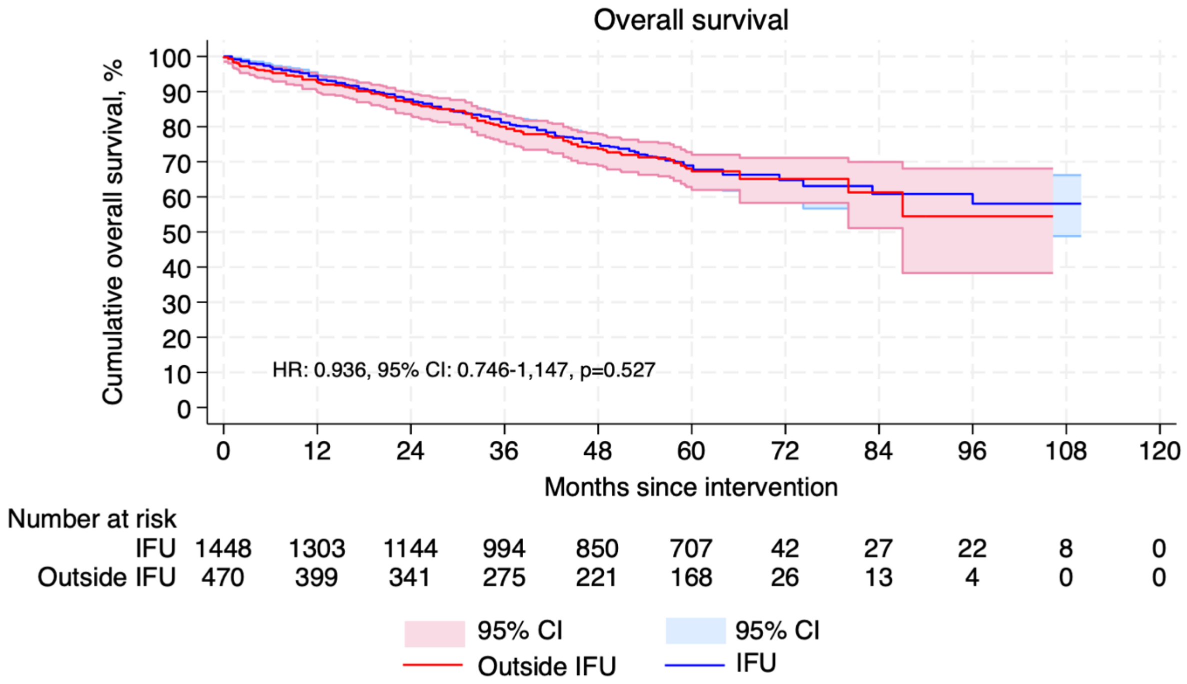

B. Forest plot based on the pooled HRs of the included studies comparing patients treated IFU versus outside IFU

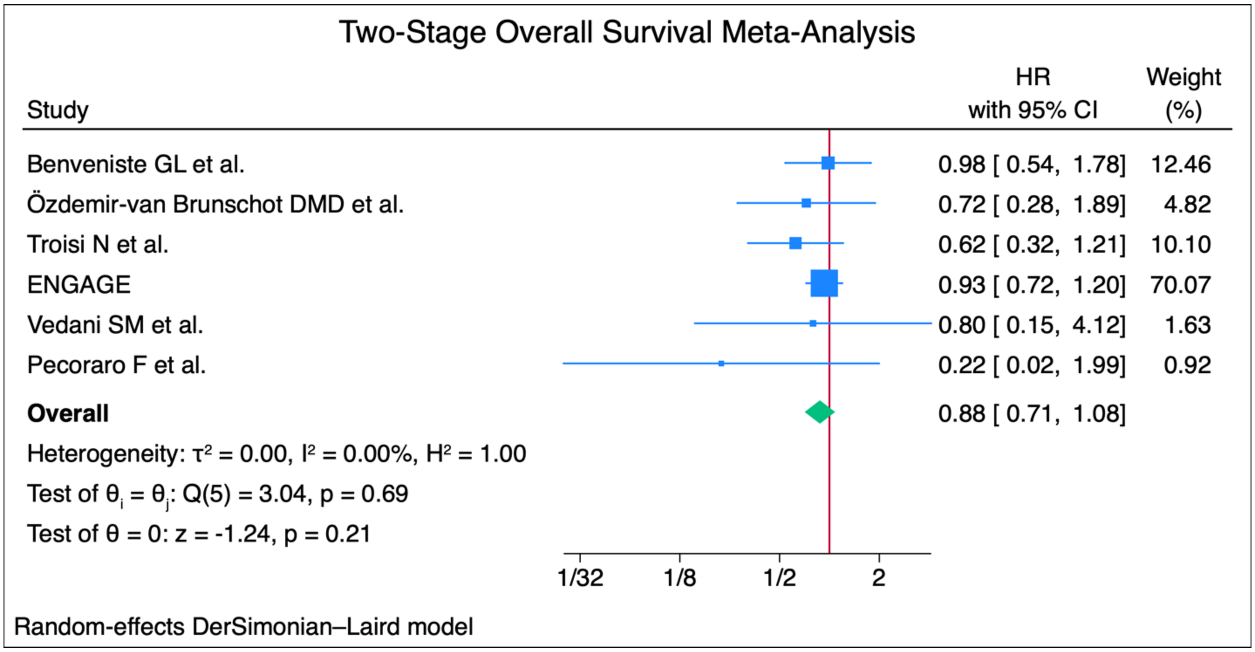

Supplemental Figure S19.

A. Kaplan–Meier curve of reconstructed IPD, regarding the aneurysm–related mortality, involving 1142 patients treated within versus 299 treated outside the IFU

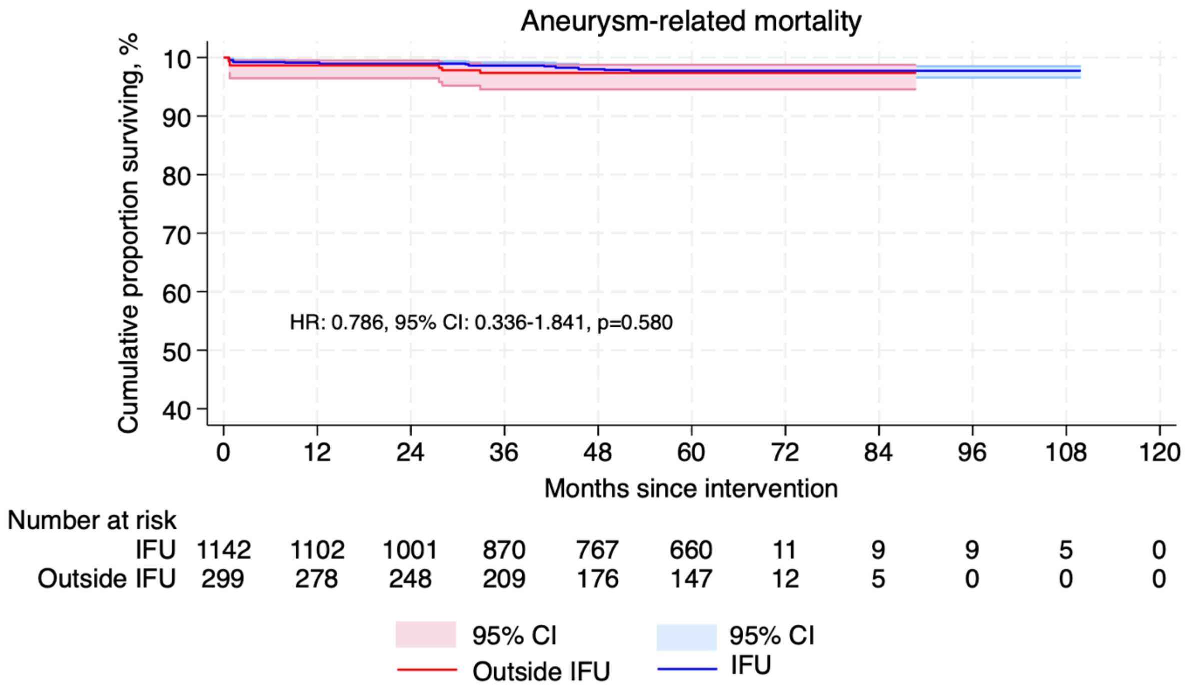

B. Forest plot based on the pooled HRs of the included studies comparing patients treated IFU versus outside IFU

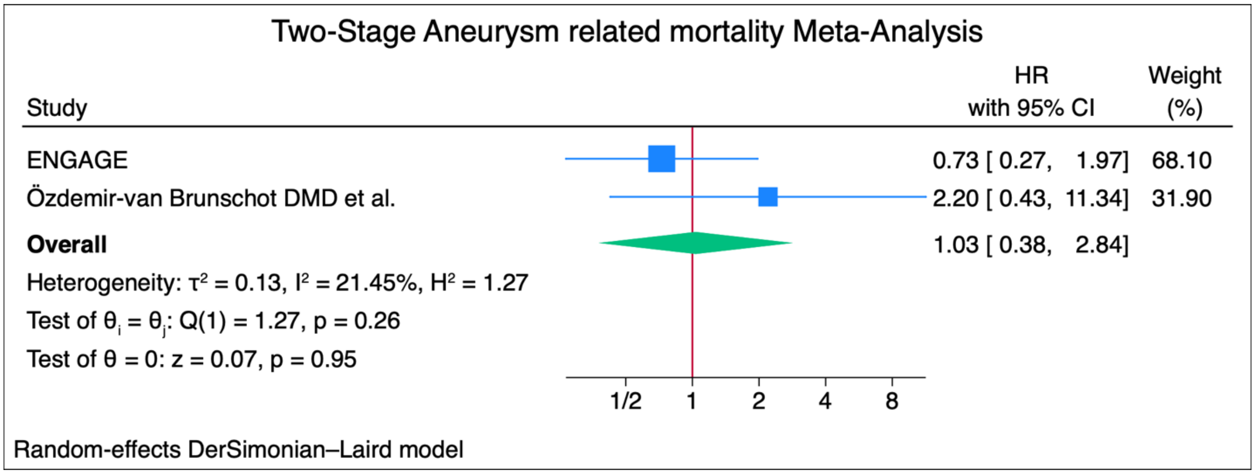

Supplemental Figure S20.

A. Kaplan–Meier curve of reconstructed IPD, regarding the freedom from secondary intervention, involving 1378 patients treated within versus 415 treated outside the IFU

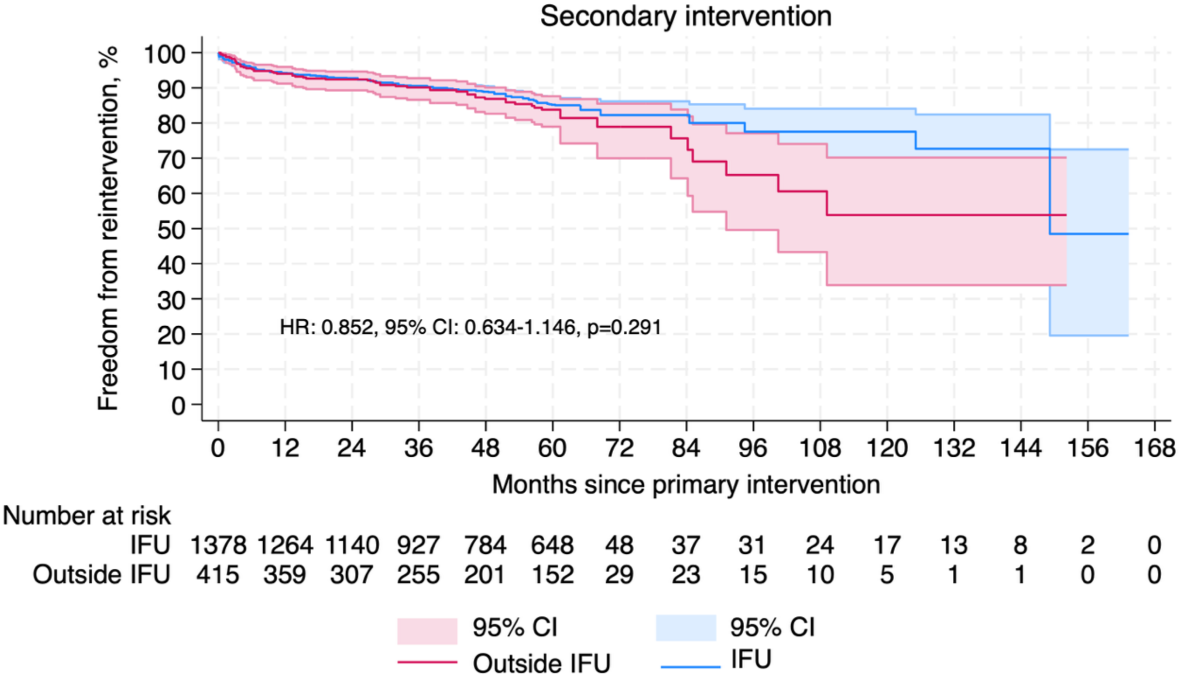

B. Forest plot based on the pooled HRs of the included studies comparing patients treated IFU versus outside IFU

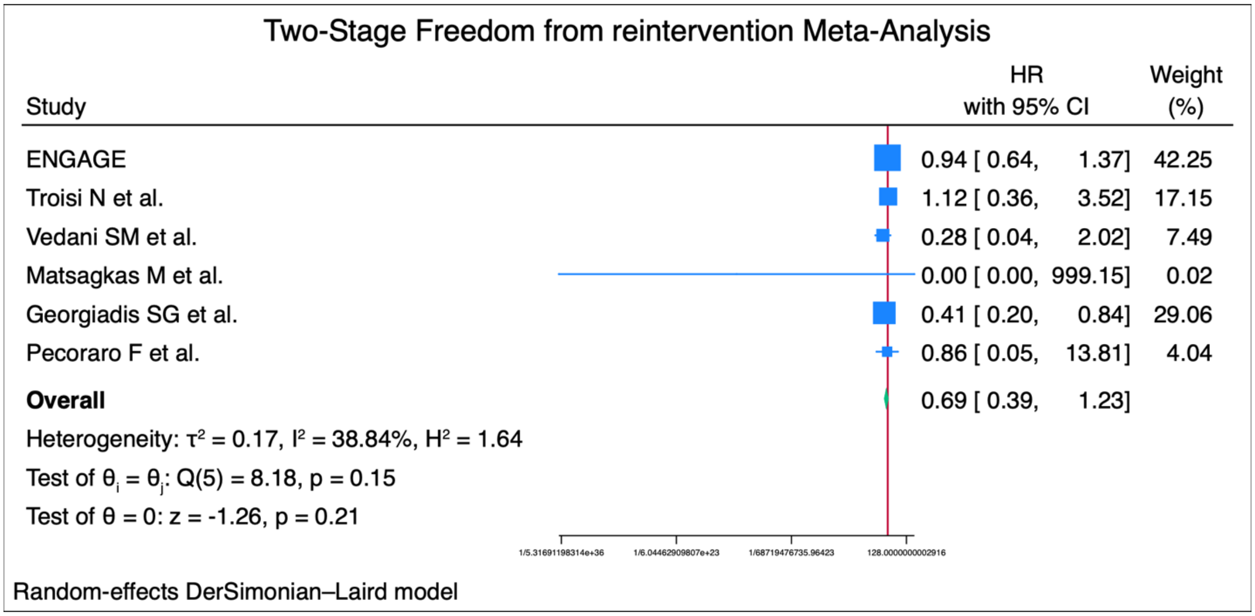

Supplemental Figure S21.

A. Kaplan–Meier curve of reconstructed IPD, regarding the freedom from secondary intervention, after excluding the low-quality studies.

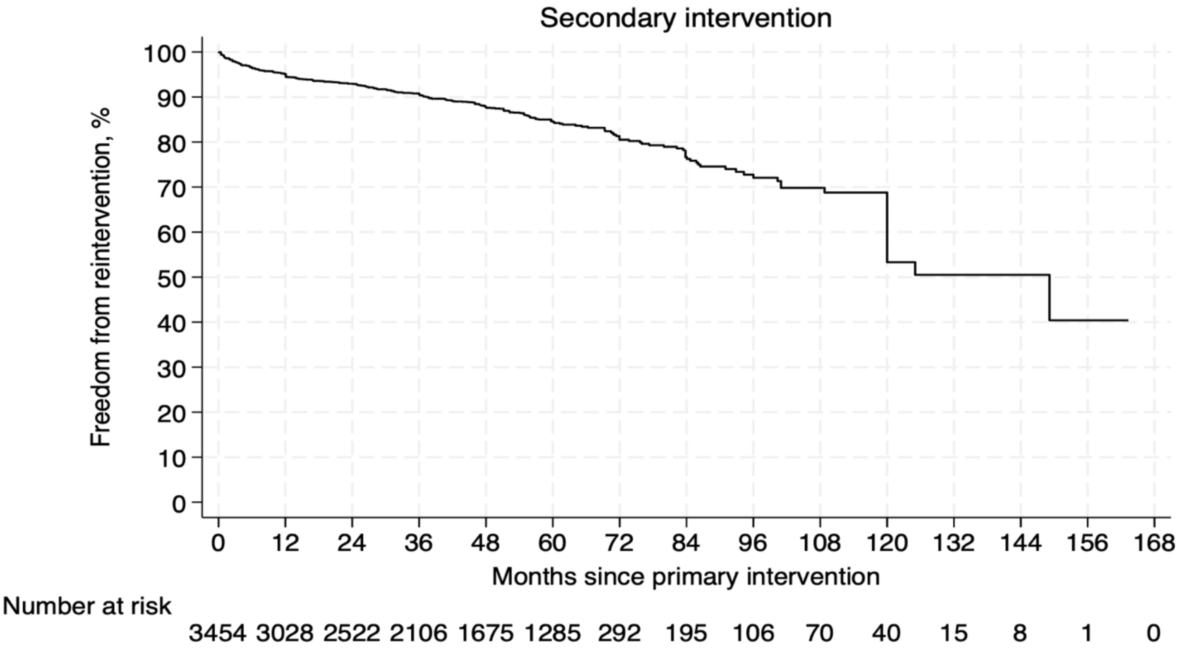

B. Kaplan–Meier curve of reconstructed IPD, regarding overall survival, after excluding the low-quality studies.

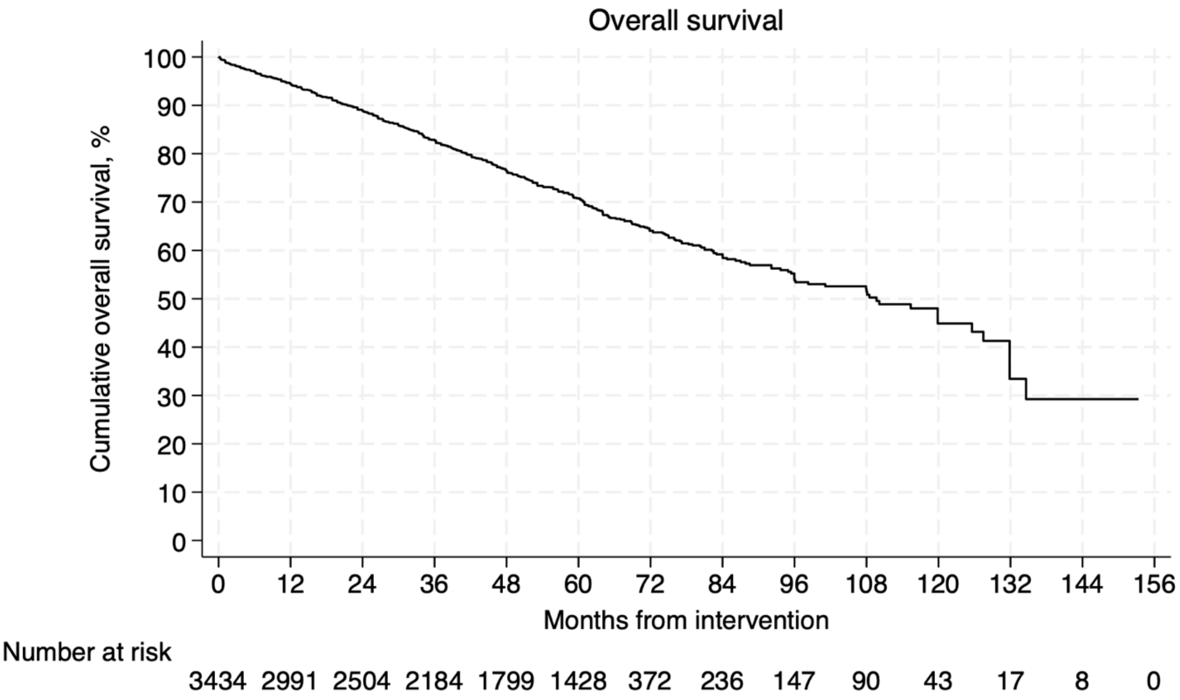

Supplement: Supplementary file 1 [file jcm-14-06453-s001.zip › Supplemental Figures S18-S21.pdf]
