# Supplementary material for: Endurant Stents in Abdominal Aortic Aneurysm Repair: A Systematic Review and Meta-Analysis
Source: J Clin Med. 2025 Sep 12;14(18):6453. doi: 10.3390/jcm14186453 (PMC12470529; doi:10.3390/jcm14186453)
Supplement: Supplementary file 1 [file jcm-14-06453-s001.zip › Supplemental Figures S1-S7.pdf]

Supplemental Figure S1.
Funnel plot regarding survival

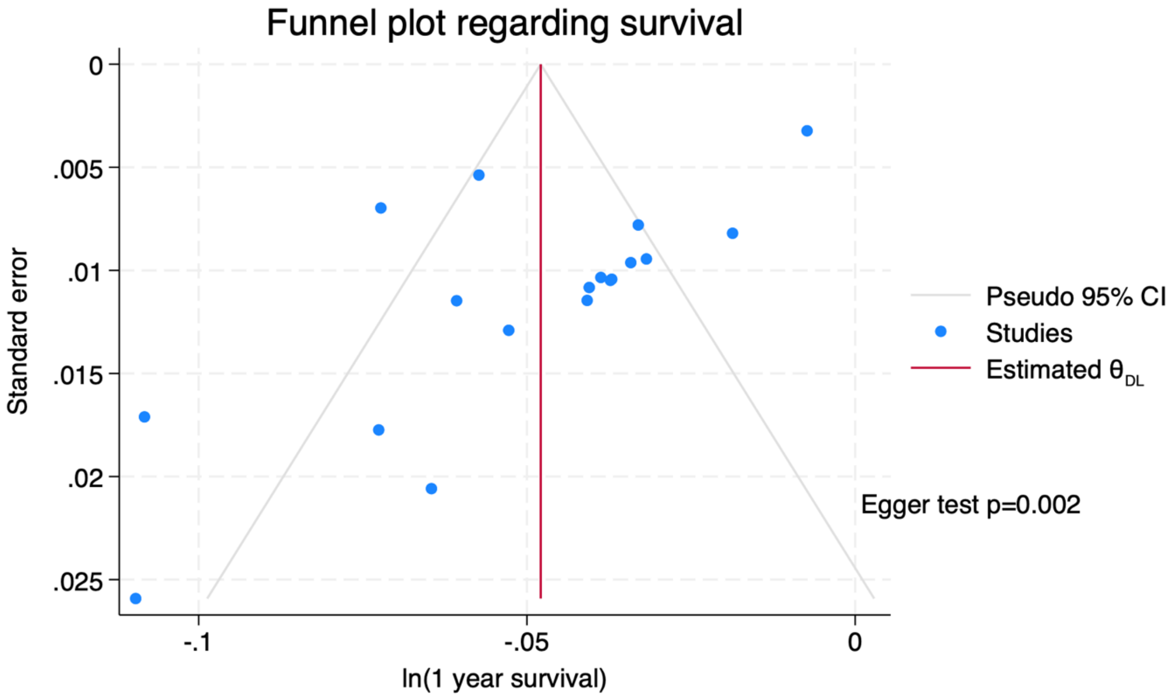

Supplemental Figure S2.
Funnel plot regarding freedom from reintervention

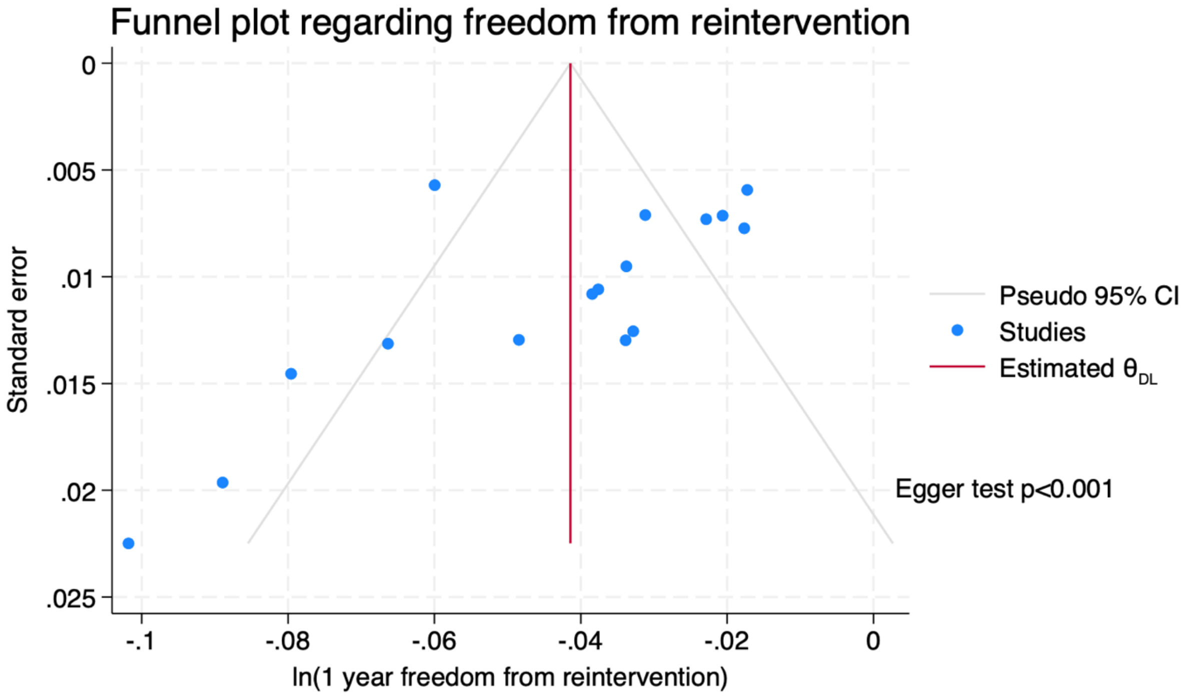

Supplemental Figure S3.

Funnel plot regarding freedom from aneurysm related mortality

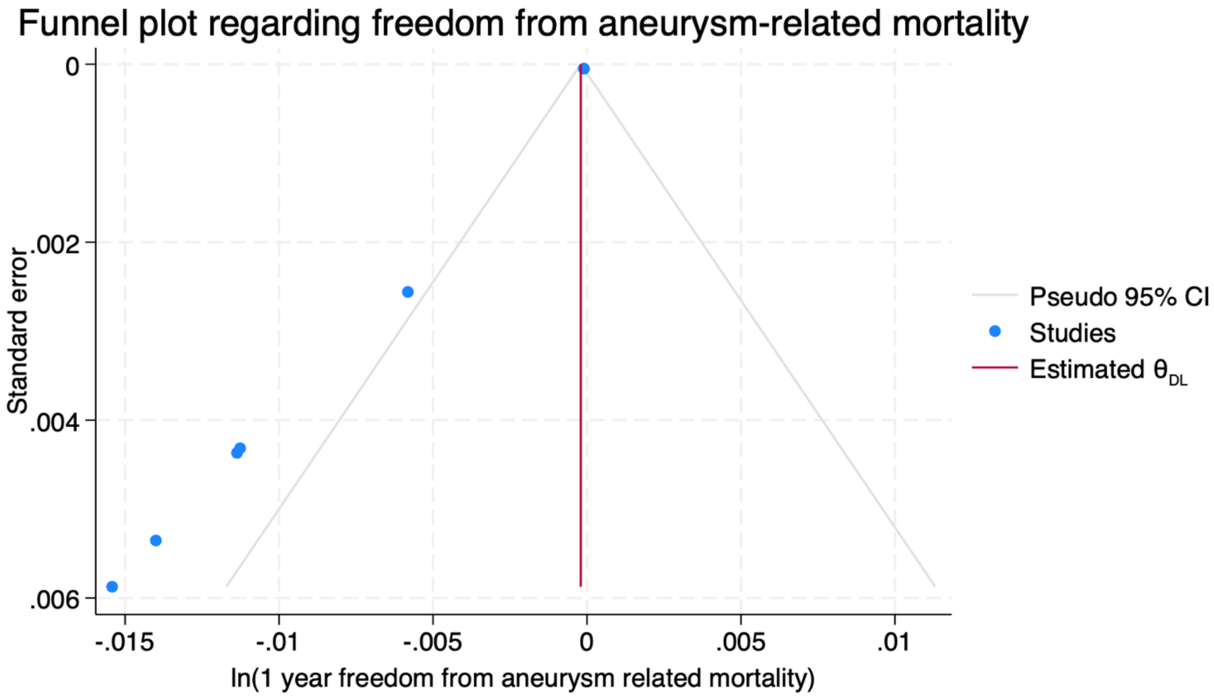

Kaplan–Meier (KM) curves of overall survival probability

Supplemental Figure S4

A. Original and regenerated KM of Mannetje Y.W. et al. [46]

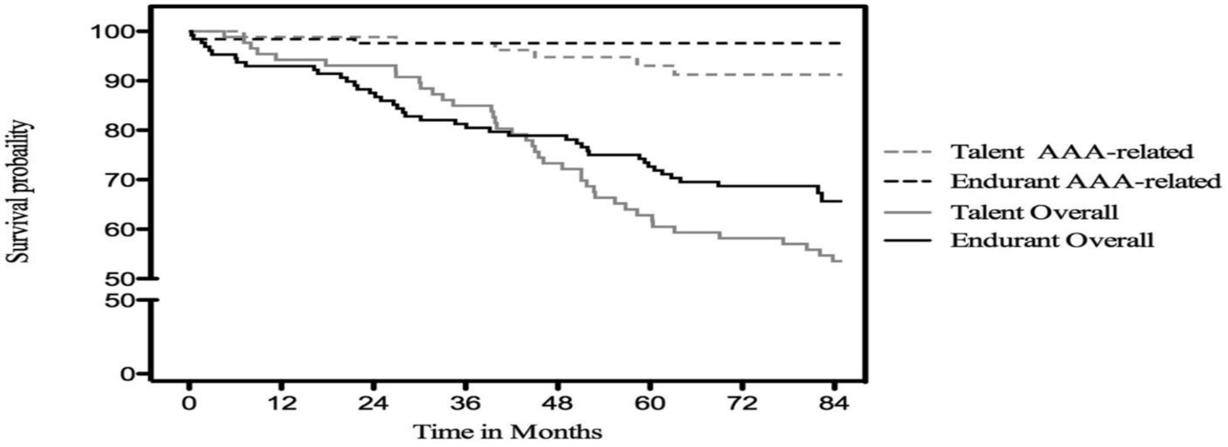

	Day 0	30-days	1-year	5-years	7-years
Talent overall<br>Patients at risk (SE)	90	88<br>(.000)	83<br>(.025)	54<br>(.052)	46<br>(.054)
Endurant overall<br>Patients at risk (SE)	131	126<br>(.011)	119<br>(.023)	93<br>(.039)	32<br>(.045)
Talent AAA<br>Patients at risk (SE)	90	88<br>(.000)	83<br>(.011)	54<br>(.030)	46<br>(.035)
Endurant AAA<br>Patients at risk (SE)	131	126<br>(.022)	119<br>(.011)	93<br>(.014)	32<br>(.014)

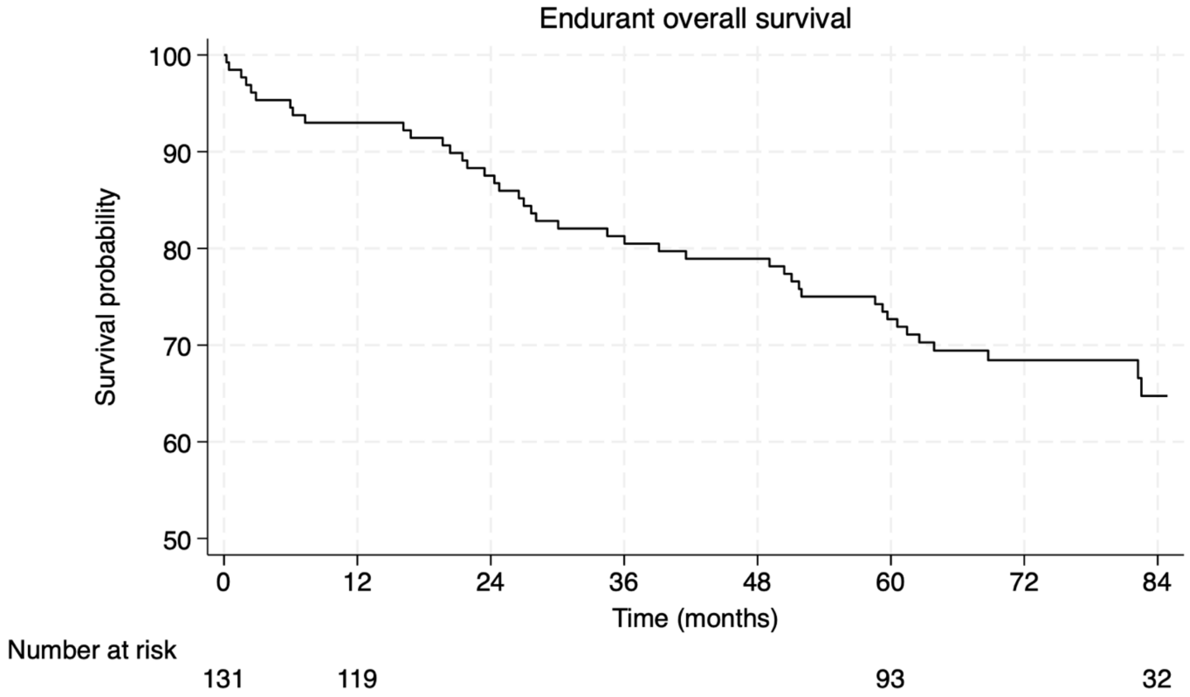

B. Original and regenerated KM of Becquemin J. P. et al. [33]

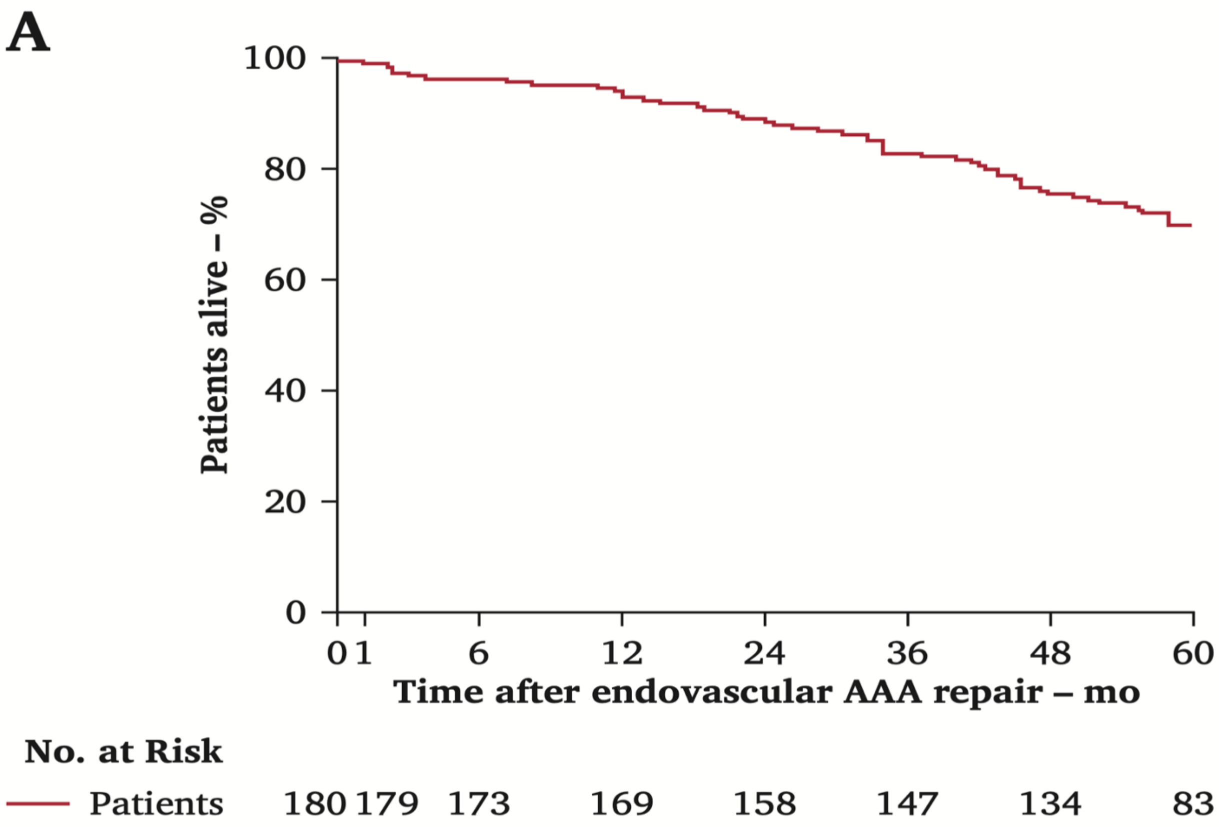

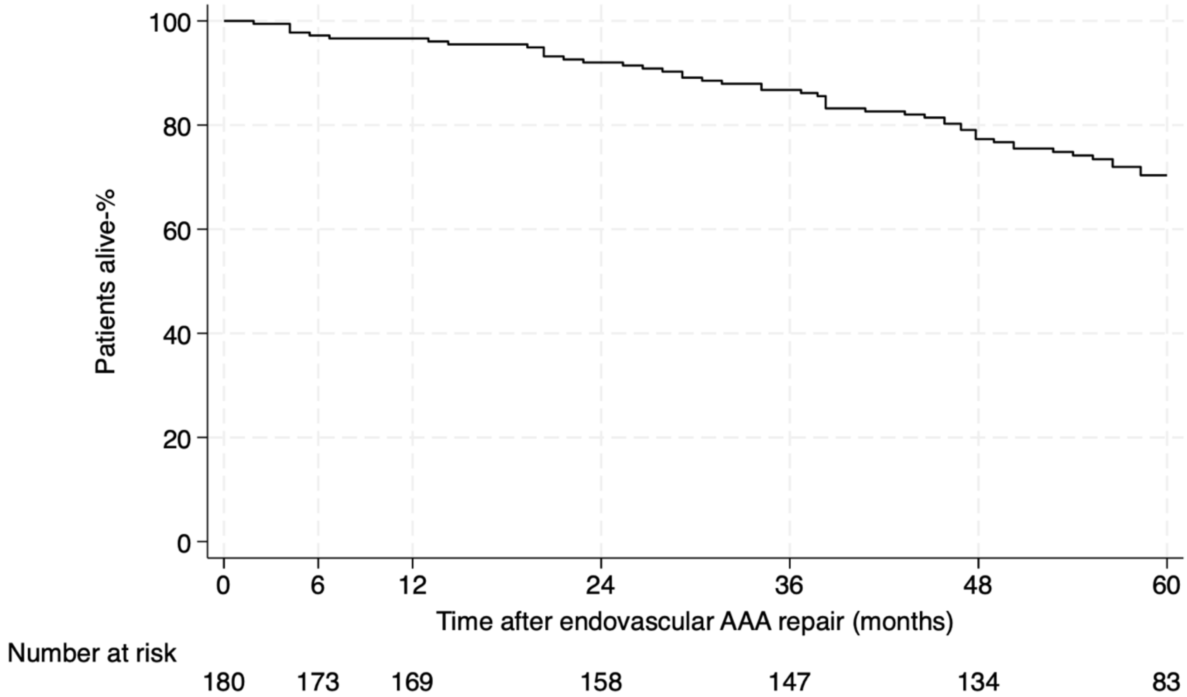

C. Regenerated KM of Benveniste G.L. Et al. [29]

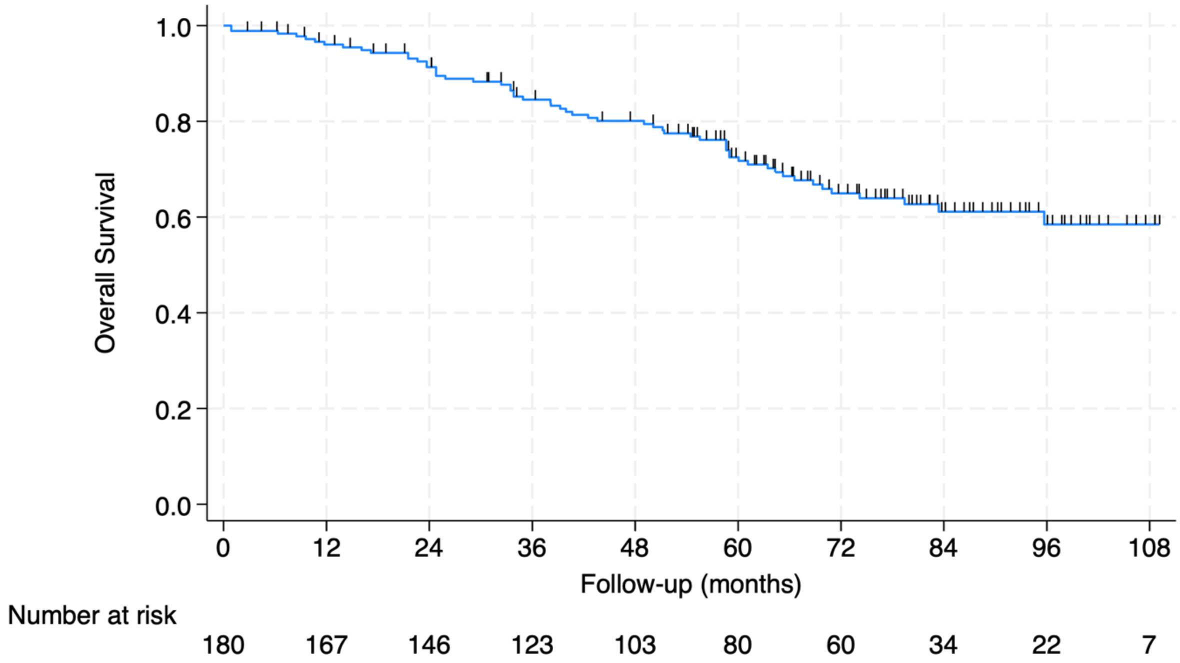

D. Original and regenerated KM of Bisdas T. et al. [36]

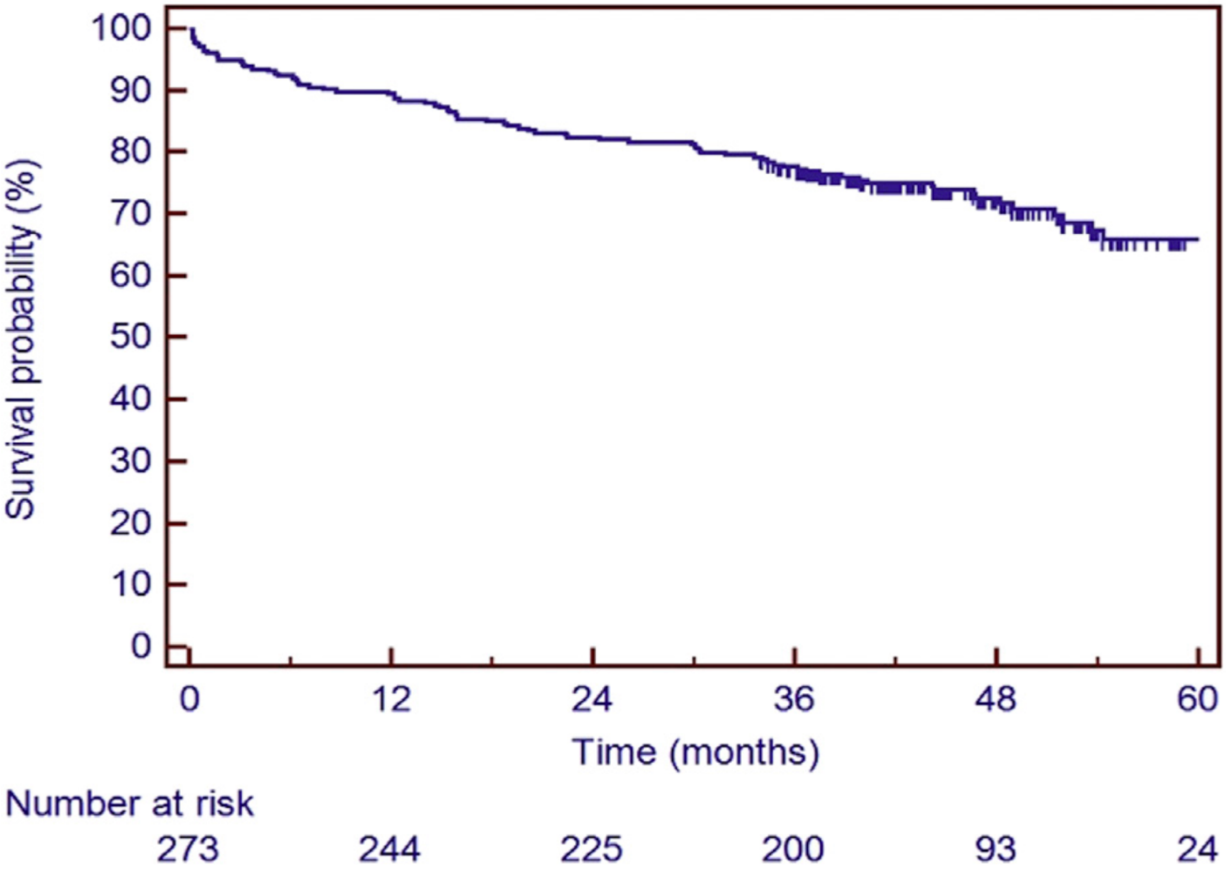
Survival probability (%)
100
90
80
70
60
50
40
30
20
10
0
0
12
24
36
48
60
Time (months)
Number at risk
273
244
225
200
93
24

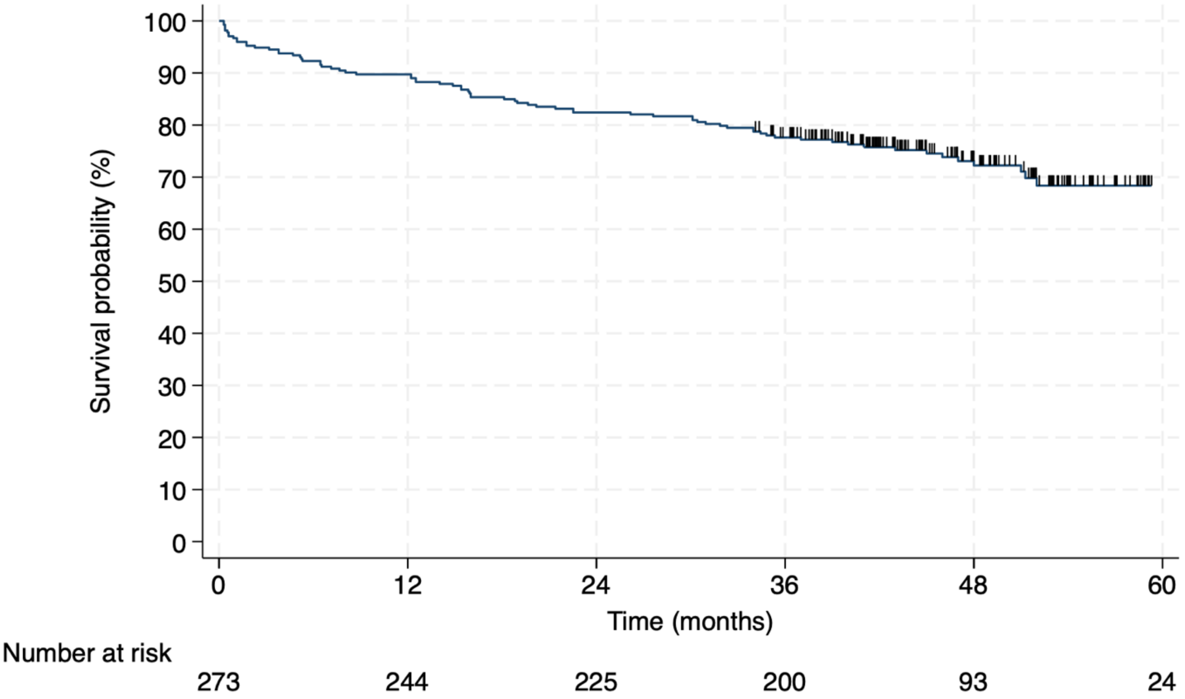
Survival probability (%)
100
90
80
70
60
50
40
30
20
10
0
0
12
24
36
48
60
Time (months)
Number at risk
273
244
225
200
93
24

E. Original and regenerated KM of Deery S.E. et al. [38]

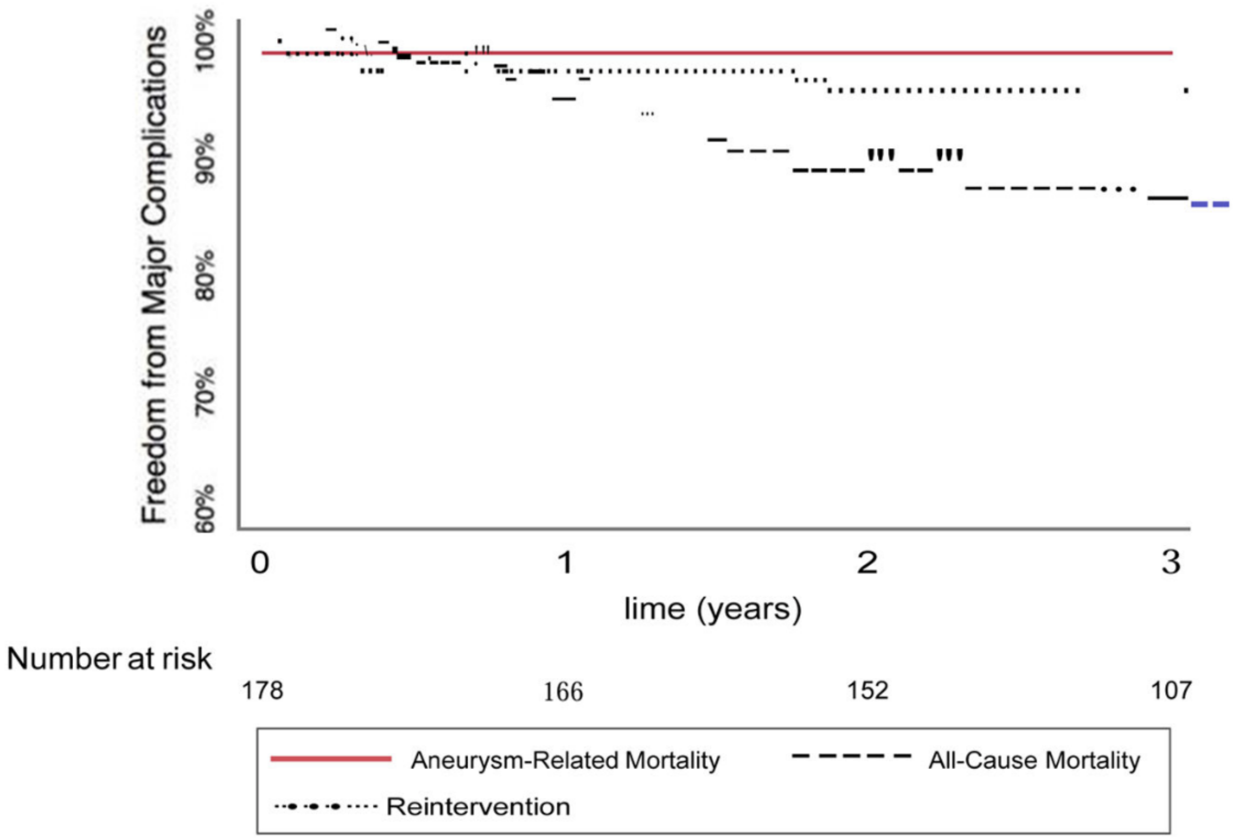

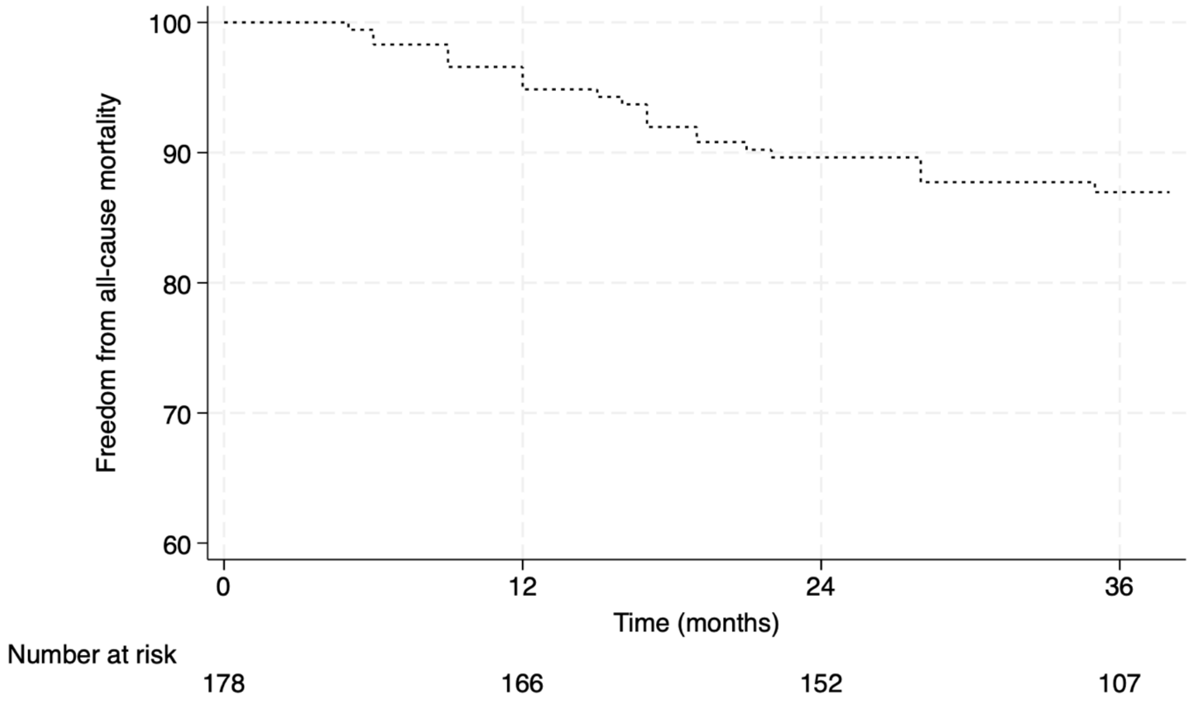

Supplemental Figure S5

A. Original and regenerated KM of ENGAGE registry. [5]

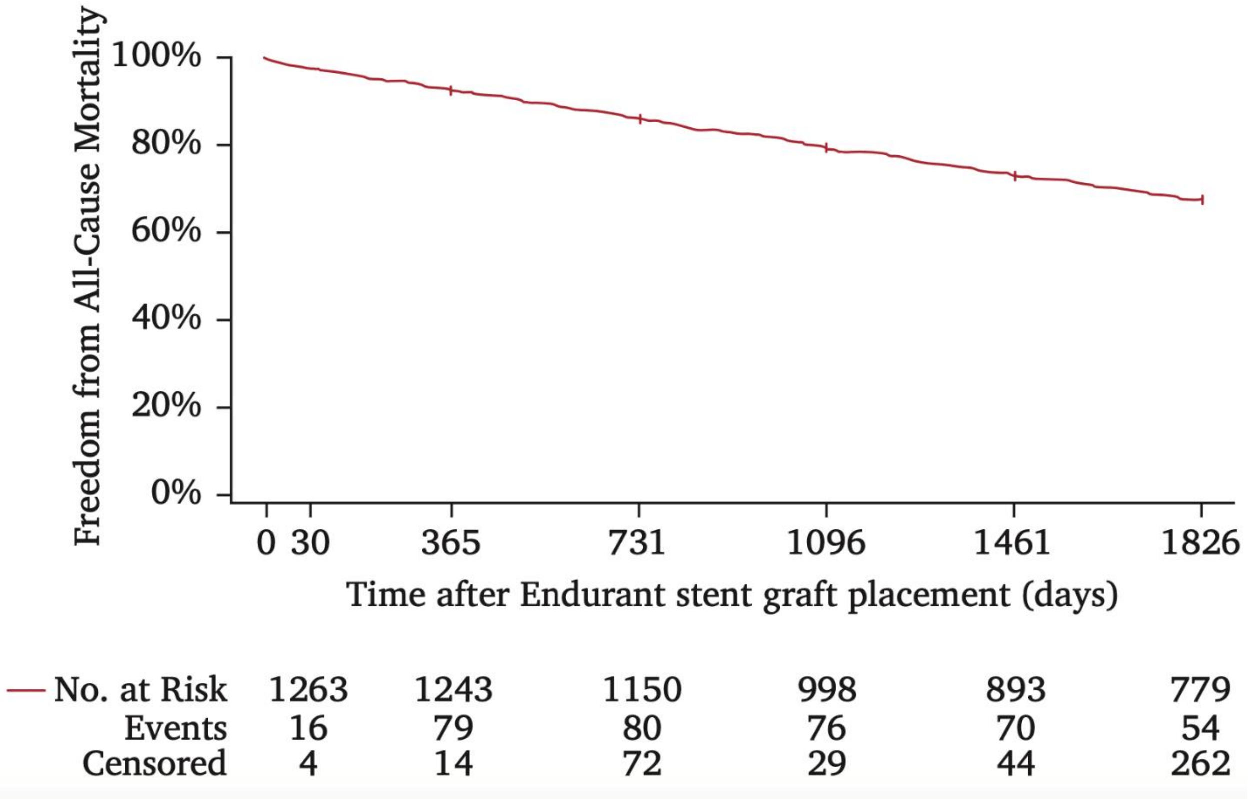

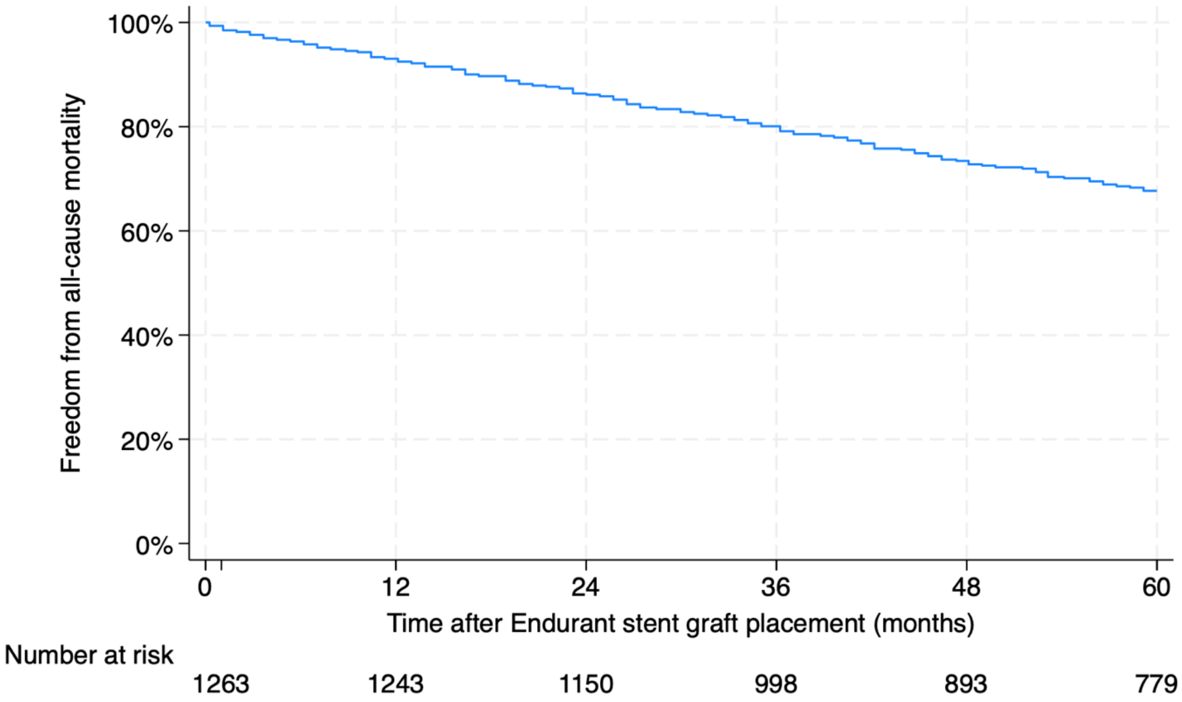

B. Original and regenerated KM of Falster M.O. et al. [30]

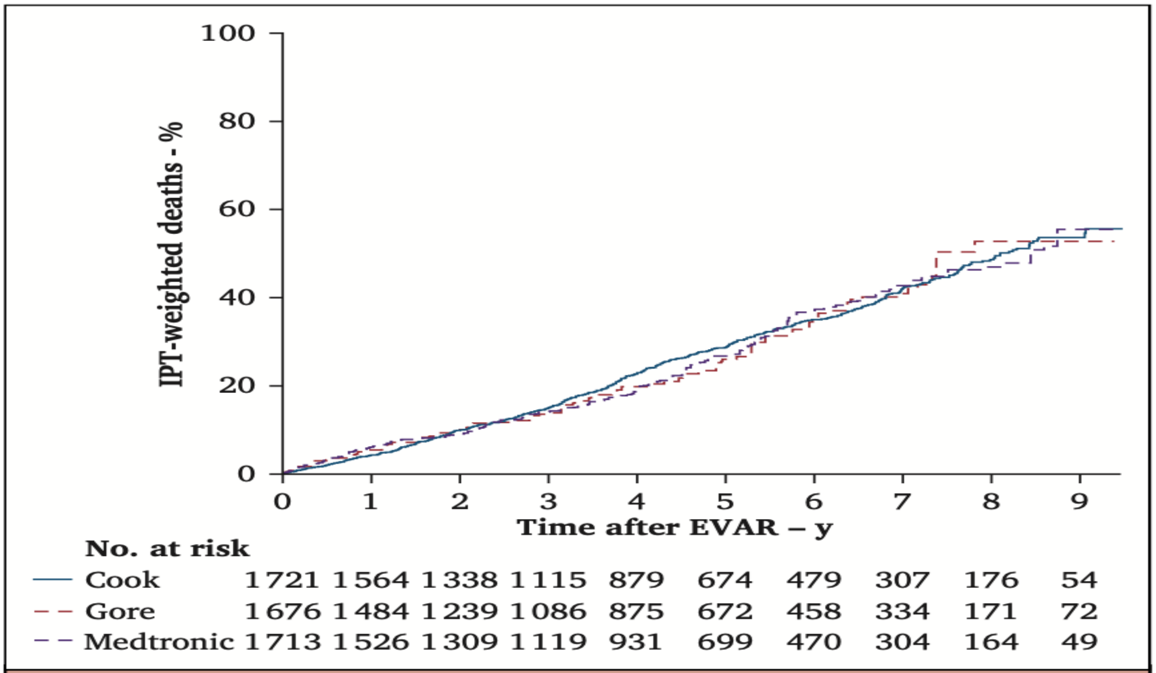

Figure 2. Inverse probability of treatment (IPT) weighted cumulative Kaplan—Meier estimate of graft failure and number of patients at risk for all cause mortality following endovascular aneurysm repair (EVAR) of intact abdominal aortic aneurysm using standard Cook, Medtronic, and Gore devices.

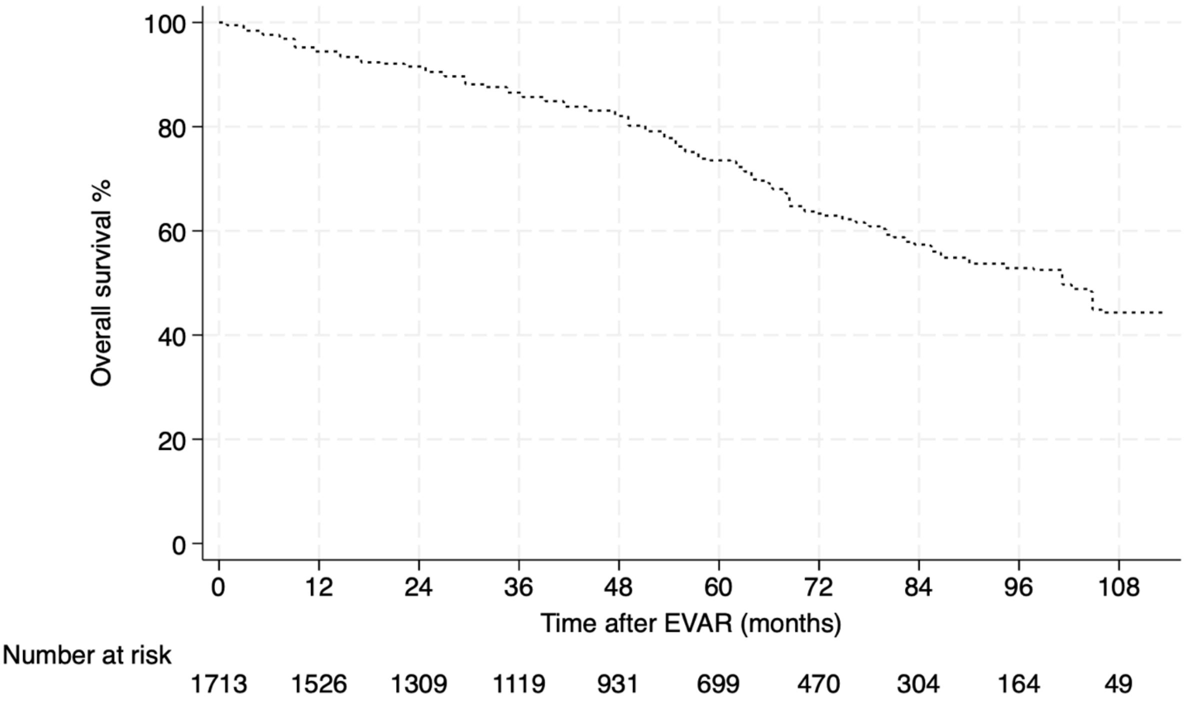

C. Original and regenerated KM of Georgiadis S.G. Et al. [43]

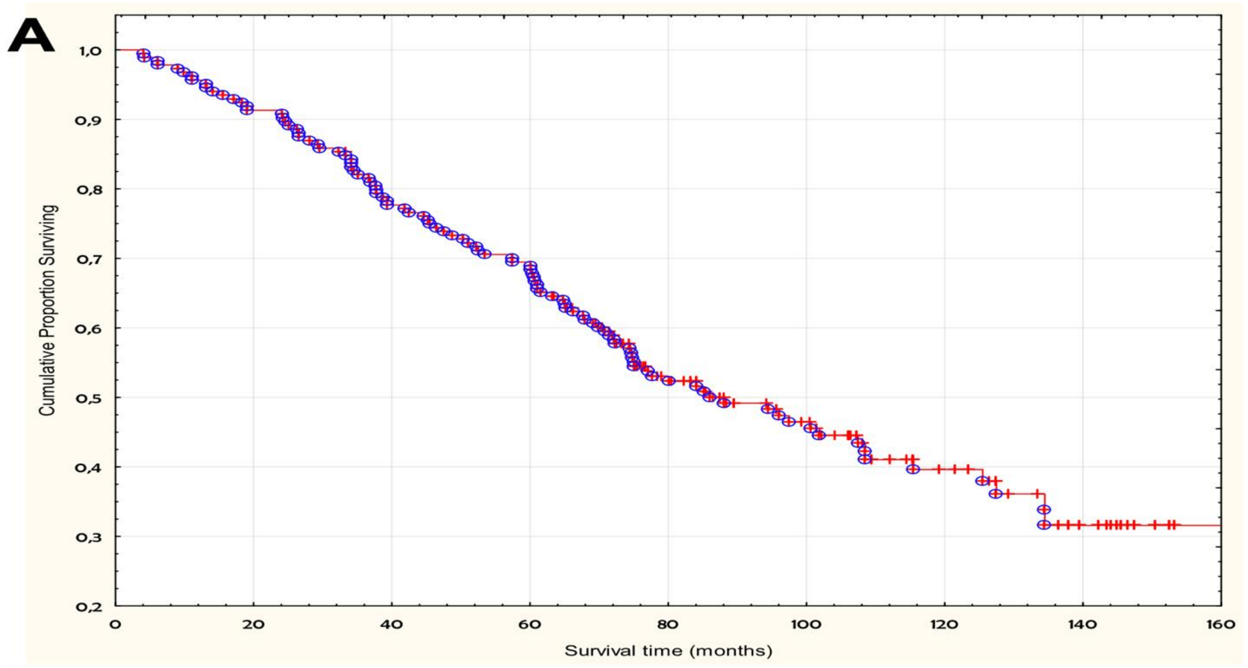

Time (months)	24	36	48	60	72	84	96	120
N at risk	168	150	135	127	99	69	53	26

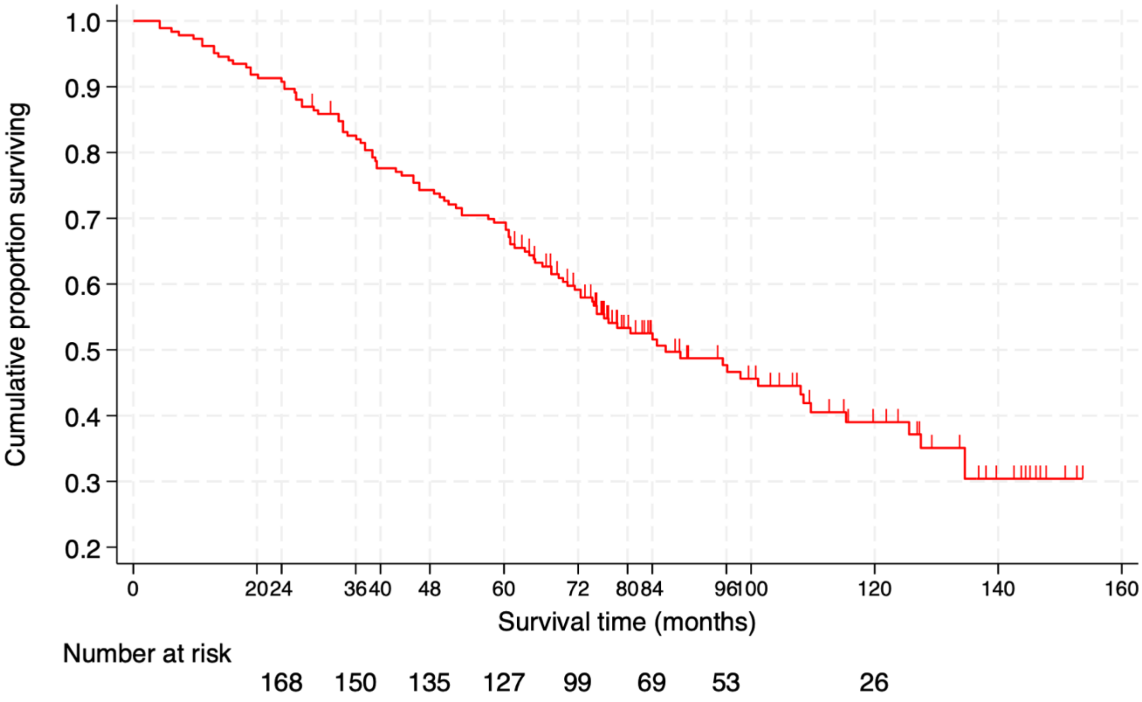

D. Original and regenerated KM of Kvinlaug K.E. et al. [31]

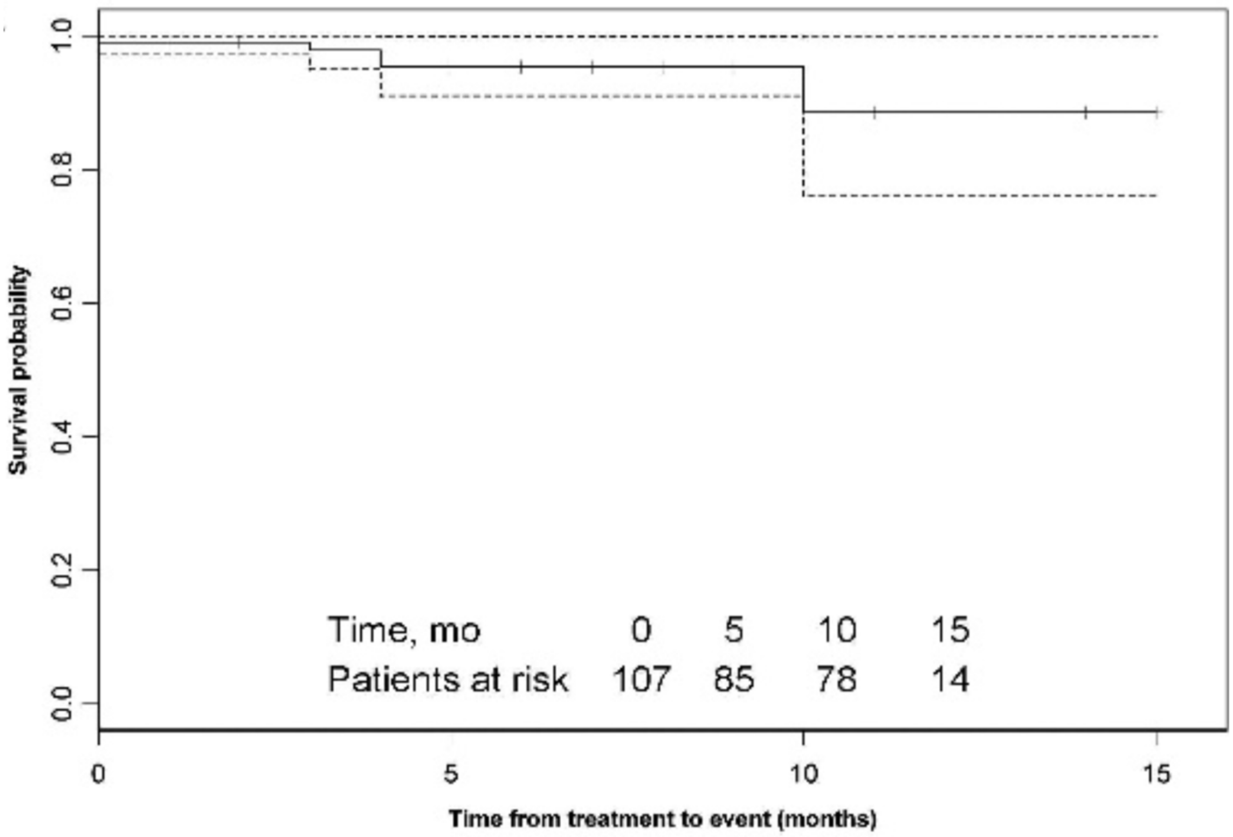

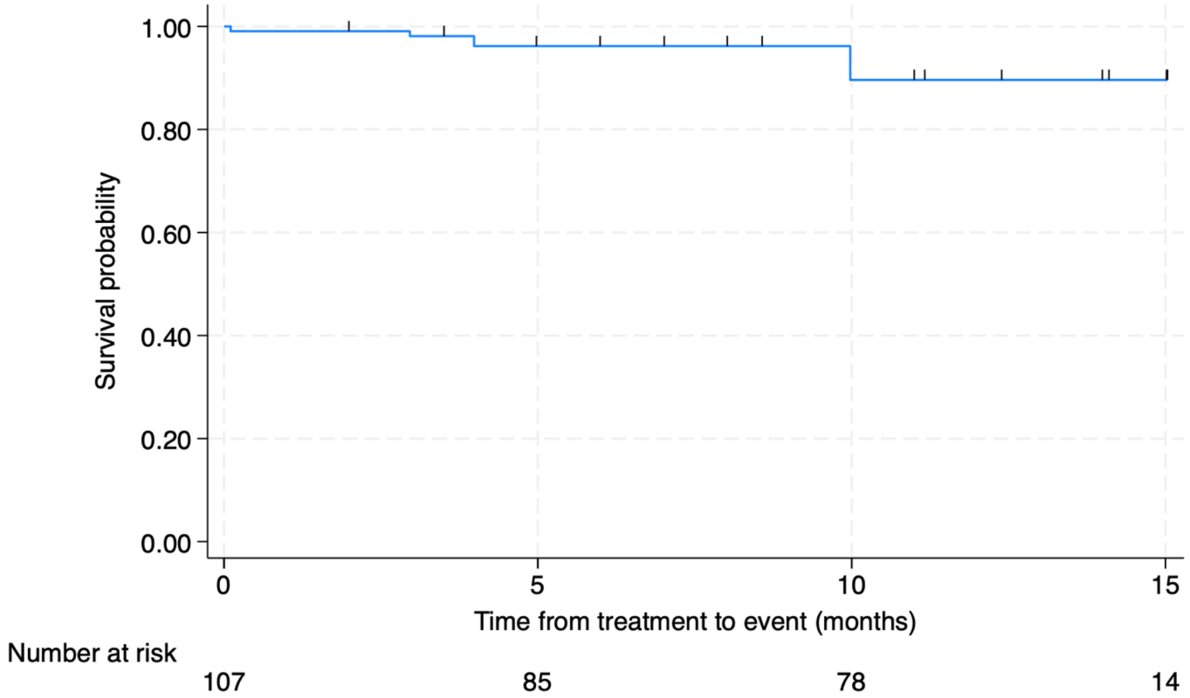

E. Original and regenerated KM of Omran S et al. [34]

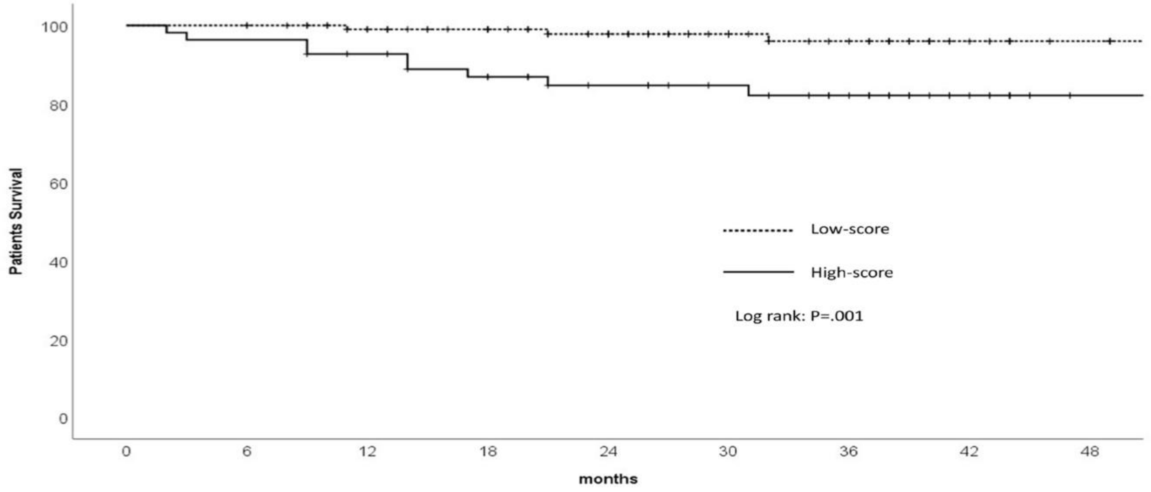

	Follow-up	0	6	12	18	24	30	36	42	48
Low-score	Pt at risk	110	110	100	90	76	59	48	35	28
	SE	.00	.01	.01	.02	.02	.02	.02	.02	.02
High-score	Pt at risk	55	53	49	44	37	33	28	19	12
	SE	.03	.04	.05	.05	.05	.06	.06	.06	.13

Figure 3. Kaplan-Meier survival estimates of low-score and high-score patients (Kaplan-Meier log-rank test, p=0.001). SE, standard error.

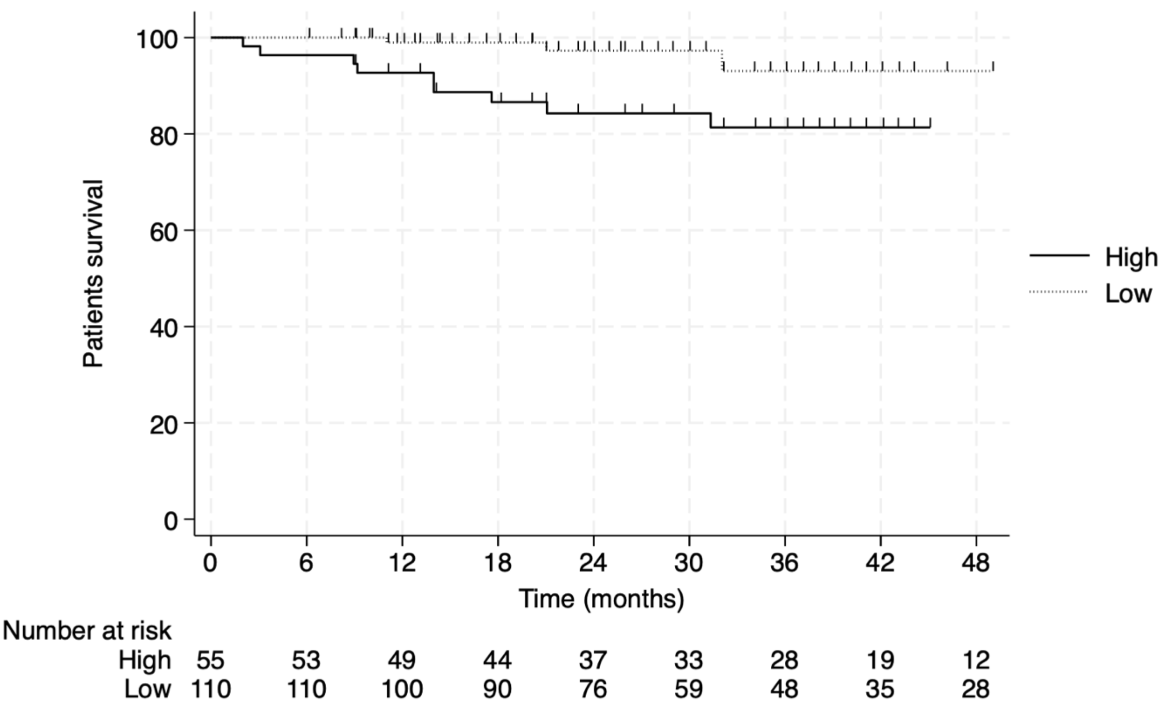

Supplemental Figure S6

A. Regenerated KM of Özdemir-van Brunschot D.M.D. et al. [35] (The extracted data were used to reconstruct a single final KM)

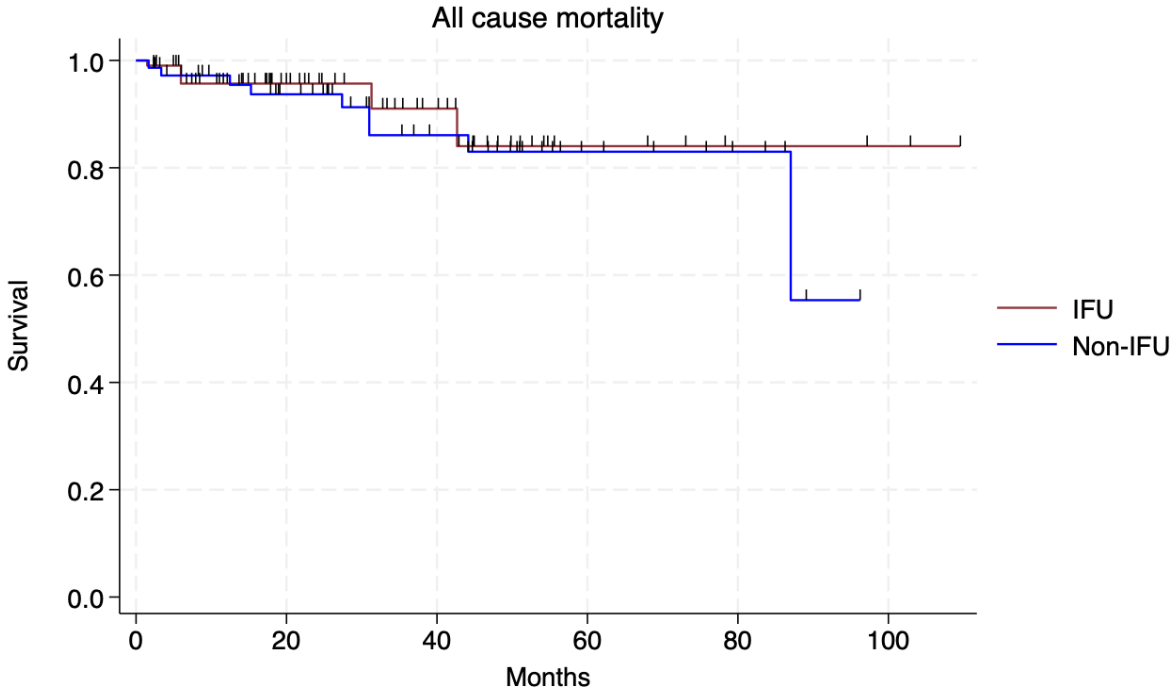

B. Original and regenerated KM of Pecoraro F. et al. [45]

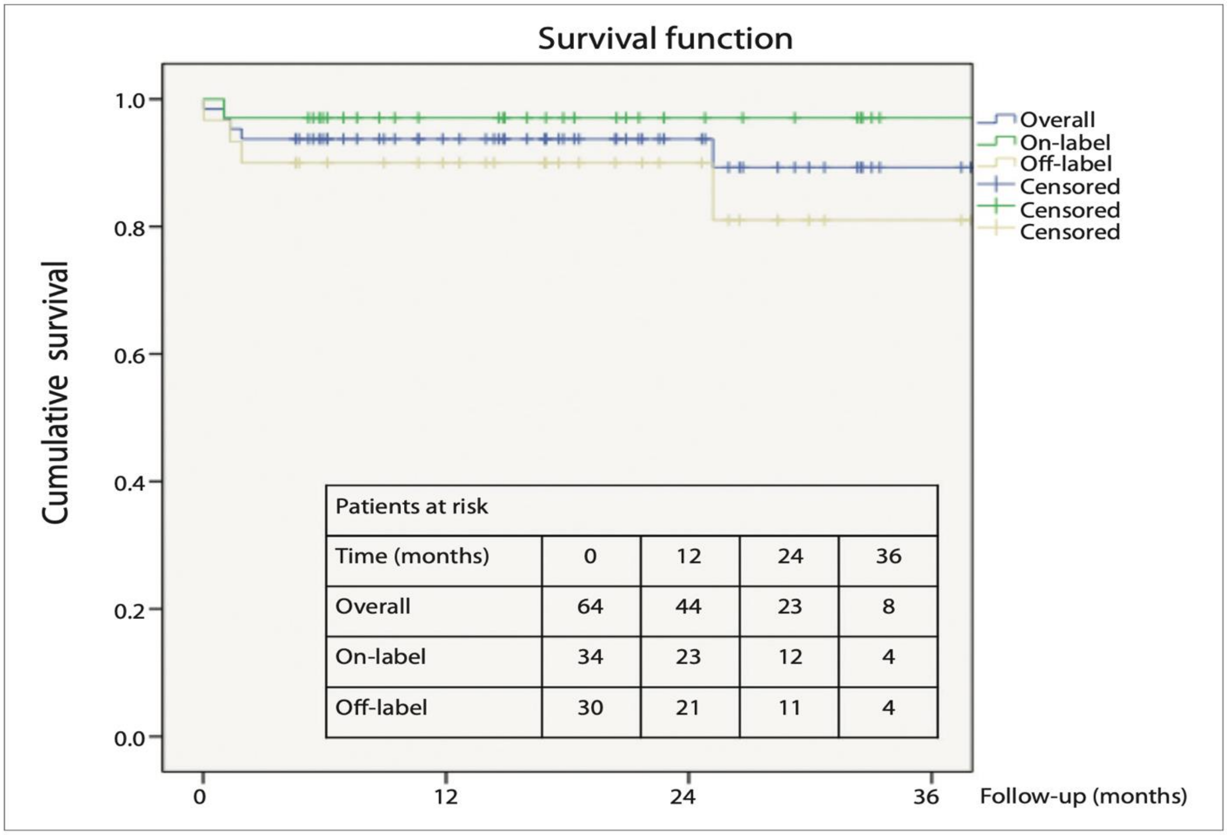

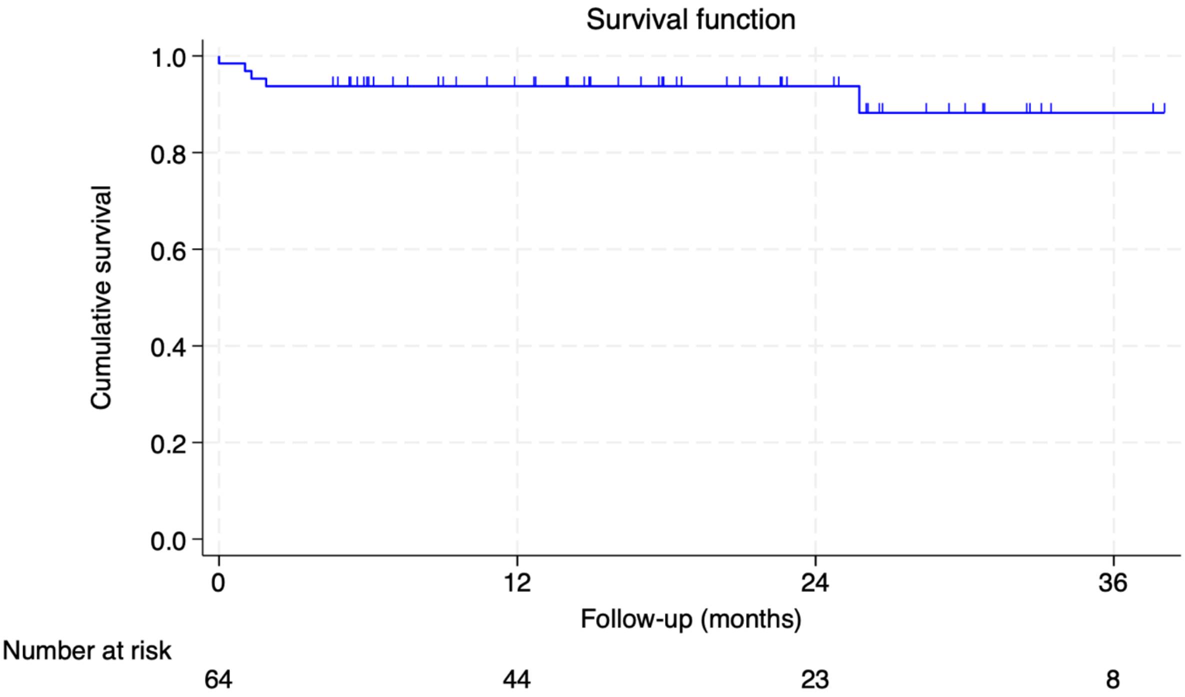

C. Original and regenerated KM of Rouwet E.V. et al. [28]

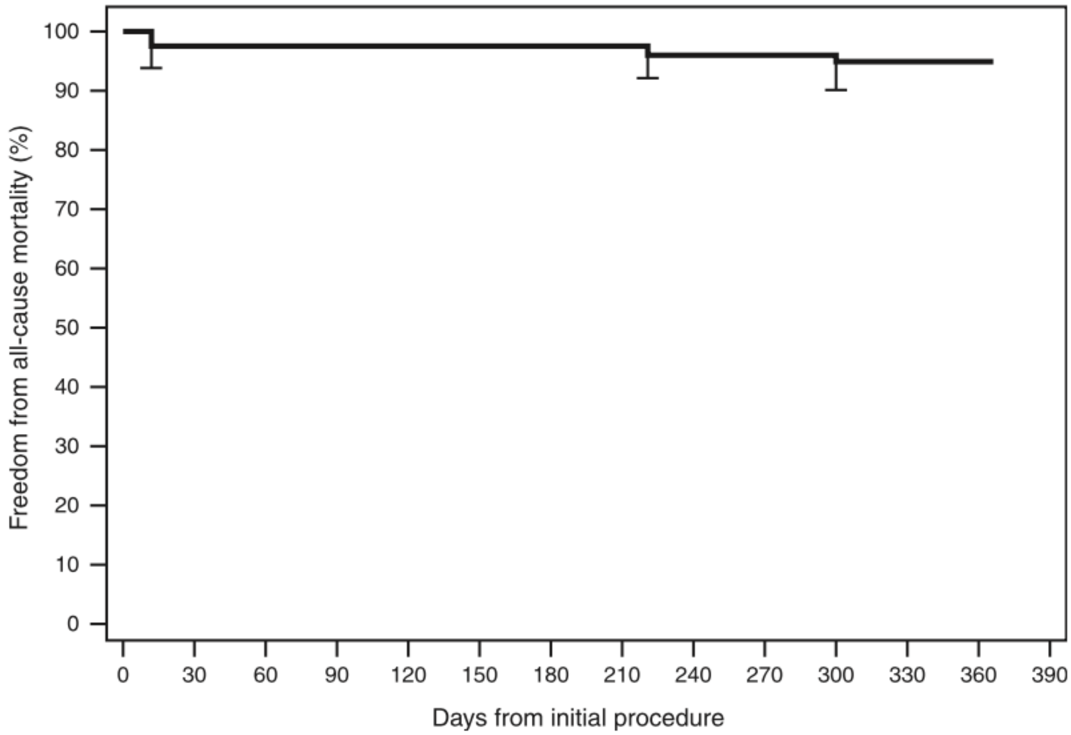

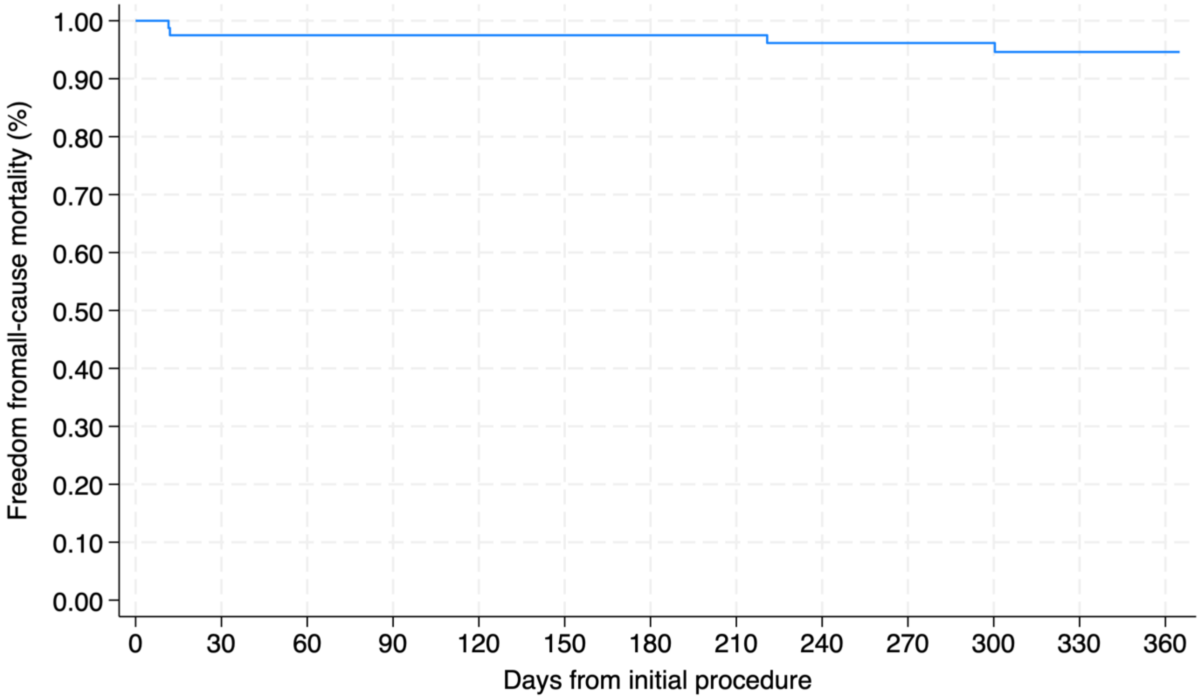

D. Regenerated KM of Salemans P.B. et al. [10]

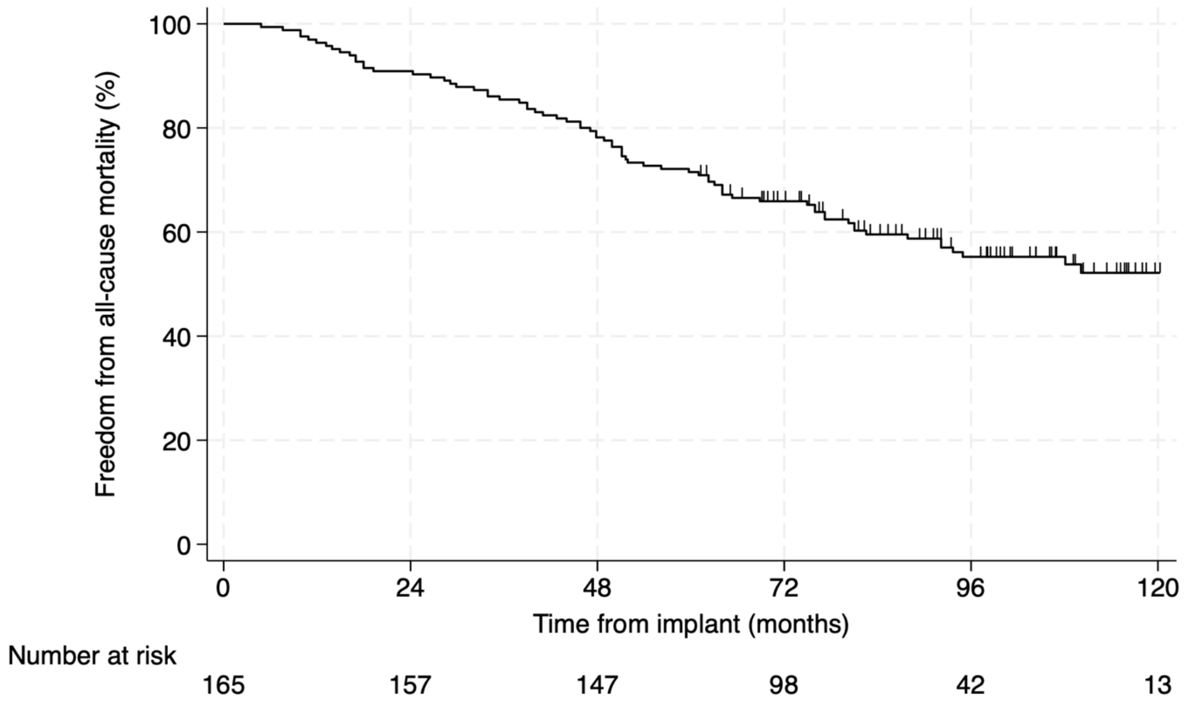

E. Regenerated KM of Sekimoto Y. et al. [39]

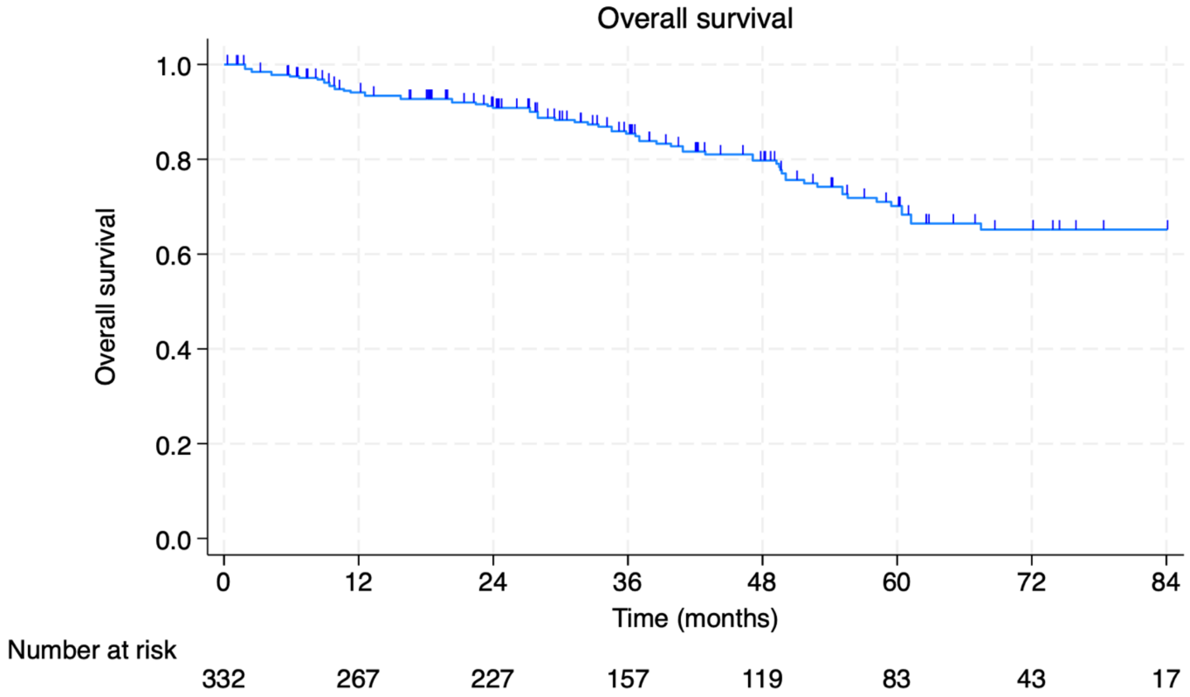

Supplemental Figure S7

A. Regenerated KM of Setacci F. et al. [11]

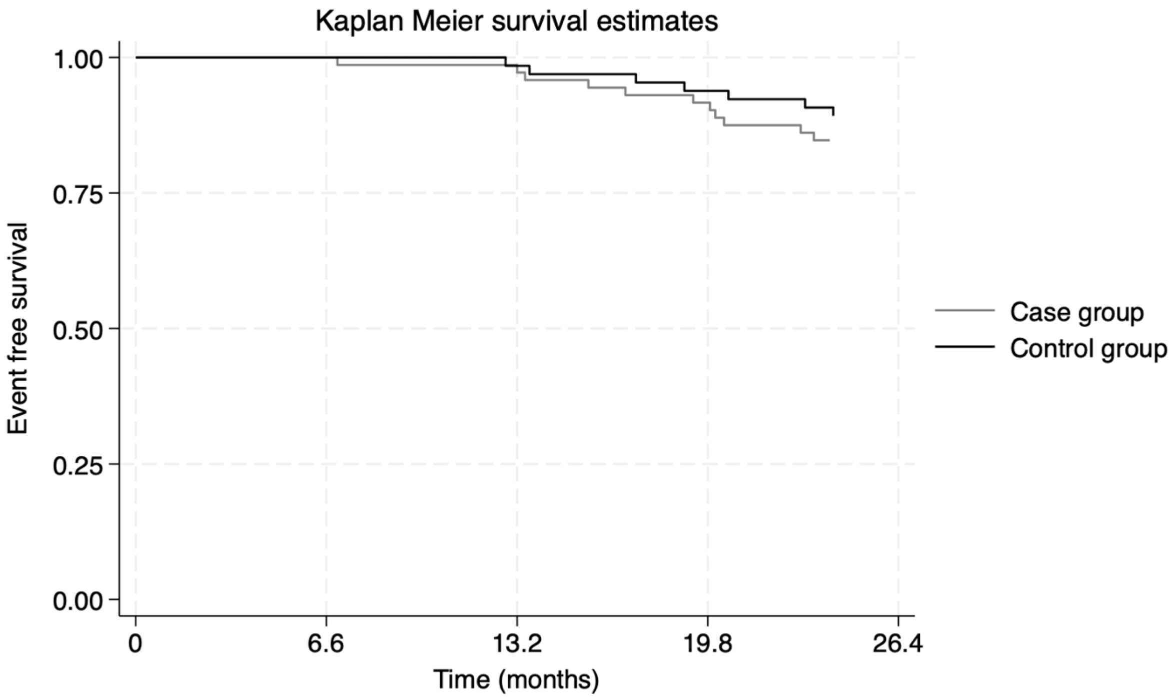

B. Original and regenerated KM of Singh M.J. et al. [44]

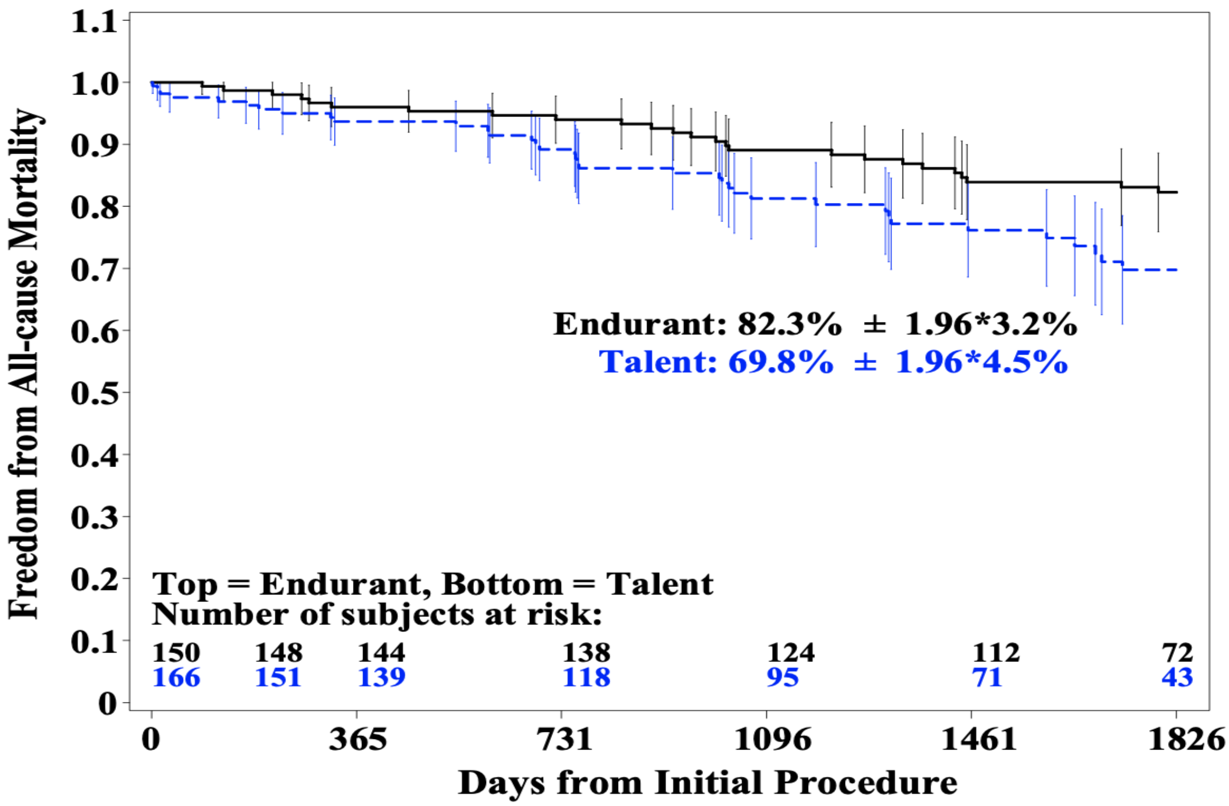

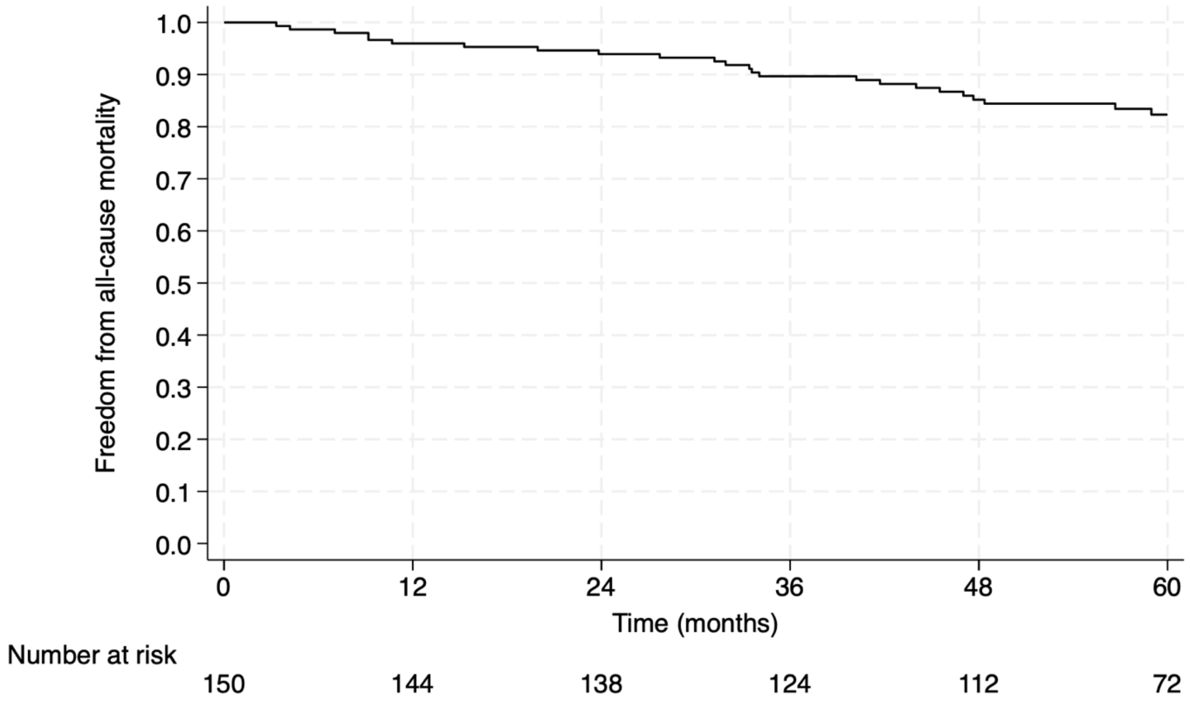

C. Original and regenerated KM of Spanos K. et al. [42]

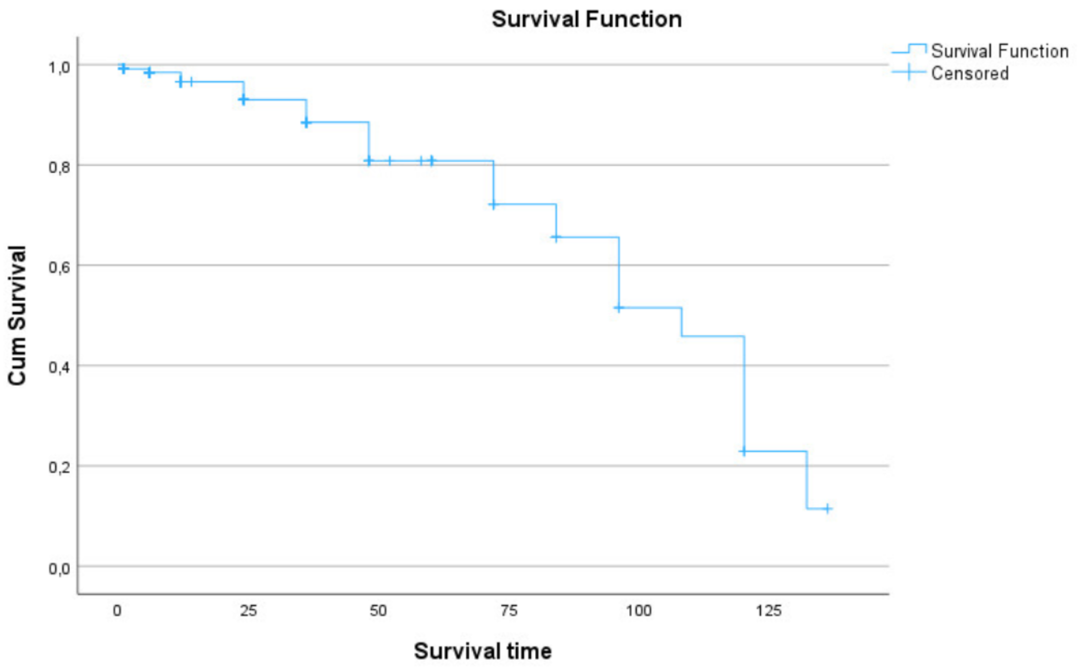

Months	12	24	36	48	60	72	84	96	108
Patients at risk	271	80	61	45	34	27	21	13	8
Events	9	12	15	19	19	22	24	27	28
Percentages	7%	93%	89%	81%	-	72.2%	66%	52%	46%
SE	1%	2.3%	3.4%	4.8%	-	6.4%	7.3%	9.2%	9.8%

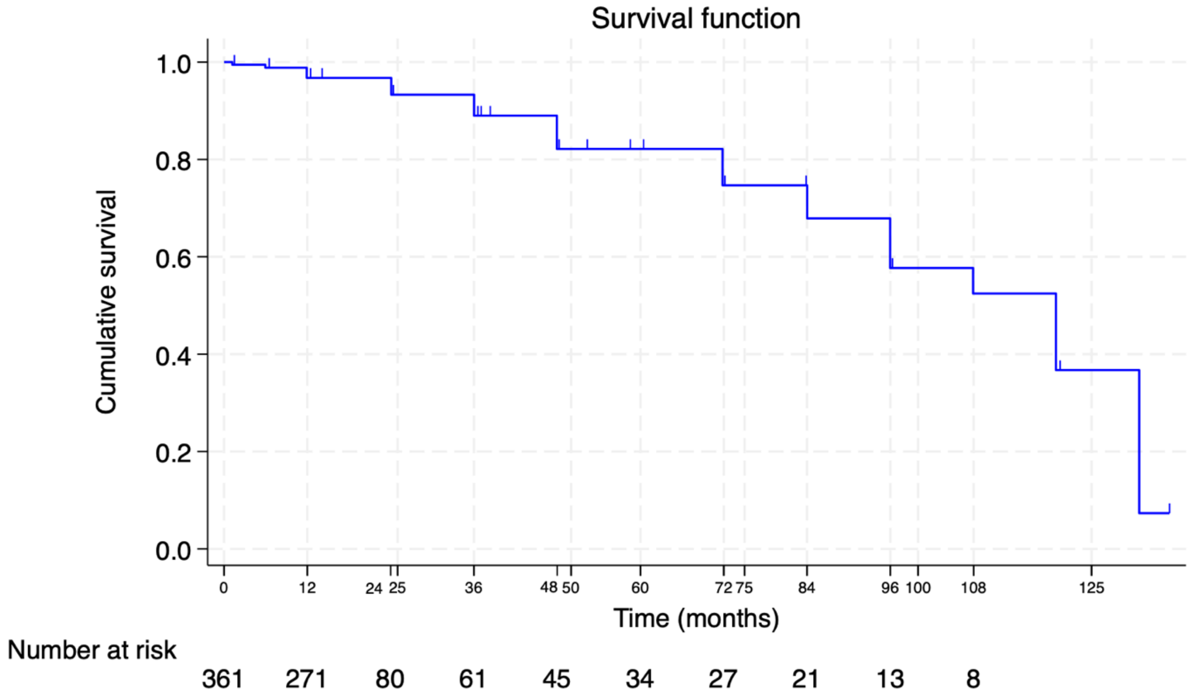

D. Original and regenerated KM of Vedani S.M. et al. [40]

(The extracted data were used to reconstruct a single final KM)

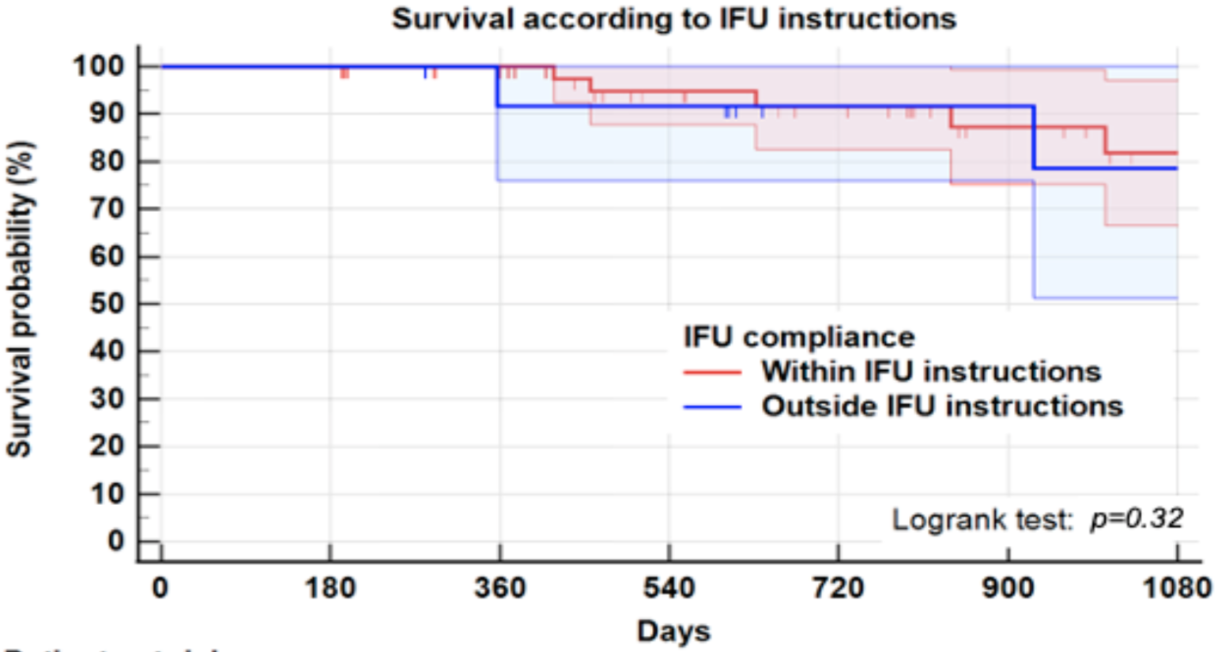

Patients at risk

Group: Within IFU instructions	47	47	42	32	27	18	13
Group: Outside IFU instructions	13	13	11	11	7	7	6

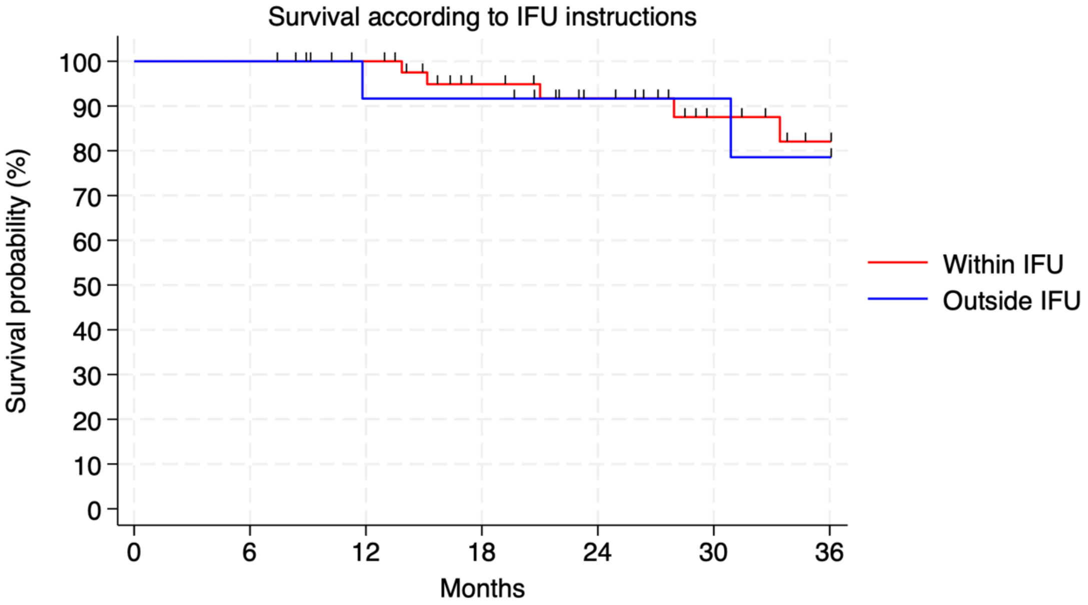

Number at risk							
Within IFU	47	47	42	32	27	18	13
Outside IFU	13	13	11	11	7	7	6
