# Supplementary material for: Endurant Stents in Abdominal Aortic Aneurysm Repair: A Systematic Review and Meta-Analysis
Source: J Clin Med. 2025 Sep 12;14(18):6453. doi: 10.3390/jcm14186453 (PMC12470529; doi:10.3390/jcm14186453)

Kaplan–Meier (KM) curves of overall freedom from reintervention

Supplemental Figure S8

A. Original and regenerated KM of Mannetje Y.W. et al. [46]

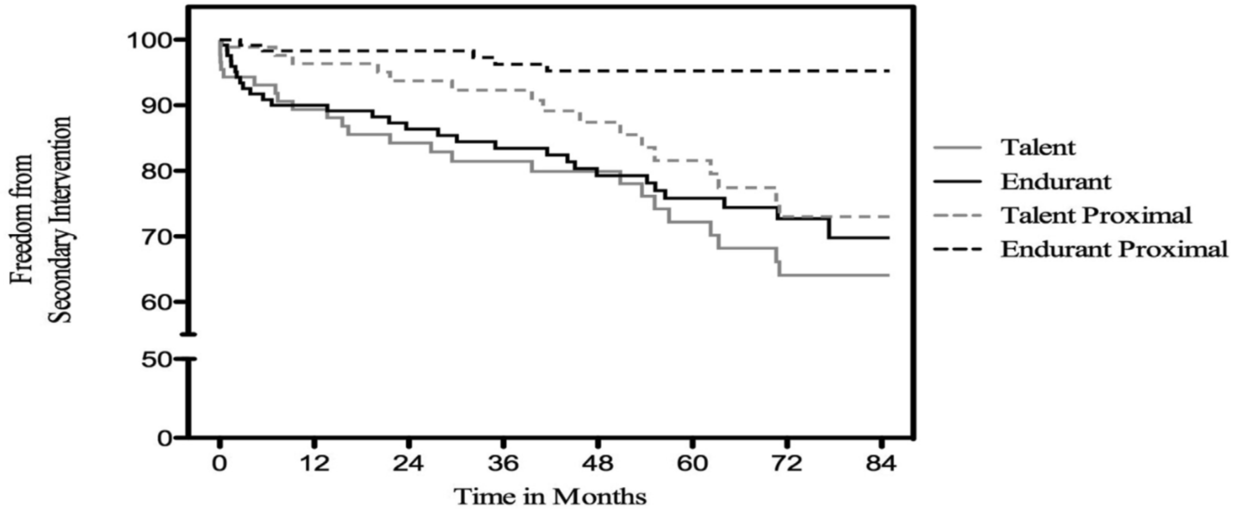

	Day 0	30-days	1-year	5-years	7-years
Talent overall Patients at risk (SE)	88	81 (.025)	72 (.034)	36 (.055)	31 (.062)
Endurant overall Patients at risk (SE)	126	120 (.016)	104 (.028)	65 (.042)	8 (.052)

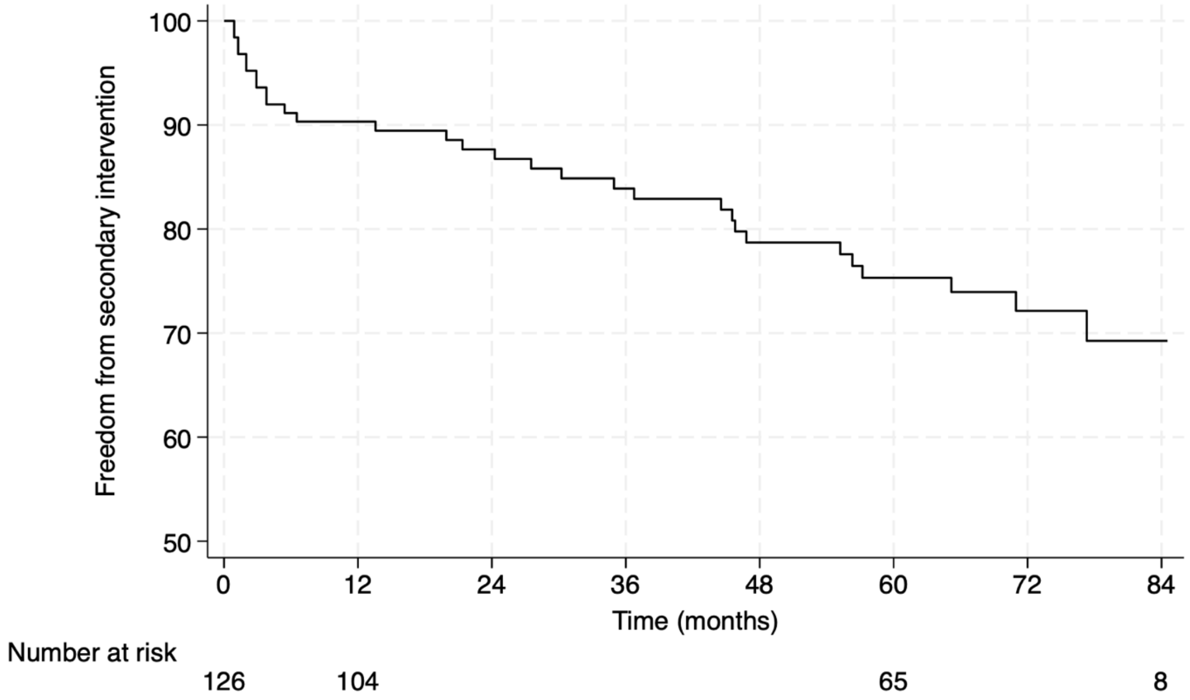

B. Original and regenerated KM of Becquemin J.P. et al. [33]

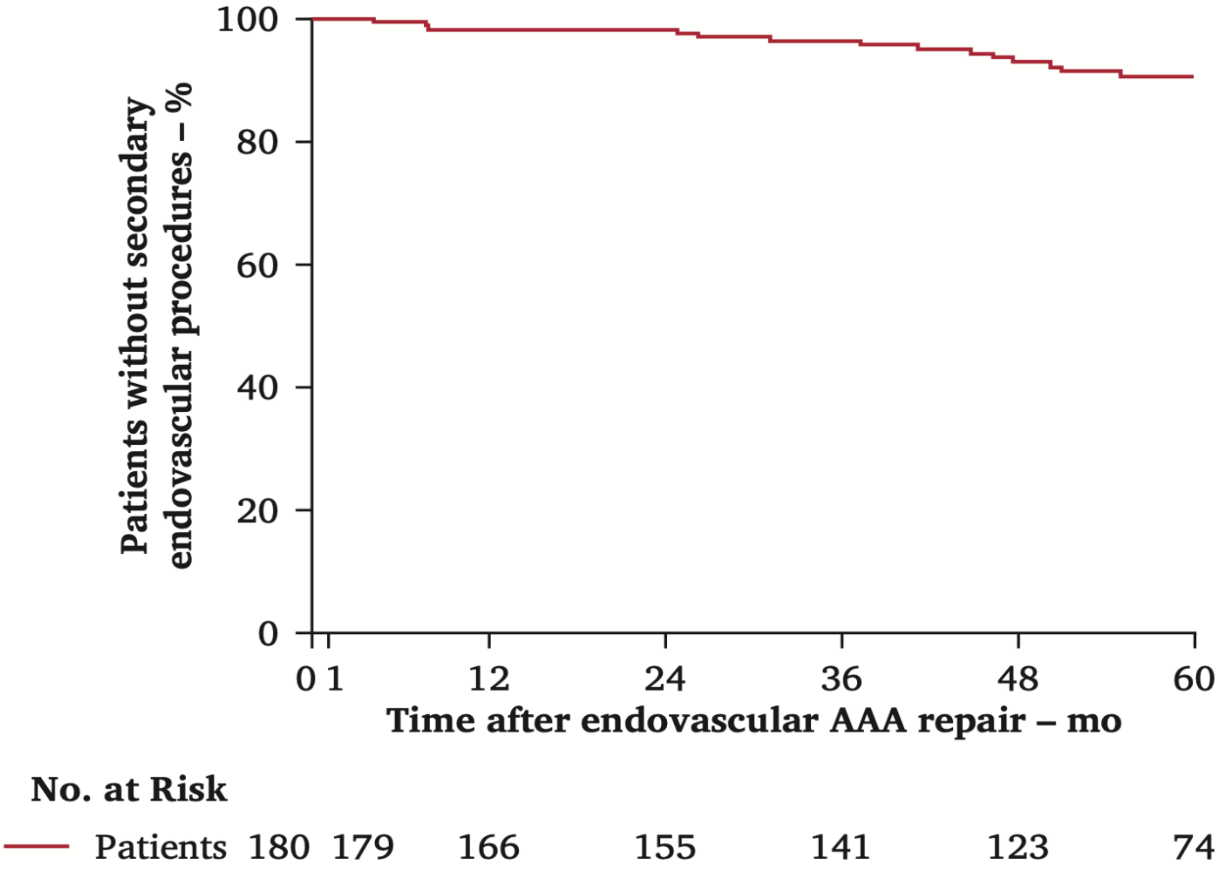

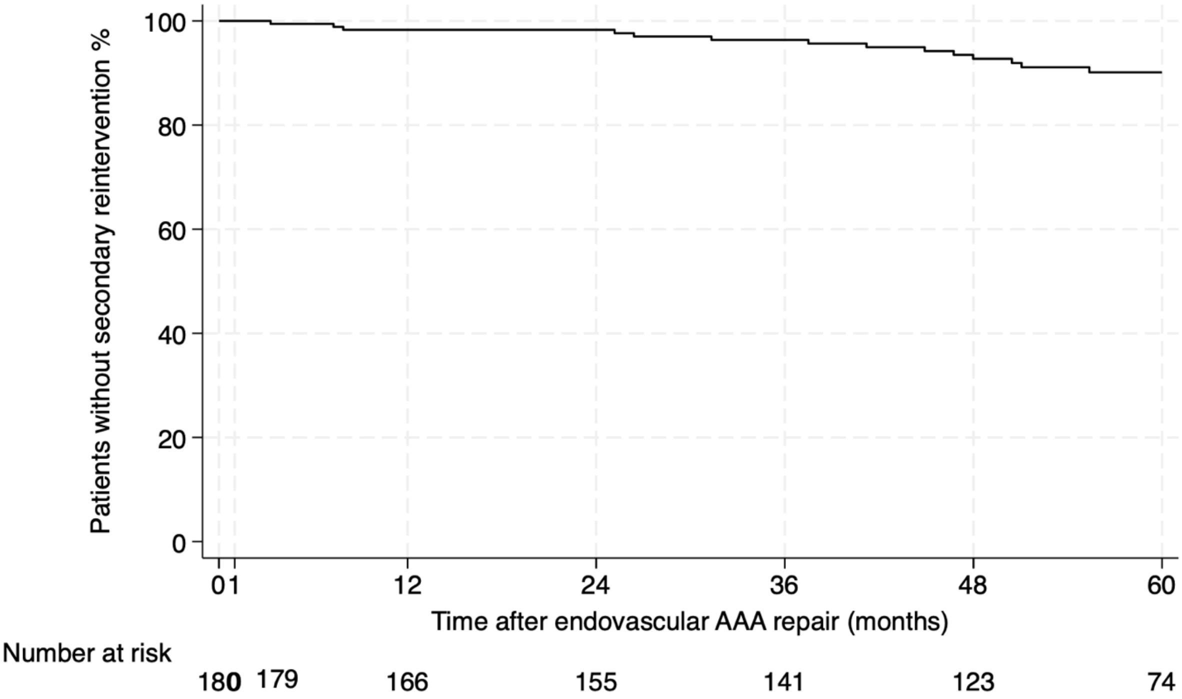

C. Original and regenerated KM of Bisdas T. et al. [36]

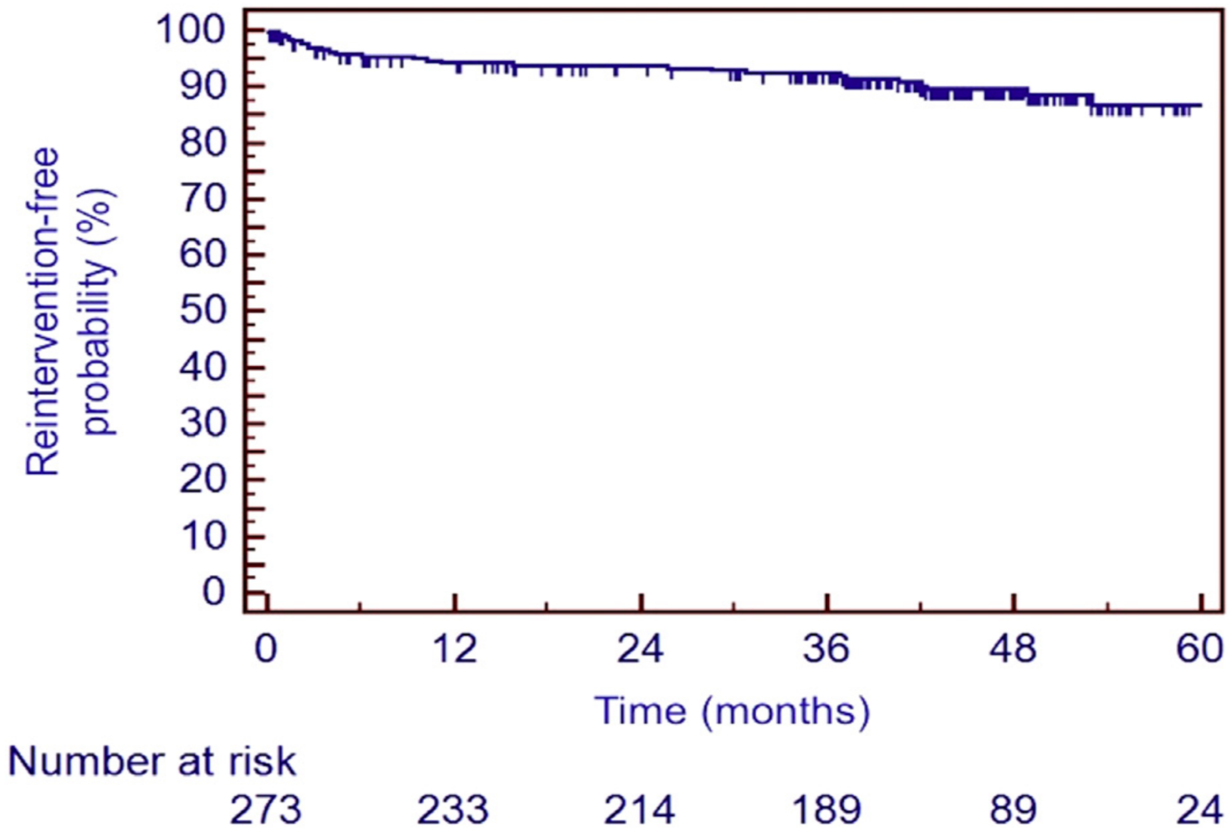

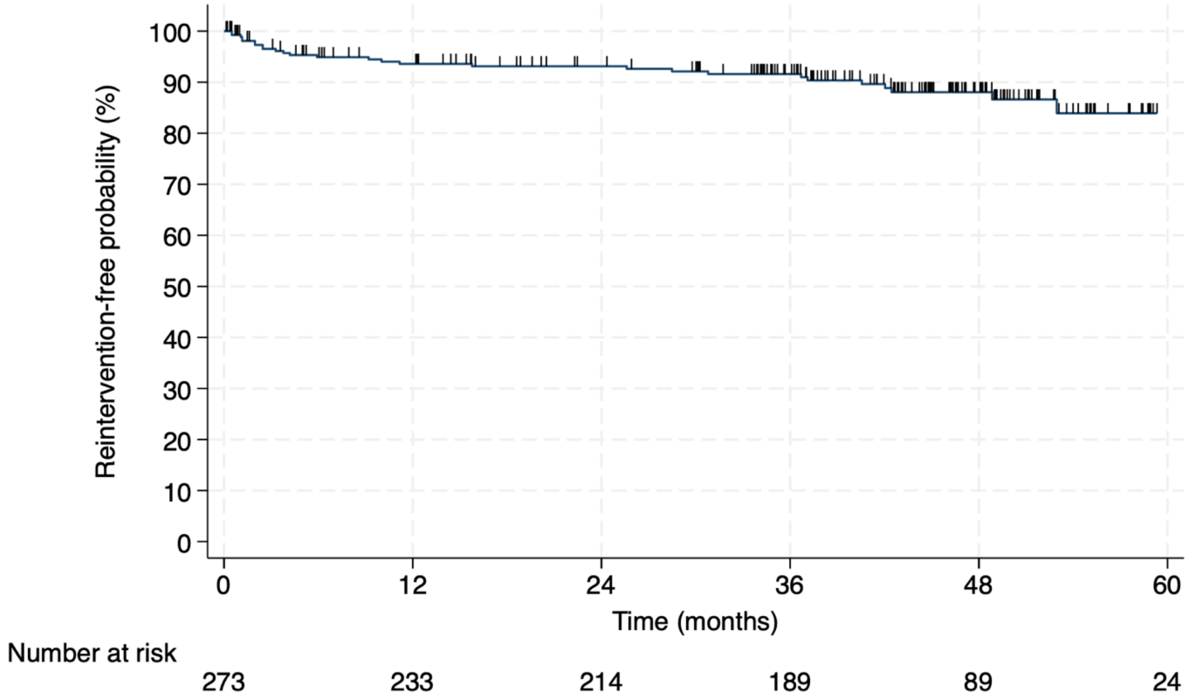

D. Original and regenerated KM of Deery S.E. et al. [38]

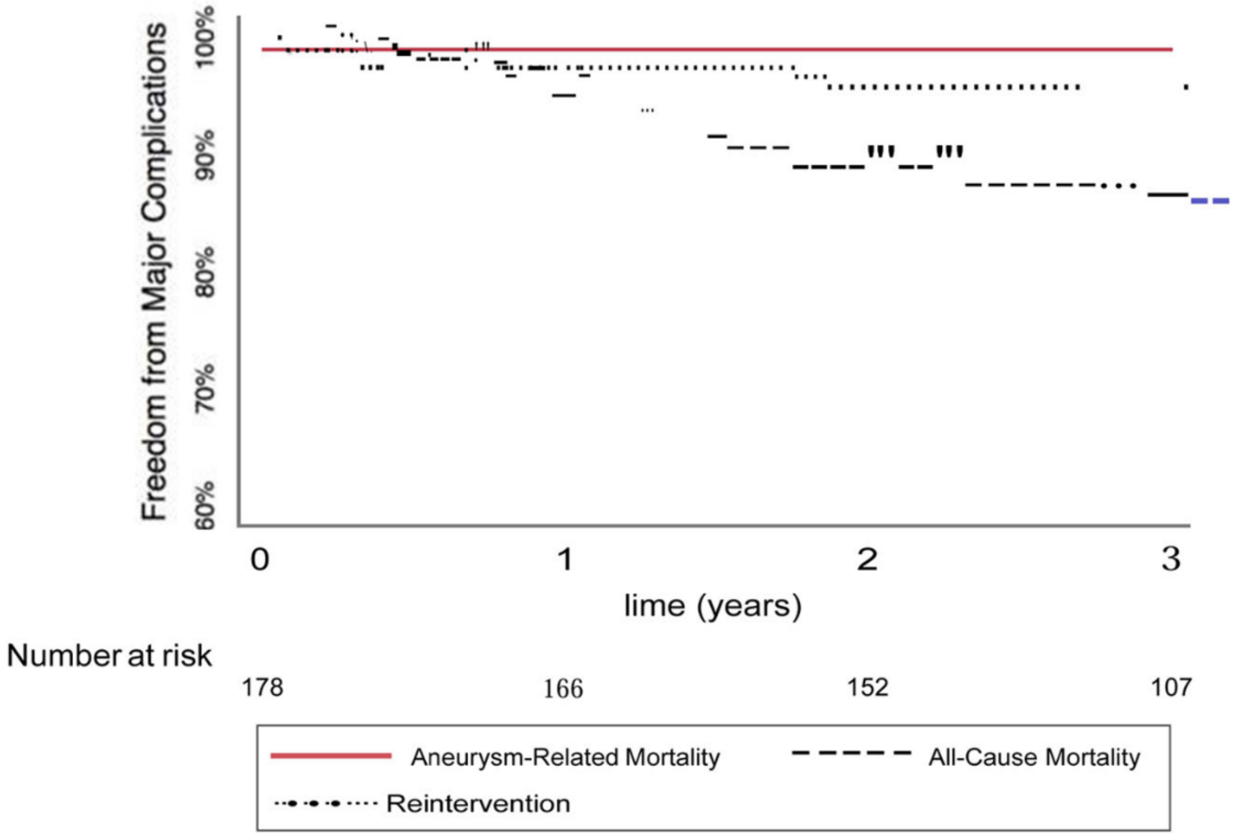

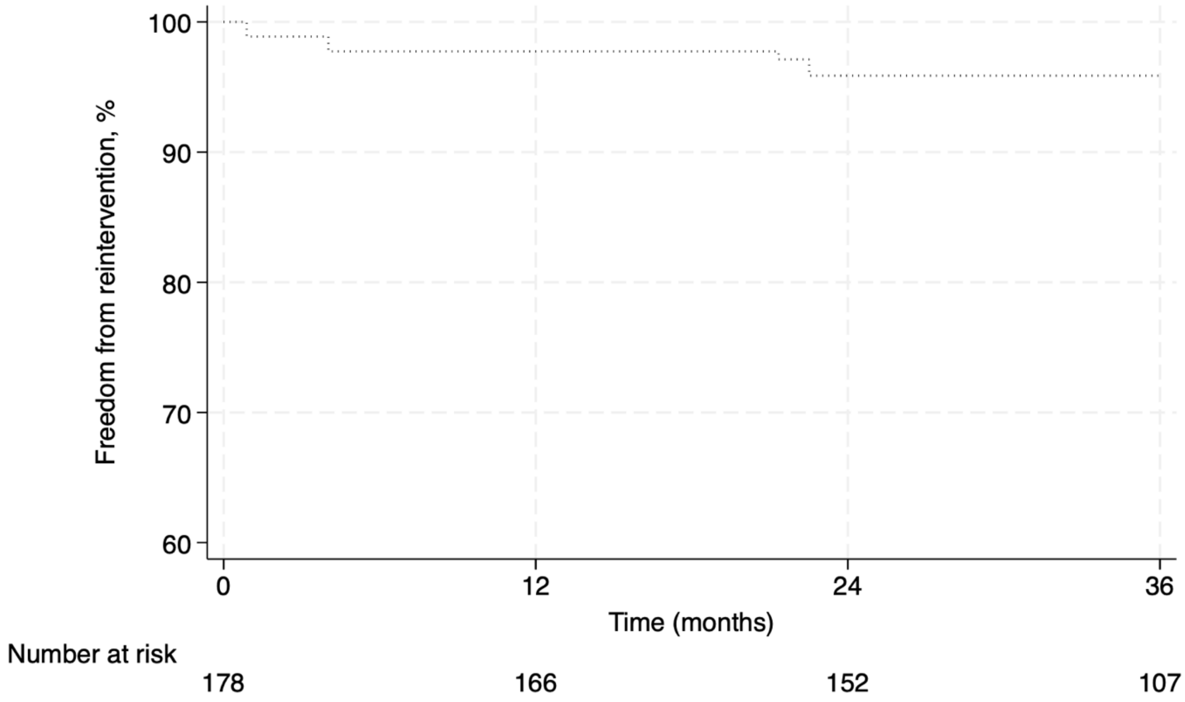

E. Original and regenerated KM of ENGAGE registry. [5]

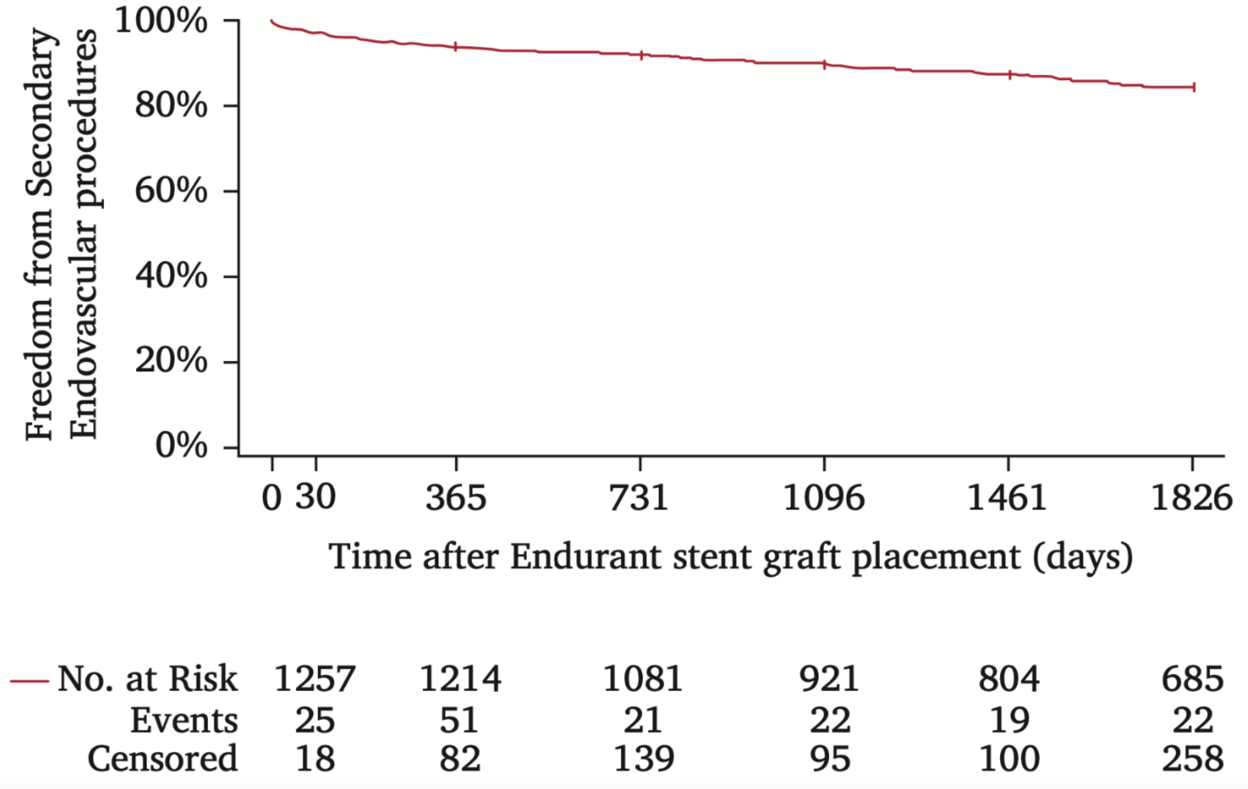

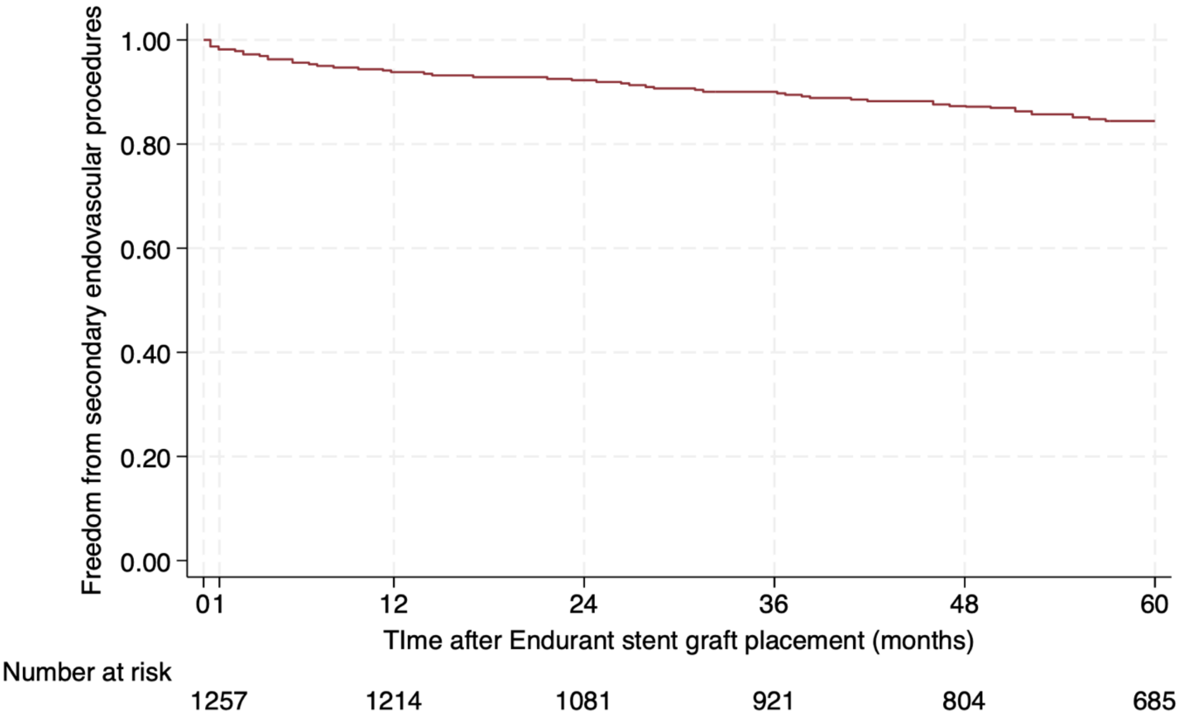

Supplemental Figure S9

A. Original and regenerated KM of Falster M.O. et al. [30]

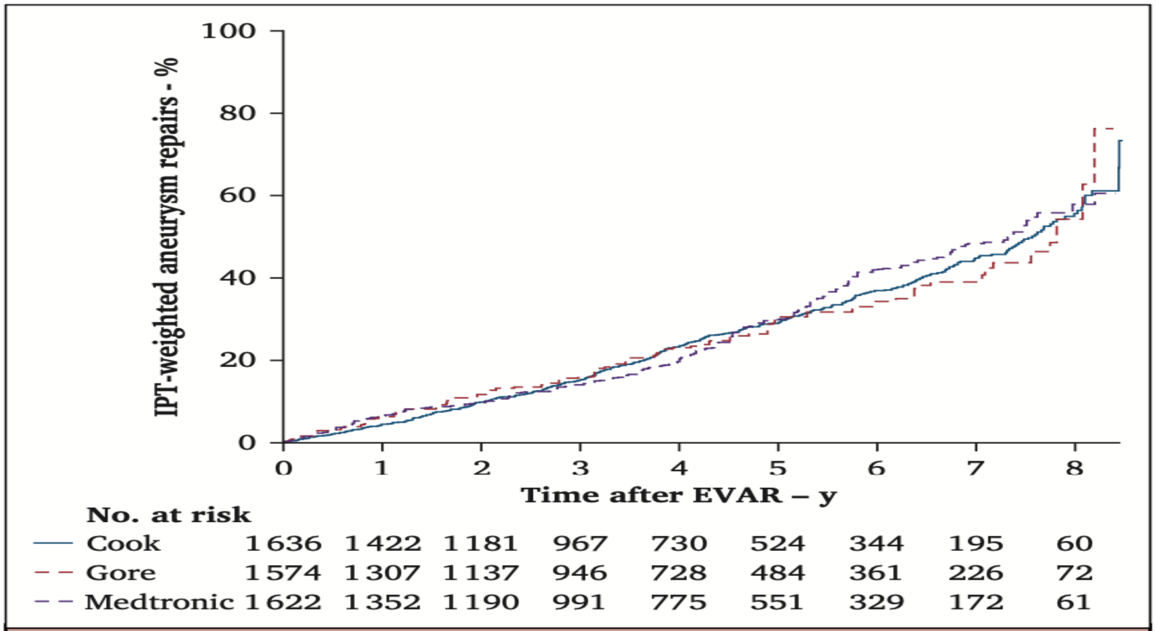

Figure 3. Inverse probability of treatment (IPT) weighted cumulative Kaplan—Meier estimate of graft failure and number of patients at risk for subsequent aneurysm repair following endovascular aneurysm repair (EVAR) of intact abdominal aortic aneurysm using standard Cook, Medtronic, and Gore devices.

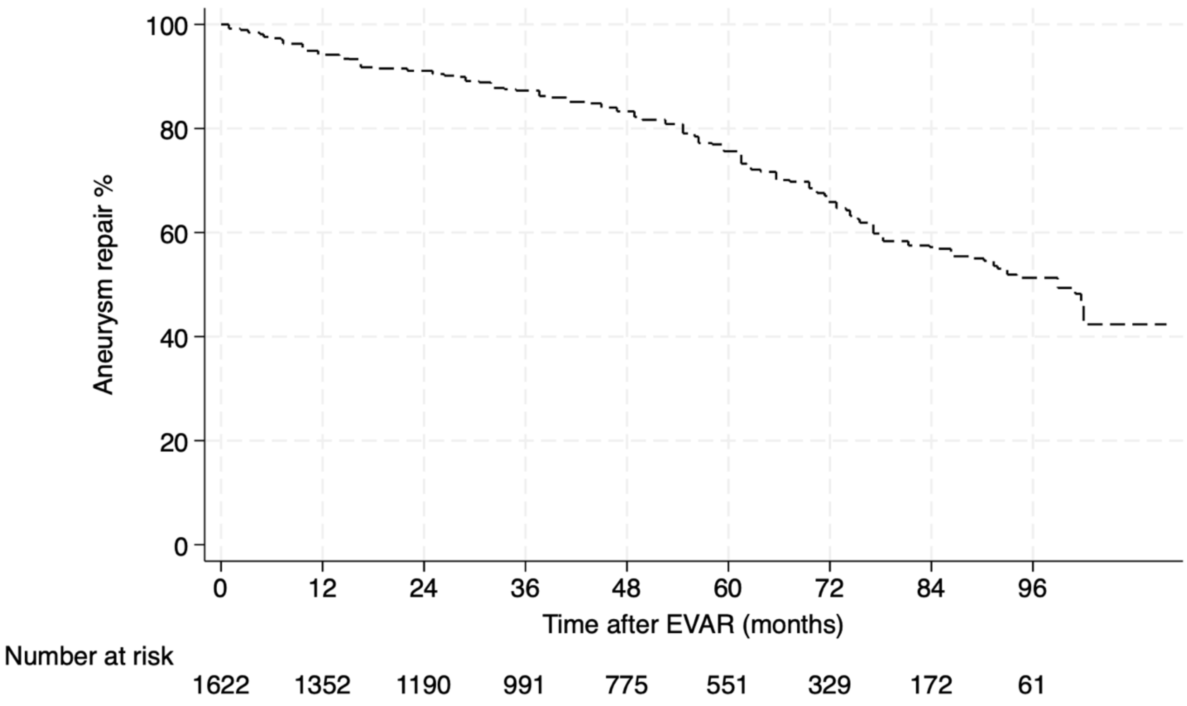

B. Original and regenerated KM of Georgiadis S.G. et al. [43]

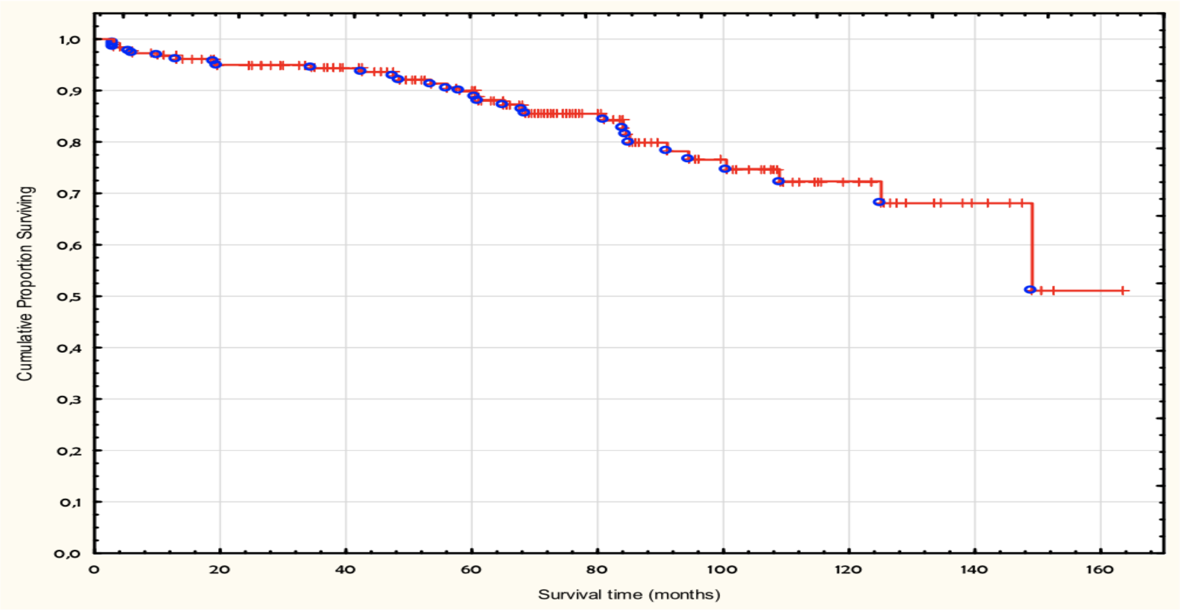

Time (months)	24	36	48	60	72	84	96	120
N at risk	161	142	126	114	85	60	45	20

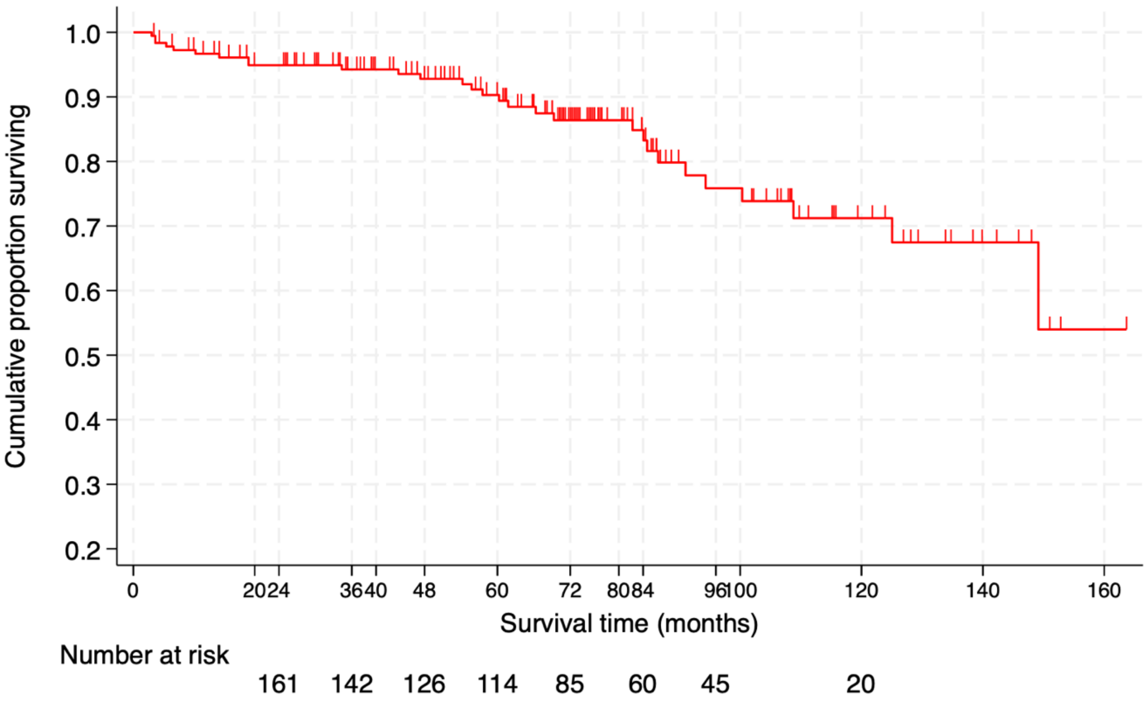

C. Regenerated KM of Kemmling S. et al. [48]

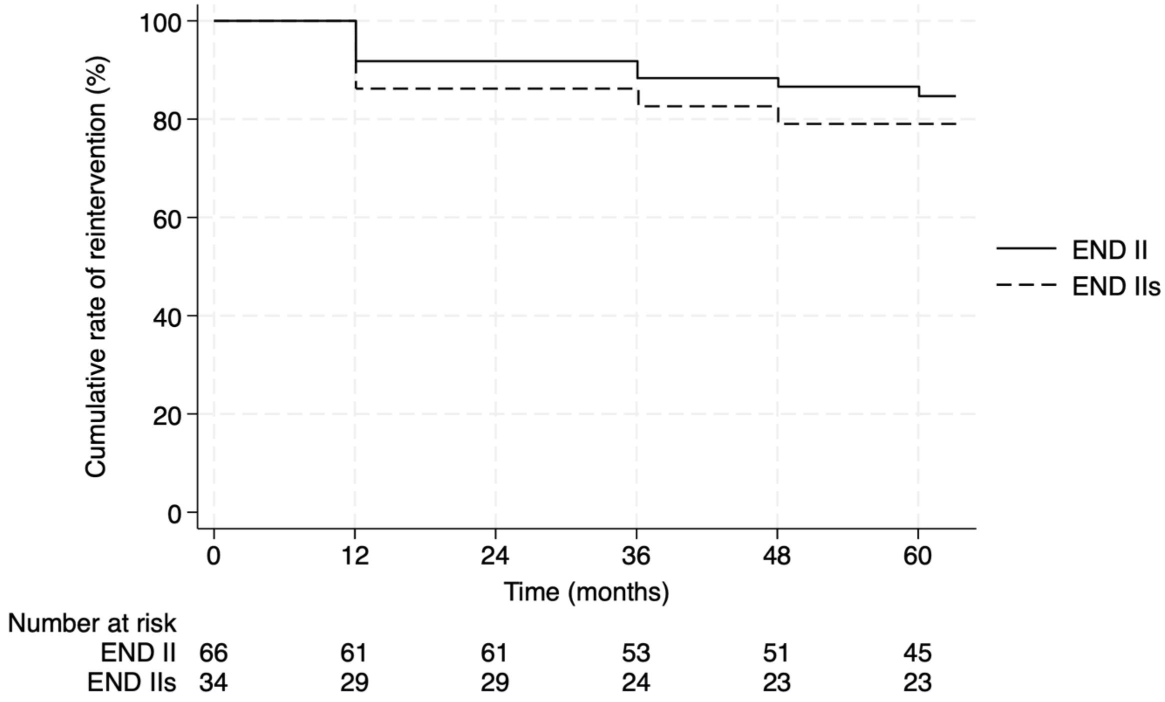

D. Original and regenerated KM of Matsagkas et al. [41]
(The extracted data were used to reconstruct a single final KM)

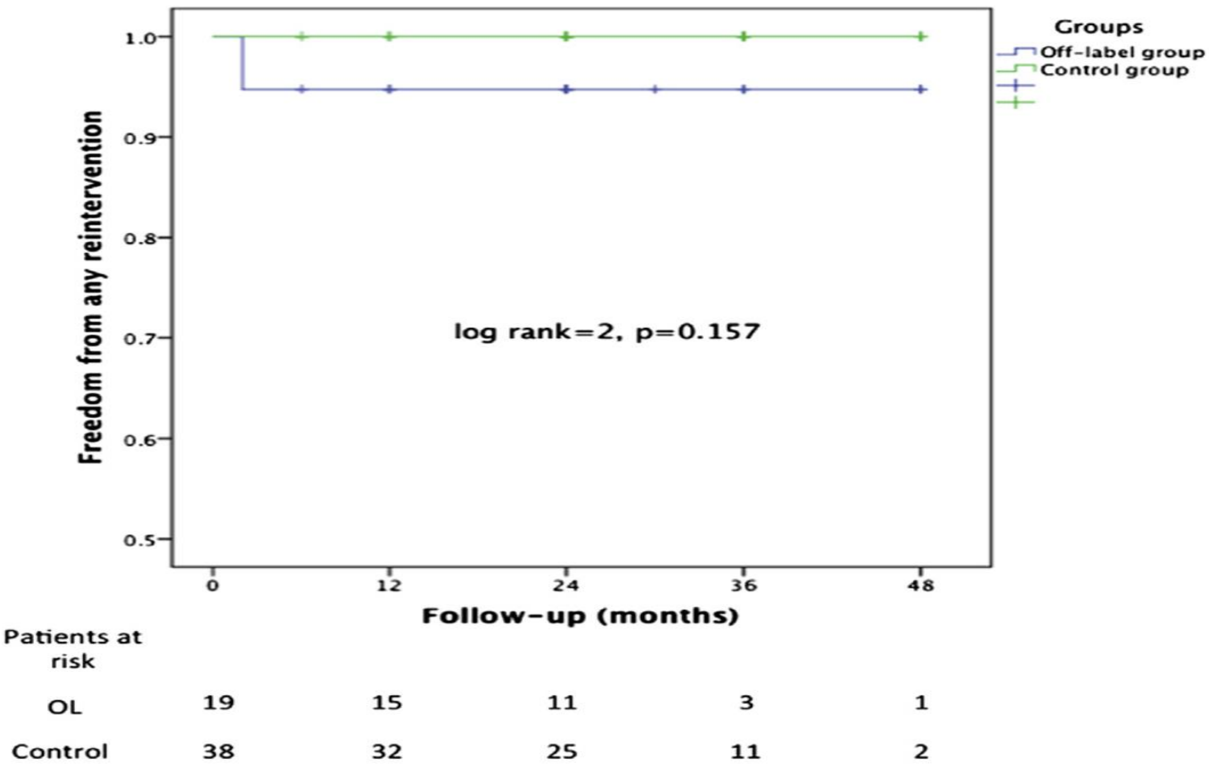

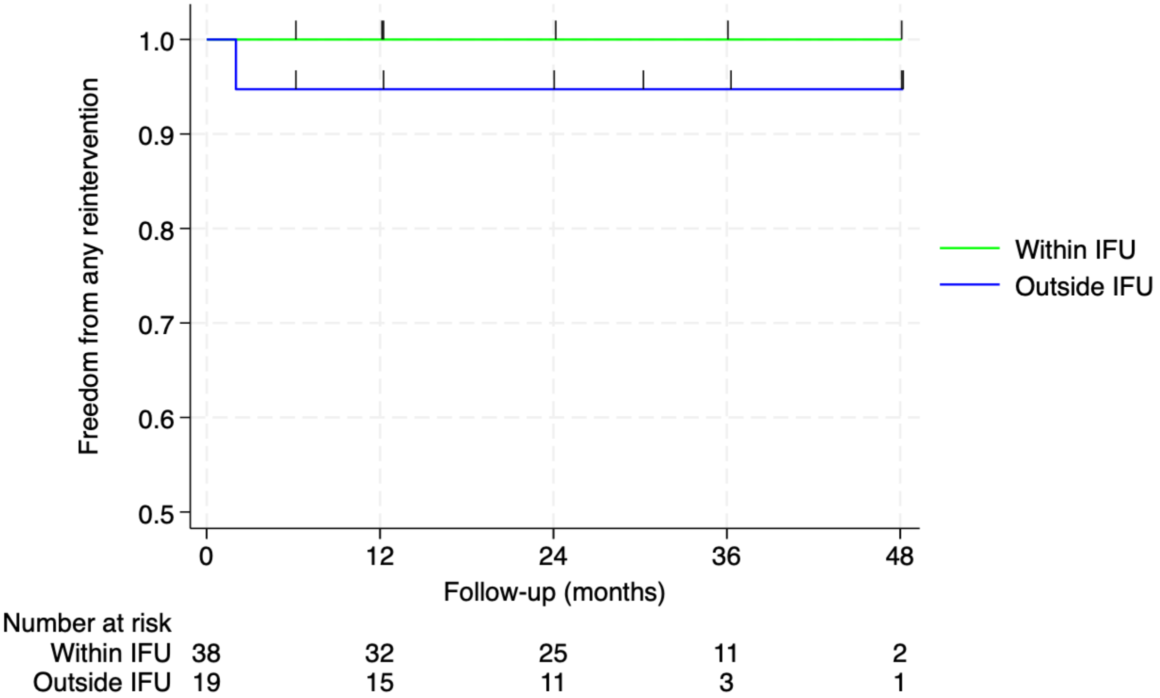

E. Original and regenerated KM of Oliveira-Pinto J. et al. [47]

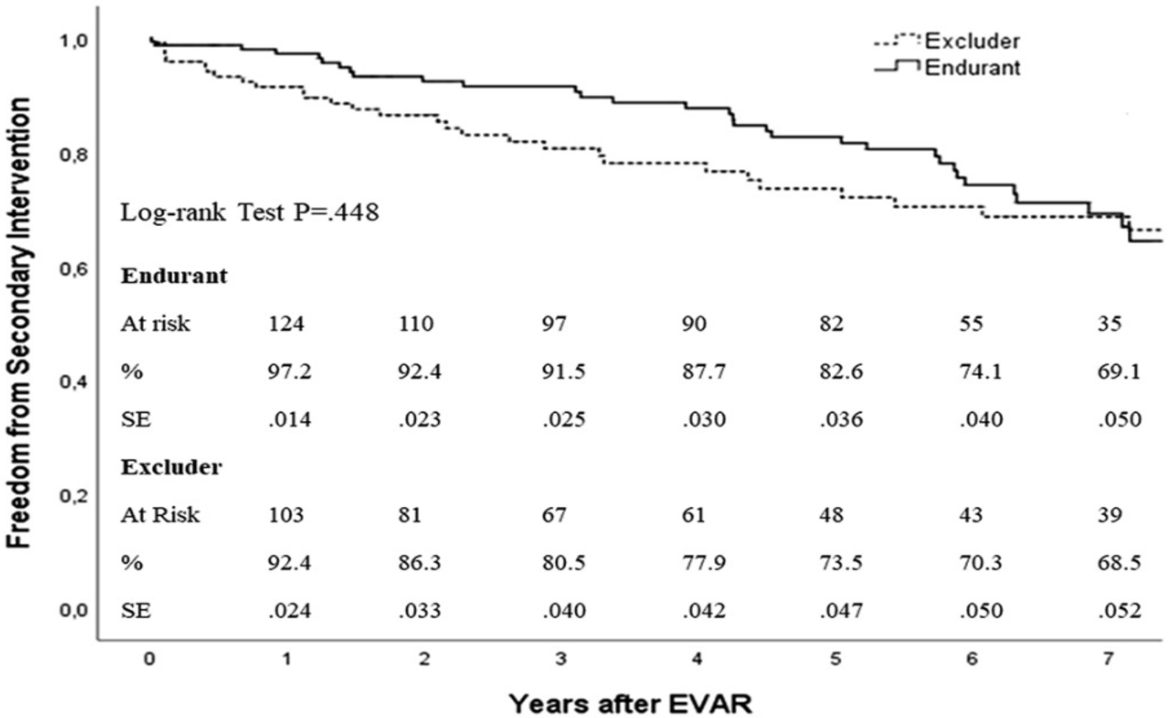

Fig 3. Kaplan-Meier curve for freedom from secondary interventions for the Endurant and Excluder stent grafts. *EVAR,* Endovascular aneurysm repair; *SE,* standard error.

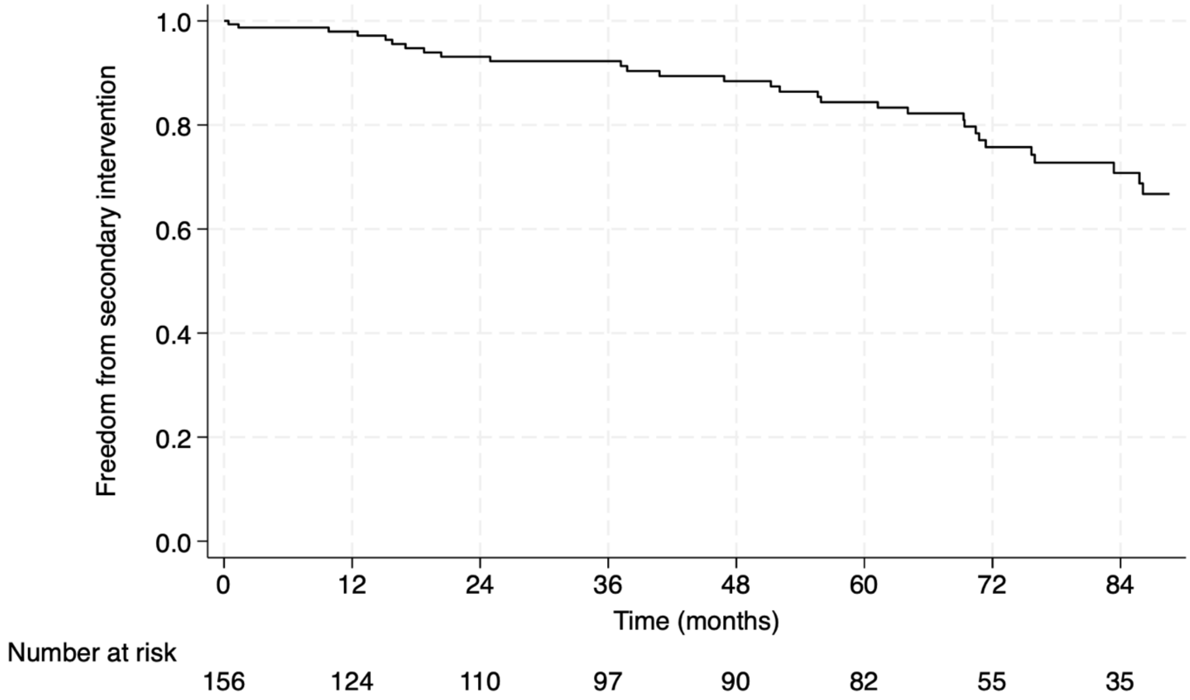

Supplemental Figure S10

A. Original and regenerated KM of Pecoraro F. et al. [45]

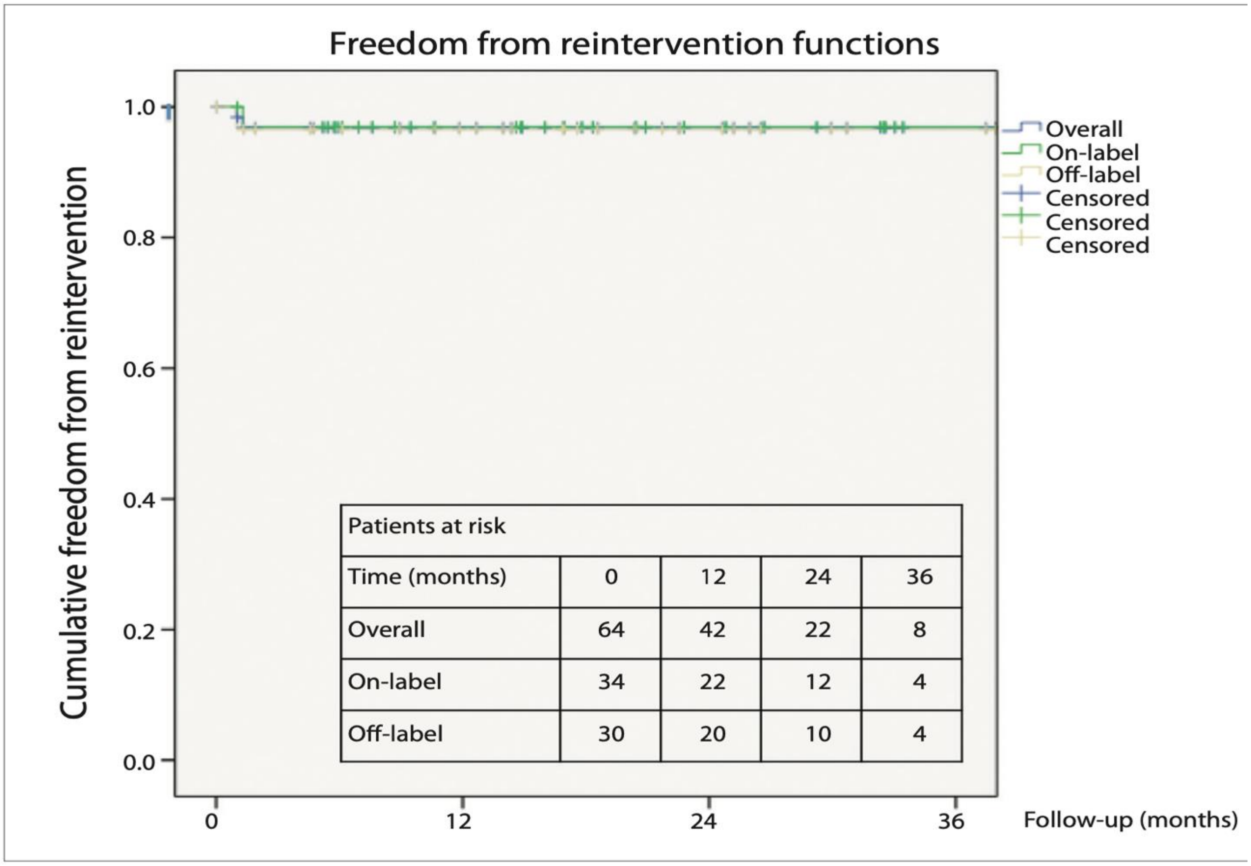

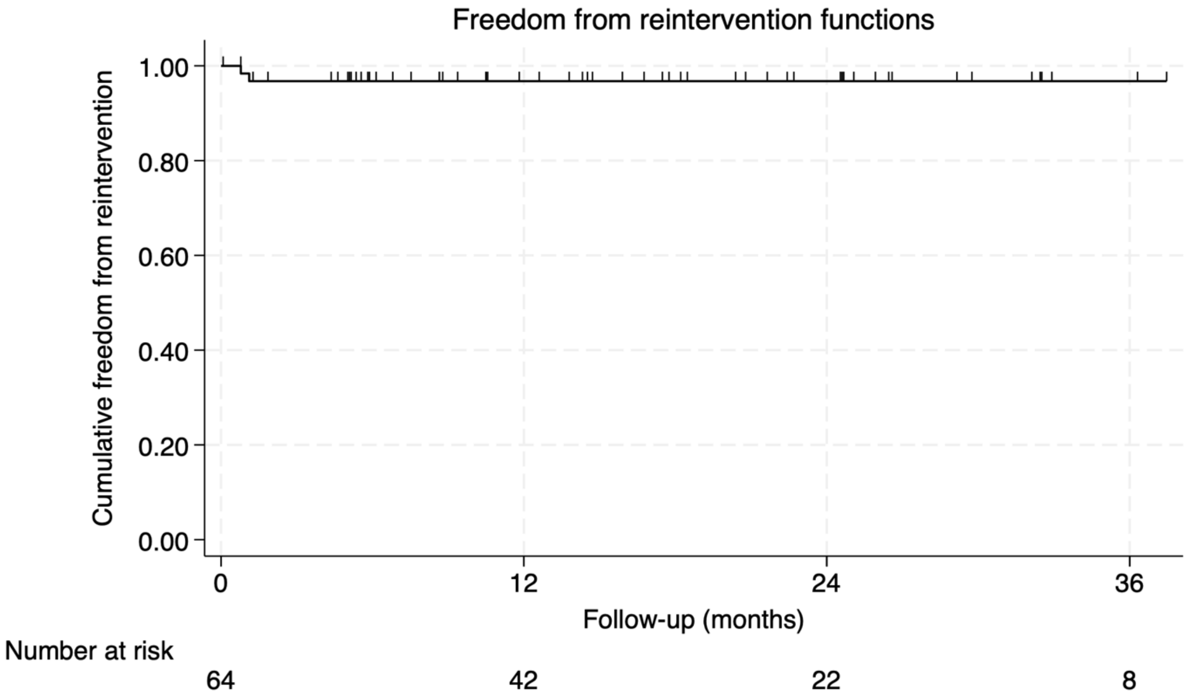

B. Regenerated KM of Salemans P.B. et al. [10]

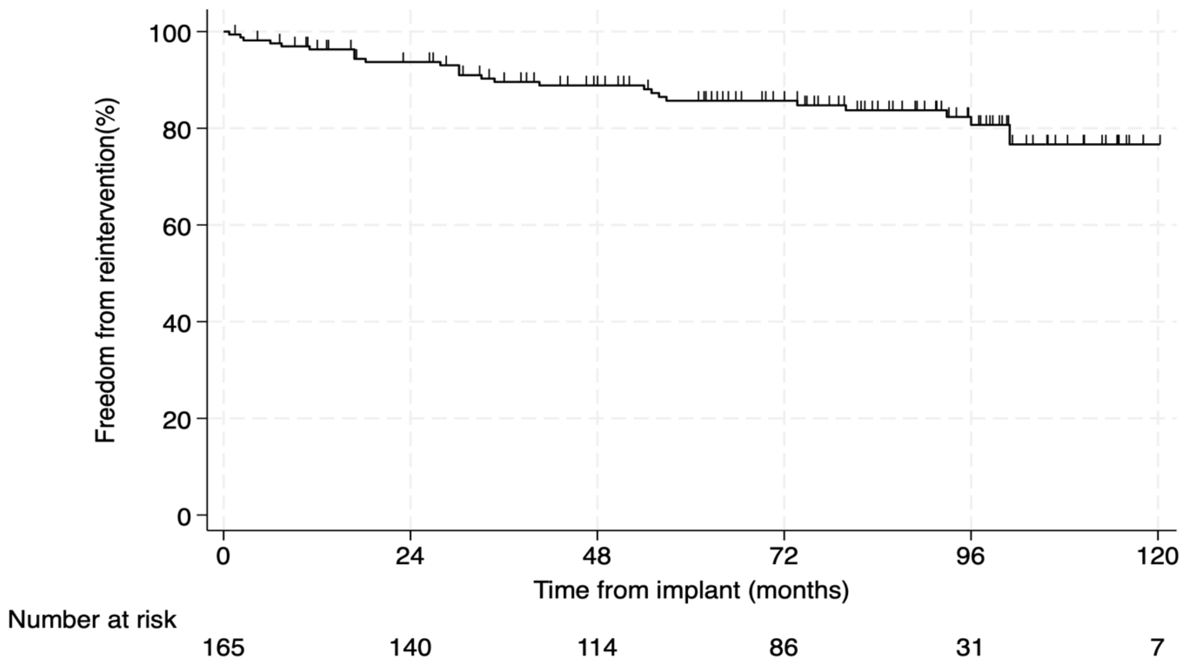

C. Original and regenerated KM of Singh M.J. et al. [44]

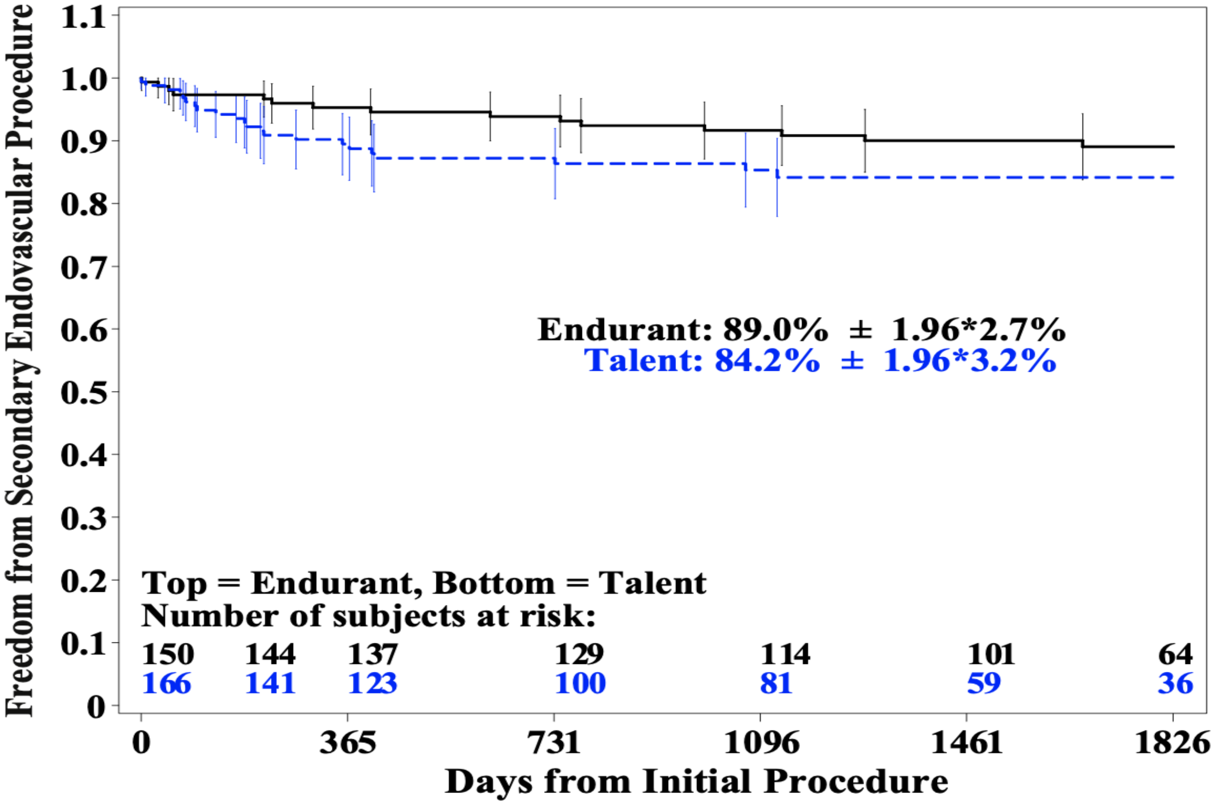

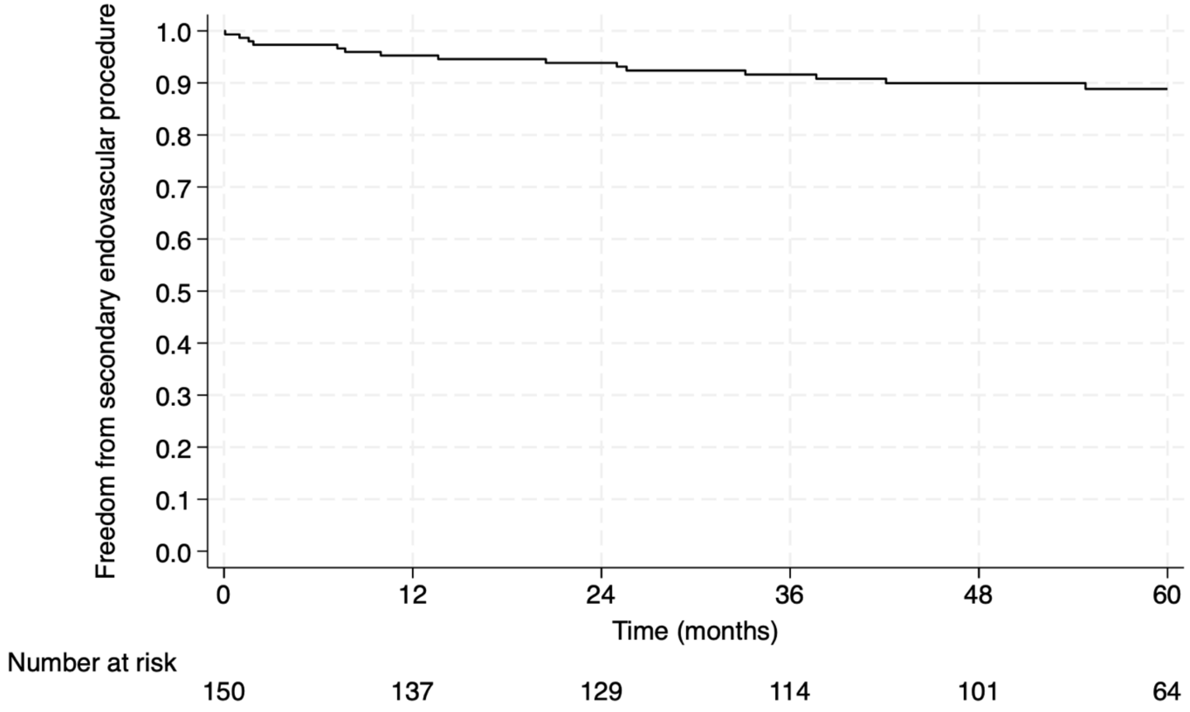

D. Original and regenerated KM of Spanos K. et al. [42]

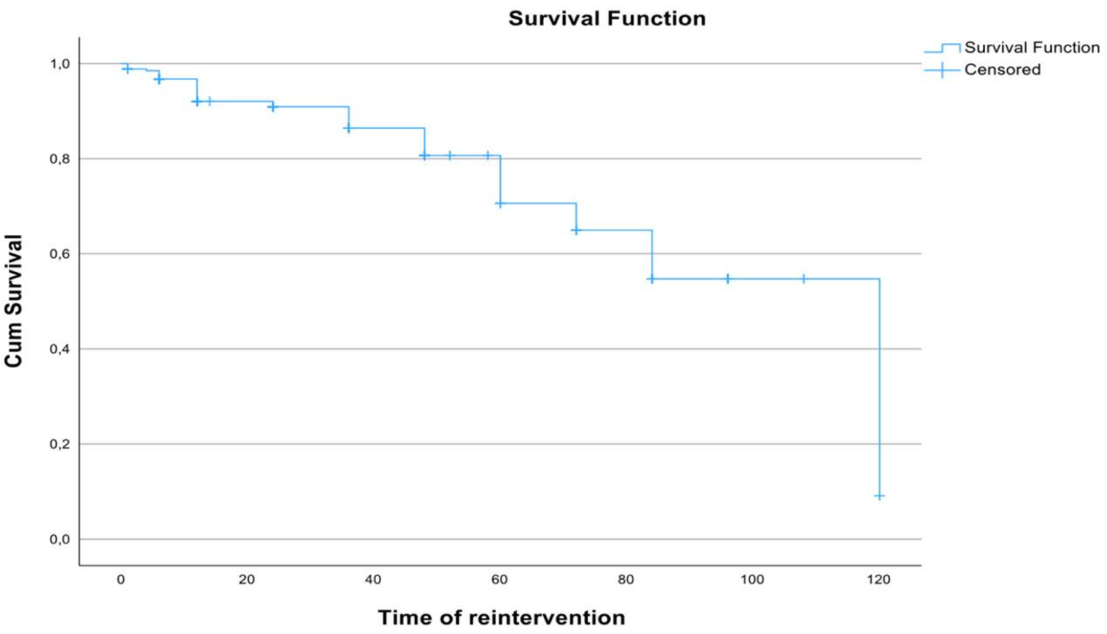

Months	12	24	36	48	60	72	84	96	108
Patients at risk	269	77	59	42	31	23	18	12	7
Events	20	20	24	27	32	33	36	36	36
Percentages	92%	-	86.4%	81%	71%	65%	55%	-	-
SE	1.7%	-	3.2%	4.4%	6.1%	6.8%	7.9%	-	-

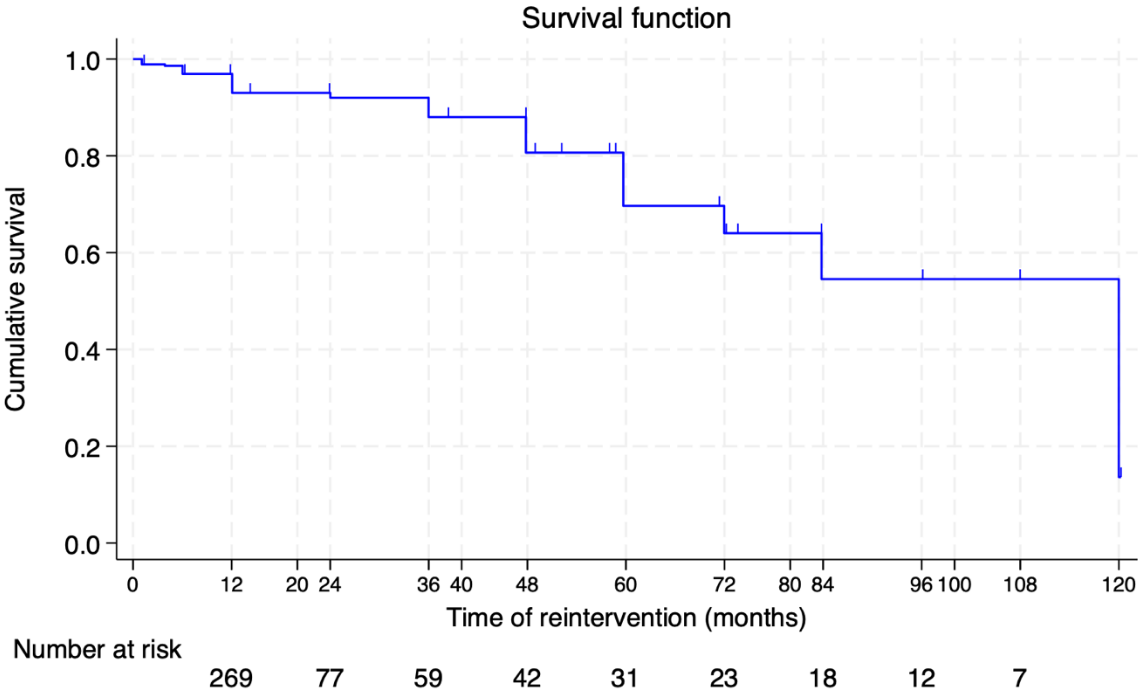

E. Original and regenerated KM of van Basten Batenburg M. et al. [32]

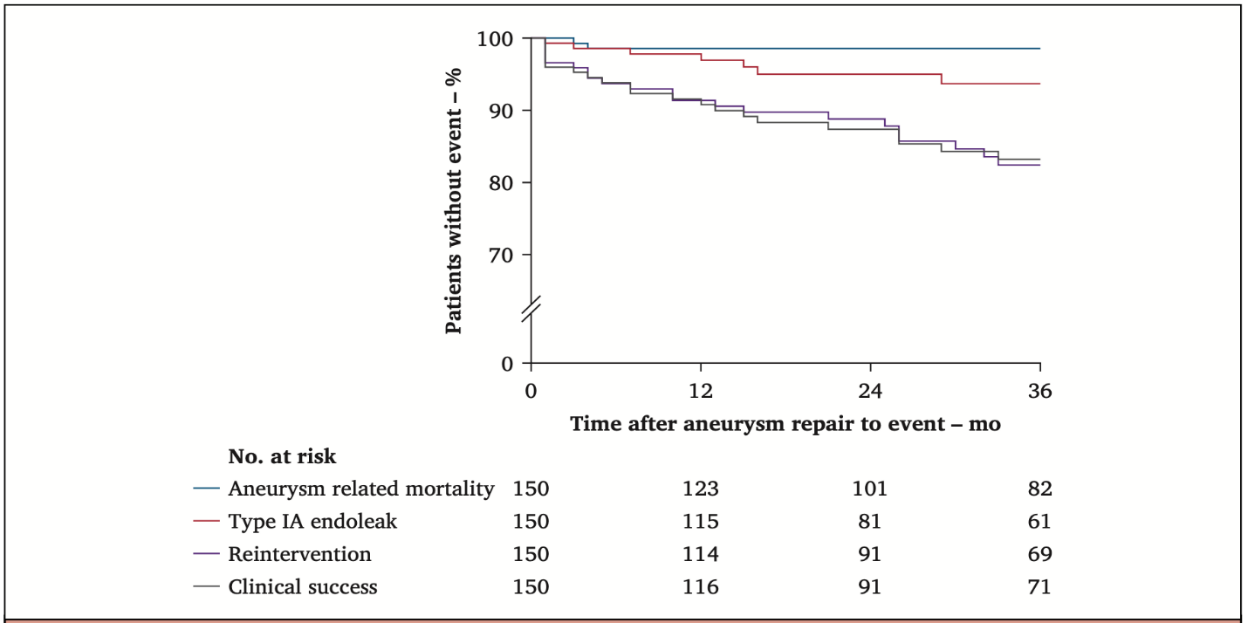

Figure 3. Cumulative Kaplan–Meier estimate of freedom from aneurysm related mortality, type IA endoleak and re-intervention, as well as clinical success in 150 patients with abdominal aortic aneurysms with challenging neck anatomy treated with endovascular aneurysm repair outside instructions for use. analysis. Note the adapted scale for the *y* axis.

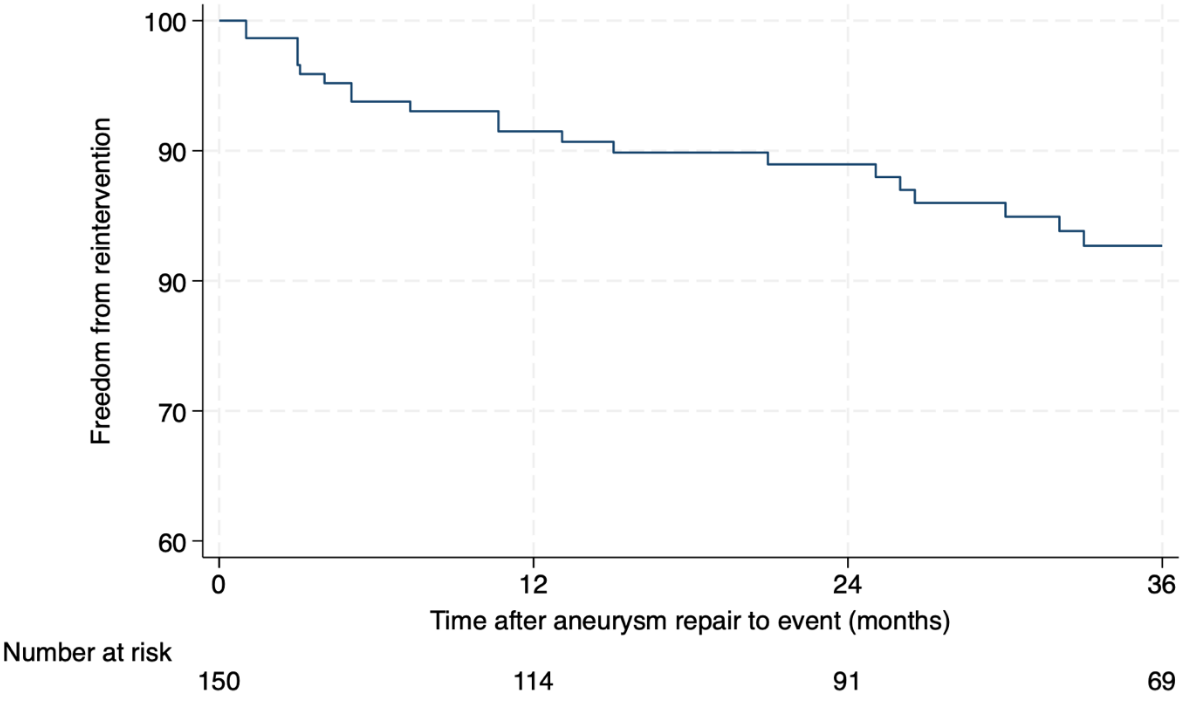

Supplemental Figure S11

A. Original and regenerated KM of Vedani S.M. et al. [40]

(The extracted data were used to reconstruct a single final KM)

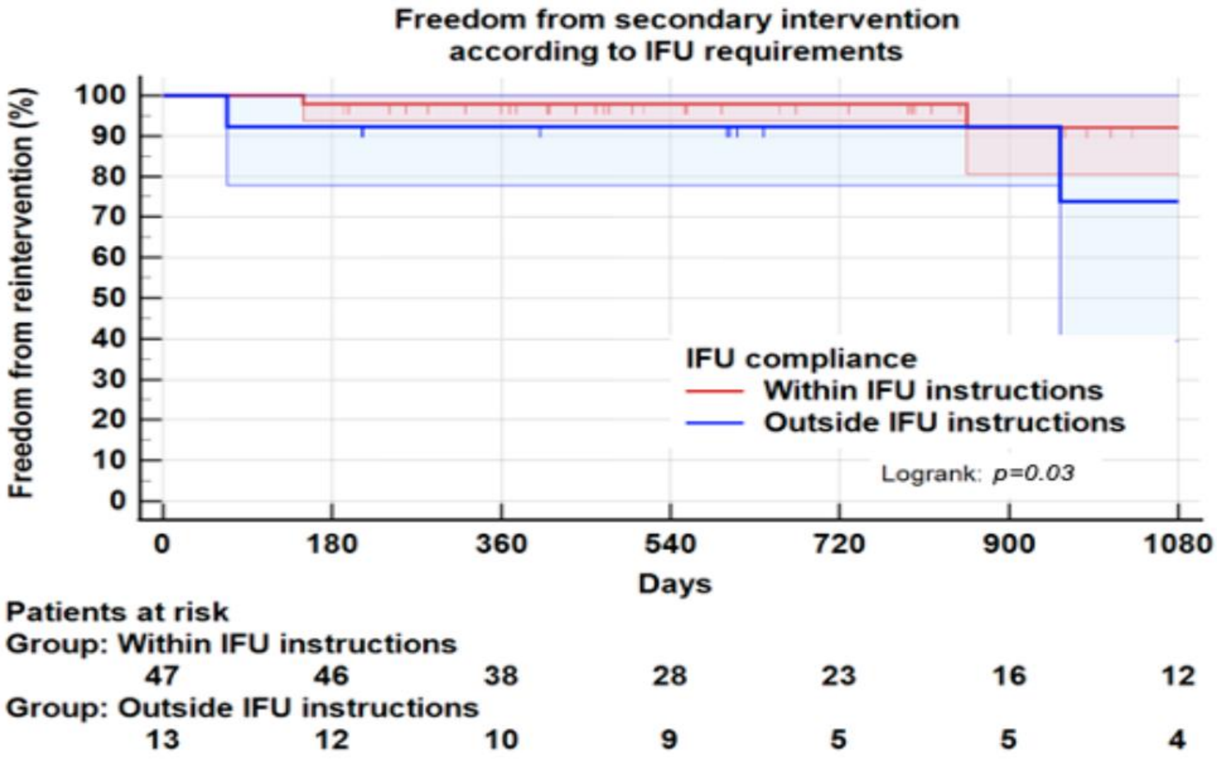

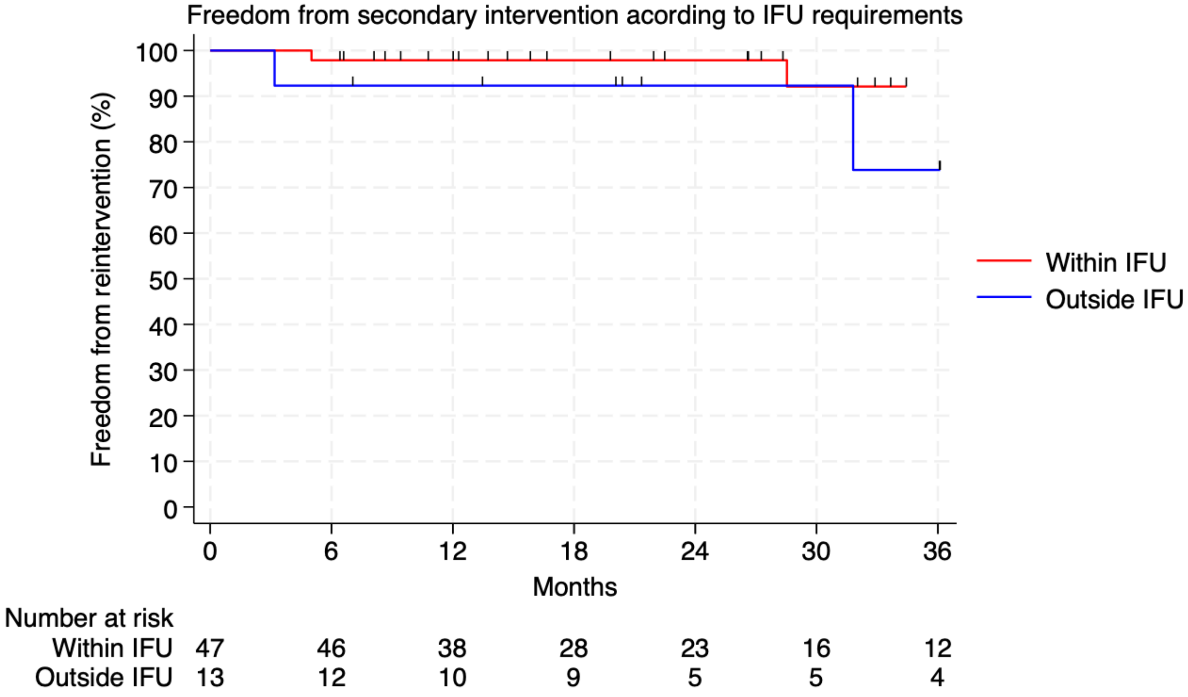

B. Original and regenerated KM of Omran S. et al. [34]

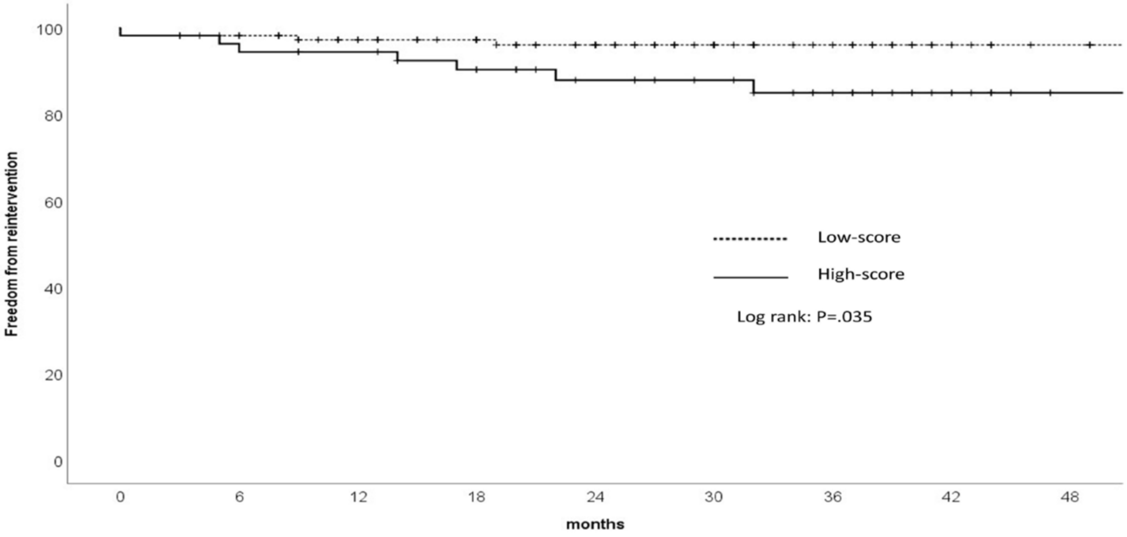

	Follow-up	0	6	12	18	24	30	36	42	48
Low-score	Pt at risk	110	106	94	87	72	55	44	32	26
	SE	.01	.02	.02	.02	.02	.02	.02	.02	.02
High-score	Pt at risk	55	51	48	43	35	31	25	18	11
	SE	.03	.03	.04	.05	.05	.05	.05	.05	.05

Figure 2. Cumulative Kaplan-Meier estimate of freedom from reintervention after endovascular procedures through to 4 years (Kaplan-Meier log-rank test, p=0.035). SE, standard error.

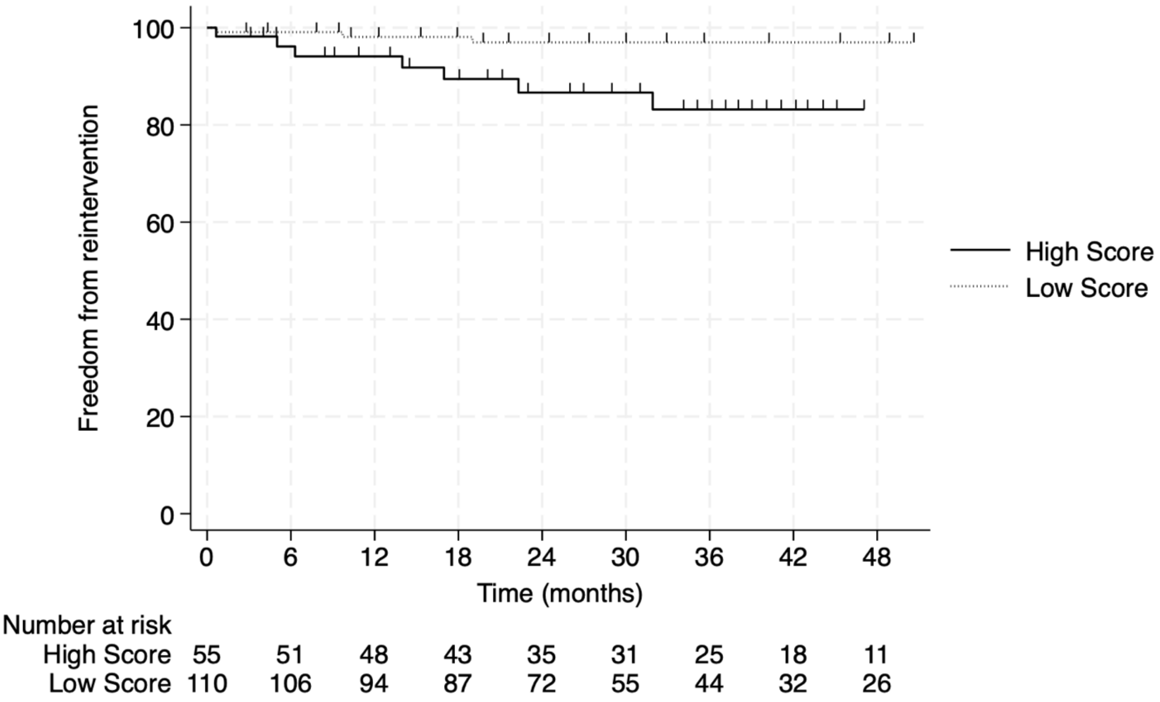

Supplement: Supplementary file 1 [file jcm-14-06453-s001.zip › Supplemental Figures S8-S11.pdf]
