# Supplementary material for: Endurant Stents in Abdominal Aortic Aneurysm Repair: A Systematic Review and Meta-Analysis
Source: J Clin Med. 2025 Sep 12;14(18):6453. doi: 10.3390/jcm14186453 (PMC12470529; doi:10.3390/jcm14186453)
Supplement: Supplementary file 1 [file jcm-14-06453-s001.zip › Supplemental Table S1.pdf]

Supplementary Table S1.
The Newcastle-Ottawa Scale was used to assess the risk of bias in the included studies. The first domain is selection, which provides up to four stars, including the representativeness of the exposed cohort, selection of the non-exposed cohort, ascertainment of exposure, and demonstration that the outcome of interest was not present at the start of the study.
The second domain is comparability, providing a maximum of two stars. As most of the included studies were not comparative, this domain was considered non-applicable (NA), except for studies that provided data comparing outcomes following the intervention within versus outside the instructions for use (IFU).
The final domain is the outcome, which evaluates the assessment of outcome, the sufficiency of follow-up length, and the adequacy of follow-up, providing up to three stars.

Author	Publication Year	Selection	Comparability	Outcome	Total
Rouwet EV et al. [28]	2011	✵✵	NA	✵✵	4
Benveniste GL et al. [29]	202	✵✵✵	✵	✵✵✵	7
Falster MO et al. [30]	2023	✵✵	NA	✵✵	4
Kvinlaug KE et al. [31]	2012	✵✵✵	NA	✵	4
van Basten Batenburg M et al. [32]	2022	✵✵✵	NA	✵✵	6
Becquemin JP et al. [33]	2021	✵✵✵	NA	✵✵✵	6
Omran S et al. [34]	2023	✵✵	NA	✵✵✵	5
Özdemir-van Brunschot DMD et al. [35]	2024	✵✵	✵	✵✵✵	6
Bisdas T et al. [36]	2014	✵✵✵	NA	✵✵	5
Troisi N et al. [37]	2014	✵✵✵	✵	✵✵	6
Deery SE et al. [38]	2019	✵✵✵	NA	✵	4
Mwipatayi B et al. [5]	2021	✵✵✵	✵	✵✵✵	7
Teijink JAW et al. [8]	2019	✵✵✵	NA	✵✵✵	6
Sekimoto Y et al. [39]	2023	✵✵	NA	✵✵	4

Vedani SM et al. [40]	2022	✵✵	✵	✵✵	5
Matsagkas M et al. [41]	2015	✵✵	✵	✵✵✵	6
Georgiadis G et al. [43]	2023	✵✵	✵	✵✵✵	6
Singh MJ et al. [44]	2016	✵✵✵	NA	✵✵✵	6
Pecorraro F et al. [45]	2016	✵✵✵	✵	✵✵	6
Setacci F et al. [11]	2014	✵✵	NA	✵✵	4
t Mannetje YW et al. [46]	2017	✵✵✵	NA	✵✵✵	6
Oliveira Pinto J et al. [47]	2019	✵✵✵	NA	✵✵✵	6
Salemans PB et al. [10]	2021	✵✵✵	NA	✵✵	5
Kemmling S et al. [48]	2022	✵✵	NA	✵✵✵	5
Spanos K et al. [42]	2024	✵✵✵	NA	✵✵✵	6
