# Supplementary material for: Endurant Stents in Abdominal Aortic Aneurysm Repair: A Systematic Review and Meta-Analysis
Source: J Clin Med. 2025 Sep 12;14(18):6453. doi: 10.3390/jcm14186453 (PMC12470529; doi:10.3390/jcm14186453)
Supplement: Supplementary file 1 [file jcm-14-06453-s001.zip › Supplemental Table S2.pdf]

Supplementary Table S2. GRADE assessment of the including studies regarding the quality of evidence.

**Outcome**	**No of studies**	**Study design**	**Risk of bias**	**Inconsistency of results**	**Indirectness of evidence**	**Imprecision**	**Publication bias**	**Overall quality**
Survival	19 [8,10,11,28–31,33–36,38–40,42–46]	7 Retrospective 12 Prospective	Serious	Serious	Not serious	Not serious	Not serious	Low
Freedom from reintervention	17 [8,10,30,32–34,36,38,40–47,49]	5 Retrospective 12 Prospective	Serious	Serious	Not serious	Not serious	Not serious	Low
Freedom from endoleak type IA	4 [8,32,33,42]	1 Retrospective 3 Prospective	Serious	Serious	Not serious	Serious	Serious	Very Low
Aneurysm related mortality	7 [32,33,35,38,43,44,46].	2 Retrospective 5 Prospectvie	Serious	Not serious	Not serious	Serious	Not Serious	Very Low
