# Supplementary material for: Endurant Stents in Abdominal Aortic Aneurysm Repair: A Systematic Review and Meta-Analysis
Source: J Clin Med. 2025 Sep 12;14(18):6453. doi: 10.3390/jcm14186453 (PMC12470529; doi:10.3390/jcm14186453)
Supplement: Supplementary file 1 [file jcm-14-06453-s001.zip › Supplemental Table S3.pdf]

Supplementary Table S3. Summary of evidence

Outcome	No of participants and studies	Quality GRADE	Relative effect 95% CI	Conclusions
Survival	5 901 patients, 19 studies [8,10,11,28–31,33–36,38–40,42–46]	Low	1 year 94.4% (94-95)<br>5 years 71.6% (70-73)<br>10 years 42.4% (37-47)	The evidence supporting the reported survival rates in patients treated with the Endurant stent endograft is of low quality
Freedom from reintervention	5 254 patients, 17 studies [8,10,30,32–34,36,38,40–47,49]	Low	1 year 94.9% (94-95)<br>5 years 81.9% (81-83)<br>10 years 43.7% (36-51)	The evidence supporting the reported freedom from reintervention rates in patients treated with the Endurant stent endograft is of low quality
Freedom from endoleak type IA	1 954 patients, 4 studies [8,32,33,42]	Very Low	1 year 98.8% (98-99)<br>5 years 94.6% (93-96)<br>10 years 85.6% (68-94)	The evidence supporting the reported freedom from type IA endoleak rates in patients treated with the Endurant stent endograft is of very low quality
Aneurysm related mortality	1 151 patients, 7 studies [32,33,35,38,43,44,46]	Very Low	1 year 0.8% (0.42-1.5)<br>5 years 2.3% (1.44-3.55)<br>10 years 7.6% (3.8-15.1)	The evidence supporting the reported aneurysm related mortality rates in patients treated with the Endurant stent endograft is of very low quality

Abbreviations: CI: Confidence interval
