# Supplementary material for: Endurant Stents in Abdominal Aortic Aneurysm Repair: A Systematic Review and Meta-Analysis
Source: J Clin Med. 2025 Sep 12;14(18):6453. doi: 10.3390/jcm14186453 (PMC12470529; doi:10.3390/jcm14186453)
Supplement: Supplementary file 1 [file jcm-14-06453-s001.zip › Supplemental Table S4.pdf]

Supplemental Table S4. Data regarding reasons for reintervention.

Study	Type Ia endoleak no (%)	Type 1b endoleak no (%)	Type II endoleak no (%)	Type III endoleak no (%)	Graft migration no (%)	Stenosis or Occlusion of iliac limb no (%)	Related to proximal neck no (%)
't Mannetje YW et al. [46]	6 (4.8)	3 (2.3)	4 (3.2)	3 (2.3)	0 (0)	10 (7.9)	6 (4.8)
Becuemin Jp et al. [33]	1 Type I (0.5)		4 (2.2)	1(0.5)	0(0)	31 (17.2)	-
Bisdas T et al. [36]	3 (1.1)	2 (0.7)	4 (1.4)	1 (0.3)	-	10 (3.6)	-
Deery Se et al. [38]	3 (1.6)	0 (0)	1 (0.5)	-	0(0)	6 (3.3)	3 (1.6)
ENGAGE [5]	-	-	-	-	-	-	-
Falster MO et al. [30]	-	-	-	-	-	-	-
Georgiadis SG et al. [43]	10 (24.4)	2 (4.9)	7 (17.1)	2 (4.9)	2 (4.9)	8 (19.5)	12 (29.3)
Kemmling S et al. [48]	2 (2)	1 (1)	8 (8)	-	0 (0)	4 (4)	-
Matsagkas M et al. [41]	0 (0)	0 (0)	0 (0)	0 (0)	0 (0)	1	0 (0)
Oliveira-Pinto J et al. [47]	8 (5.1)	-	-	-	-	-	14 (8.9)
Pecoraro F et al. [45]	0 (0)	0 (0)	-	0 (0)	-	1 (1.5)	(0)
Salemans PB et al. [10]	11 (6.7)	3 (1.8)	6 (3.6)	1 (0.6)	-	4 (2.4)	-
Sing MJ et al. [44]	1 (0.6)	1 (0.6)	7 (4.6)	0	0 (0)	6 (4)	1 (0.6)
Spanos K et al. [42]	-	-	-	-	-	14 (5.2)	-

van Basten Batenburg M et al.[32]	2 (1.3)	1 (0.6)	0 (0)	0 (0)	1 (0.6)	1 (0.6)	-
Vedani SM et al.[40]	4 (6.7)		1 (1.6)	0	0	1 (1.6)	-
Omran S et al.[34]	2 (1.2)	1 (0.6)	11 (6.7)	-	-	6 (3.6)	-
